# Genetics of suicide ideation. A role for inflammation and neuroplasticity?

**DOI:** 10.1007/s00406-024-01836-6

**Published:** 2024-06-15

**Authors:** Fabrizio Turiaco, Fiammetta Iannuzzo, Antonio Bruno, Antonio Drago

**Affiliations:** 1 Department of Biomedical and Dental Sciences and Morphofunctional Imaging, University of Messina, Via Consolare Valeria 1, 98125 Contesse, Messina Italy; 2 Unit for Psychiatric Research, Psychiatry, Aalborg University Hospital, 9100 Aalborg, Denmark

**Keywords:** SNP, Molecular pathway analysis, GWAS, PCR, Machine learning, Suicide ideation

## Abstract

Suicide is a leading cause of death worldwide. Suicide ideation (SI) is a known risk factor for suicide behaviour (SB). The current psychobiology and genetic predisposition to SI and SB are poorly defined. Despite convincing relevance of a genetic background for SI, there is no current implementable knowledge about the genetic makeup that identifies subjects at risk for it. One of the possible reasons for the absence of a clear-cut evidence is the polygenetic nature of SI along with the very large sample sizes that are needed to observe significant genetic association result. The CATIE sample was instrumental to the analysis. SI was retrieved as measured by the Calgary test. Clinical possible covariates were identified by a nested regression model. A principal component analysis helped in defining the possible genetic stratification factors. A GWAS analysis, polygenic risk score associated with a random forest analysis and a molecular pathway analysis were undertaken to identify the genetic contribution to SI. As a result, 741 Schizophrenic individuals from the CATIE were available for the genetic analysis, including 166,325 SNPs after quality control and pruning. No GWAS significant result was found. The random forest analysis conducted by combining the polygenic risk score and several clinical variables resulted in a possibly overfitting model (OOB error rate < 1%). The molecular pathway analysis revealed several molecular pathways possibly involved in SI, of which those involved in microglia functioning were of particular interest. A medium-small sample of SKZ individuals was analyzed to shed a light on the genetic of SI. As an expected result from the underpowered sample, no GWAS positive result was retrieved, but the molecular pathway analysis indicated a possible role of microglia and neurodevelopment in SI.

**Supplementary Information:**

The online version contains supplementary material available at 10.1007/s00406-024-01836-6.

## Introduction

Suicide ideation (SI) and behavior (SB) are a world-wide concern. About 800.000 individuals per year globally commit suicide. The standardized suicide rate for males and females were respectively 12 and 6/100.000 in 2019. Suicide is the second leading cause of death in younger individuals [1]. It has been estimated that the costs related to SB were in 2014 as high as 5.53 billion dollars in lost economic income with an average cost for a single suicide event estimated to be as high as 803,000 dollars [2].

The current approach to the identification and prevention of SB consists in at least three different kinds of interventions, including school-level prevention [3], brief psychological and supportive interventions [4] and follow-up interventions [5]. Exposure to suicide or suicide attempts increases the risk of suicidal behavior [6]. One of the strongest clinical predictors of SB is the non-suicidal self-injury [7]. SI and SB are favored by predisposing risk factors such as loneliness, hopelessness, demoralization, economics factors, cultural factors, or social isolation due to belonging to a social minority [8, 9].

At the moment of writing there is no implementable biologic tool to predict suicide risk in specific populations, but it is reported that the absolute risk is higher in psychiatric populations, with a suicide risk during the first year of follow-up of 1.4% compared with 0.23% in non-psychiatric populations. Evaluating the risk of SB in individual affected by major psychiatric disorders is essential to prevent this occurrence [10, 11]. In particular, one group of individuals that have higher risk for SI and SB are those suffering from Schizophrenia (SKZ). SKZ is a chronic mental disorder with a prevalence of about 1% in general population. It is characterized by positive symptoms, including hallucinations, delusions and disorders of thought that may lead to aggressive or risky behavior; negative symptoms defined by impaired speech, tendence to isolation, abulia and emotional flattening; and cognitive impairment with deficits in verbal and working memory and attention disorders [12]. Regarding the pathogenesis, SKZ has been described as a neurodevelopmental disorder in which alterations of central nervous system can appear around or before birth, as well as around the onset of psychosis. A central role has been attributed to the disruption of the neural pruning, which could lead to an excessive loss of synapses and consequently to the emergence of the illness [13].

The SB rate in SKZ ranges from 1.49% (completers) [14], to 20.3% (attempters) [15]. The RR is as high as 9.76 (completers) [16].

As for SKZ [17], both SI and SB have a genetic basis, reviewed in here [18] and here [19]. It has been reported that the genetic contribution to the development of SI and SB would range from 30 to 50% [1]. In particular, classical genetic studies reported a RR of 8.38 for biological relatives of individuals who committed suicide. Despite this, risk genes for SI and SB in SKZ have not been consistently identified [9]. The last GWAS analyses revealed although a genetic predisposition that can be determined via the polygenic risk score (PRS): SB and SI probably have a multigenic nature [20]. SKZ also have a genetic origin, this confirmed by the first twin studies reviewed here [21]and here [22] and by the last GWAS investigations in this field, see for example here [23] and here [24].

The present contribution aims at the identification of the genetic risk for suicide in individuals with SKZ. The hypothesis under analysis is that there is a combined genetic background for SI and SB in SKZ individuals. This line of research is not new, see Table 1 for an overview about a selection of the most relevant studies in the field. In the present contribution we undertake a combined approach that includes both a classic GWAS approach, a molecular pathway analysis, a polygenic risk score (PRS) and a machine learning approach to test the hypothesis that a specific set of molecular pathways or a specific genetic background may inform the risk for SI in SKZ. Due to the limits of the database under analysis it is only SI, which is taken into consideration, and not SB. SI has been reported to be a risk factor for SB: about 1 in 10 of the subjects with SI will experience SB [25].

**Table 1:** Main Previous Findings about the Genetics of Suicide Behavior in Schizophrenia

References	Sample	Main findings	Type of study
Saetre et al. (2010) [53]	n = 734 individuals with schizophrenia	No significant findings (TPH1 A218C and A779C)	Candidate gene
Fabbri & Serretti (2017) [54]	36,989 cases 113,075 controls	Significant association (IGSF9B rs75059851)	Genome-wide association study, GO enrichment, case control study
Bani-Fatemi et al. (2013) [55]	n = 241 subjects with schizophrenia, 53 suicide attempters and 188 as non-attempters	CpG rs2661319 associated with SB in SKZ	Candidate gene
Finseth et al. (2014) [56]	n = 338 individuals with schizophrenia	ITIH3/4 rs2239547 significantly associated with SB in SKZ No significant association (ANK3 rs10994359 and CACNA1C rs4765905)	Candidate gene
Carlström et al. (2012) [57]	n = 734 individuals with schizophrenia	SLC6A4 rs16965628 significantly associated with SB in SKZ	Candidate gene
Lang et al. (2019) [11]	n = 1087 inpatients with schizophrenia n = 576 healthy controls	No significant findings (TNF-alpha gene -308G > A and—1031C > T) but -1031C/T associated with age at first episode of SB	Candidate gene, case control study
Kowalczyk et al. (2020) [58]	n = 377 individuals with paranoid schizophrenia n = 524 healthy controls	HSPA1B rs539689 associated with SB in SKZ	Candidate gene, case control study
Suchanek-Raif et al. (2017) [59]	n = 388 individuals with schizophrenia, 74 patients attempted suicide n = 657 healthy controls	No significant findings ( TNFR1 rs4149576, rs4149577, and rs1860545)	Candidate gene, case control study
Wang et al. (2015) [60]	n = 1729 participants attempted suicide n = 1794 participants without a history of attempted suicide, but with a diagnosis of mental disorder n = 2398 healthy controls	HTR2A 102 C/C associated with SB in SKZ	Meta-analysis
Giegling et al. (2008) [61]	n = 167 suicide attempters, of which 35 with schizophrenia n = 92 individuals as post-mortem sample n = 312 healthy controls	No significant findings (Ers1 rs827421, rs1913474, rs1801132, rs722207, rs974276 and rs910416)	Candidate gene, case control study
De Luca et al. (2006) [62]	n = 35 individuals with schizophrenia, of which 22 patients died by suicide	No significant findings (TPH2) ( – 8396G > C/ – 473 T > A)	Candidate gene, case control
Wang et al. (2010) [63]	n = 499 individuals with schizophrenia, of which 196 patients attempted suicide	SCN8A rs10506302, rs1601012 and rs12581041 associated with SB in SKZ	Candidate gene, case control study
Fanous et al. (2009) [64]	n = 1408 subjects, including 755 with psychotic illness	No significant findings (HTR2A)	Candidate gene, case control study
Mullins et al. (2019) [65]	n = 1683 attempters n = 2946 non-attempters, all with schizophrenia	No significant findings	Genome-wide association, case control study Meta-analysis
Bani-Fatemi et al. (2016) [66]	n = 121 individuals with schizophrenia, of which 53 patients attempted suicide	No significant findings	Genome-wide association, case control study
Zai et al. (2015) [67]	n = 188 individuals with schizophrenia, of which 55 patients attempted suicide	BDNF Val66Met and DRD3 Ser9Gly interaction significantly associated with SB in SKZ	Candidate gene, case control study
Karanović et al. (2015) [68]	n = 131 individuals with schizophrenia, of which 51 patients attempted suicide	ADARB1 rs9983925 and rs4819035 and HTR2C rs6318 significantly associated with SB in SKZ	Candidate gene, case control study
Dada et al. (2021) [69]	n = 60 individuals with schizophrenia spectrum disorder	ZNF701 cg10782349 significantly associated with SB in SKZ	Genome-wide association (methylation)
Bani-Fatemi et al. (2017) [70]	n = 20 suicide attempters with schizophrenia n = 27 suicide non-attempters with schizophrenia n = 9 individuals as post-mortem sample n = 11 healthy controls	HTR2A exon I methylation rate not significantly associated with SB in SKZ	Candidate gene, case control study (methylation)
De Luca et al. (2011) [71]	The schizophrenia sample consisted of 33 triads, seven triads plus a sibling, where three of the siblings are affected, 39 diads, eight families of a proband with a single parent and a sibling where all siblings are unaffected	BDNF Val66Met not significantly associated with SB	Candidate gene, family-based study
Sokolowski et al. (2016) [72]	n = 660 suicide attempters, of which 59 with schizophrenia	No significant findings	Genome wide association, family-based study
Feldcamp et al. (2008) [73]	n = 35 individuals with schizophrenia of which 6 died by suicide n = 35 healthy controls	PPP1R1B rs907094 associated with SKZ without SB PPP1R1B rs9352 associated with SKZ and SB DARPP-32 expression differences between SKZ – SB group, SKZ non-SB group, and controls	Candidate gene, case control study
Li et al. (2017) [74]	n = 162 individuals with schizophrenia spectrum disorder	ACP1 rs300774 associated with SB	Candidate gene study
Aytac et al. (2022) [75]	n = 113 individuals with schizophrenia n = healthy controls	TNF-α − 238 G/A and TNF- α − 308 G/A not associated with SKZ TNF-α − 238 G/A associated with SB in SKZ	Candidate gene, case control study

## Materials and methods

### Sample

The sample under analysis is the NIMH CATIE sample (NIMH contract NO1 MH90001). SKZ patients were enrolled between 1/2001 and 12/2004. CATIE was a multi-phase randomized controlled trial of antipsychotic medications involving 1460 persons with SCZ followed for up to 18 months. The main focus of the CATIE investigation was to test the tolerability of a number of antipsychotic treatments in the “real world” conditions. 51% of CATIE participants donated a DNA sample. This sub-sample is the core of the present investigation. A diagnosis of SKZ was conducted according to the Structured Clinical Interview for DSM-IV (SCID).

Inclusion criteria were: (1) a diagnosis of SCZ, (2) age 18–67 years, (3) clinical decision that oral medication was appropriate, (4) adequate decisional capacity, and (5) provision of written informed consent. Exclusion criteria were: (1) a diagnosis of schizoaffective disorder, (2) mental retardation, or (3) another cognitive disorder, (4) a history of serious adverse reactions to the proposed treatments, (5) only one psychotic episode, (6) a history of treatment resistance, (7) being pregnant or breastfeeding, (8) or a serious and unstable medical condition.

### DNA sampling & cell line establishment

Peripheral venous blood samples were sent to the Rutgers University Cell and DNA Repository (RUCDR) where cell lines were established via EBV transformation. RUCDR employs stringent quality control procedures and the success rate for immortalization exceeds 99%. Sample DNA concentrations were quantified and normalized via the use of Picogreen dsDNA Quantitation Kits (Molecular Probes, Eugene, OR).

### GWAS sampling and quality control

Affymetrix 500 K “A” chipset and a custom 164 K chip created by Perlegen to provide further coverage for the original sample. For the present analysis, the genetic data available from the HIMH database were downloaded after permission. SNPs (Single Nucleotide Polymorphism) were excluded for allele frequency < 0.01 and low genotype call rate. Deviations from the Hardy–Weinberg equilibrium were accepted under a P-threshold of 0.0001. Pruning was implemented according to standard –indep-pairwise 50 5 0.5 criteria (window size in SNPs (e.g., 50), the number of SNPs to shift the window at each step (e.g., 5), the VIF threshold. The VIF is 1/(1-R^2)). Lambda values served to exclude inflation factors. To avoid genetic stratification events a principal component analysis was conducted and the first 6 components were used as covariates. Covariates were also created from the clinical study of the sample, to avoid spurious genetic associations. For each SNP the statistics of association were generated and used to conduct the analysis of quality (to calculate lambda values), to run the basic GWAS analysis along with the calculation of the PRS and to conduct the molecular pathway analysis. PRS was calculated as the sum of the weighted effect size (or beta) calculated for each SNP. The effect size (or beta) gives the strength of the statistical association of every SNP with the phenotype under analysis. This association is weighted for the number of copies of the specific SNP in each individual.

### Genetic association tests and molecular pathway analysis

Plink [26] served for the genetic association test. Single tests for association were generated for every SNP under a logistic regression model. SNPs associated with the investigated phenotype were ranked according to the p of association. SNPs showing a significant (nominal significance, not GWAS significance) (P < 0.05) association with the phenotype under analysis were selected. The genes harboring such variations were identified and were used as the input for the molecular pathway analysis. The molecular pathway analysis was conducted using the R software suit [27] through the packages Bioconductor [28] and ReactomePA [29]. The Reactome [30] is a manually curated database that includes chemical reactions, biological processes, and molecular pathways. SNPs that were used for the molecular pathway analysis were not preselected. Exonic, intronic, and SNPs from regulatory regions were entered into the analysis, allowing for a coverage of the molecular pathways as large as possible. The test for enrichment is corrected for multiple testing, considering the total number of molecular pathways that are tested for enrichment. Both the Bonferroni test (referred to as Padjust in the Results section) for multiple testing and the false discovery rate (referred to as q value in the Results section) were implemented in the analysis. The analysis was carried out in a Linux system in Bash language and the computations were performed through access at the Aarhus University superPC (https://genome.au.dk/). Polygenic risk score (PRS) was calculated and was instrumental to inform the machine learning model.

### Machine learning

A random forest (RF) method was applied to test the predictive power of a combined genetic and clinical profile. RF was chosen because it has a non-parametric capacity and can be implemented to solve the classification of two-category issues, which is the case on the present investigation. Multiple decision trees are created and combined in a single model, creating the best fitting classification based by the decision points of each decision tree. The sample was divided in a training set (70%) and a testing set (30%). Accuracy, Specificity and Sensitivity (Confusion Matrix) were used to describe the model.

### Definition of the principal outcome

The outcome under analysis is SI as measured by the Calgary scale [31], item number 8. Suicide ideation is explored at each interview and ranked according to the level of intensity as “Absent”, “Mild”, “Moderate” and “Severe”. The single individuals were classified as “cases” (did experience suicide ideation during the trial) and “controls” (did not experience suicide ideation during the trial).

### Analysis of covariates

The following clinical covariates were taken into consideration: age, gender, race, employment, marital status, years of education, years of treatment, age at onset and medicine. The clinical variables that were significantly associated with the outcome were included in the genetic analysis as possible stratification factors. The intensity of psychotic symptoms was also analyzed in a nested regression model as a possible confounding factor. The possible genetic stratification was analyzed with the principal component analysis in plink and the first 6 components were included in the genetic analysis.

Result.

### Sociodemographic variables

741 individuals from the CATIE study had both the genetic information and the clinical information to complete the present analysis. 274 individuals reported a positive SI at least once during the trial, while 467 individuals did not report it. The sociodemographic characteristics of the sample are reported in Table 2. Briefly, 196 females and 545 males were included, the mean age at the moment of enrolling was 40.89 ± 11.05 years. The mean age at onset and the mean duration of treatment were respectively 14.3 ± 10.83 years and 16.69 ± 11.2 years. Multiple ethnic groups were included in the analyses. White individuals were the most represented group (n = 489), followed by Black or African-American (n = 221). Most part of the enrolled individuals did not have a job (n = 627) at the moment of enrolling, the most part of them had though achieved a GED/High school diploma (n = 252) or at least some college (n = 178) level of education. The most part of the analyzed individuals were never married (n = 441). The covariate analysis for the sociodemographic variables showed that “RACE” as defined in the database was significantly associated with SI: the most relevant result was retrieved for the “Black” as defined in the database population. Individuals with SI in the “Black” group were largely less represented with respect to those without SI (0,36% vs 33%). This variable was then included in the covariate analysis for the genetic association tests. A nested mixed effect regression analysis was instrumental to test the impact of medicines (“DRUGTERM2” in the database) on suicidal ideation in the database. As reported in Table 2, “RACE” and “MARITAL”, indicating respectively the ethnicity and the marital status, were used as the clinical covariates for the genetic analysis.

**Table 2:** Clinical characteristics of the sample under analysis

Variable	Distribution	Distribution according to suicide ideation	Statistics of distribution according to suicide ideation
Age	40.89 ± 11.05	ideation = 39.53 ± 10.67 no_ideation = 41.69 ± 11.2	n.s
Gender	Female n = 196(26.45%) Male n = 545(73.55%)	ideation Variable n(%) Female n = 67(24.45%) Male n = 207(75.55%) no_ideation Variable n(%) Female n = 129(27.62%) Male n = 338(72.38%)	n.s
Marital Status	Divorced n = 163(22%) Married n = 79(10.66%) Never married n = 441(59.51%) Separated n = 43(5.8%) Widowed n = 15(2.02%)	ideation Variable n(%) Divorced n = 51(18.61%) Married n = 31(11.31%) Never married n = 164(59.85%) Separated n = 23(8.39%) Widowed n = 5(1.82%) no_ideation Variable n(%) Divorced n = 112(23.98%) Married n = 48(10.28%) Never married n = 277(59.31%) Separated n = 20(4.28%) Widowed n = 10(2.14%)	MARITALMarried t-test = -0.105; P value = 0.916403 MARITALNever married t-test = – 0.017; P value = 0.986501 MARITALSeparated t-test = 2.705; P value = 0.007014 MARITALWidowed t-test = 0.049; P value = 0.96071
Race	NA n = 1(0.13%) Asian Alone n = 14(1.89%) American Indian n = 4(0.54%) Black n = 221(29.82%) Native Hawaiian n = 1(0.13%) Two or more races n = 11(1.48%) White alone n = 489(65.99%)	ideation Variable n(%) Asian Alonen = 1(0.36%) American Indian n = 6(2.19%) Black n = 1(0.36%) Native Hawaiian n = 67(24.45%) Two or more races = 3(1.09%) White alone n = 196(71.53%) no_ideation Variable n(%) Asian Alone n = 8(1.71%) American Indian n = 3(0.64%) Black n = 154(32.98%) Native Hawaiian n = 1(0.21%) Two or more races n = 8(1.71%) White alone n = 293(62.74%)	RACEAmerican Indian or Alasks Native alone t-test = – 4.136; P value = 4e-05 RACEAsian alone t-test = – 4.159; P value = 3.6e-05 RACEBlack or African-American alone t-test = – 4.591; P value = 5e-06 RACENative Hawaiian or Other Pasific Islander alone t-test = – 3.288; P value = 0.001051 RACETwo or More Races t-test = – 4.415; P value = 1.2e-05 RACEWhite alone t-test = – 4.391; P value = 1.3e-05
Education	Advanced degree completed [e.g. Ph.D., M n = 1(0.13%) Advanced degree courses, not graduated [ n = 1(0.13%) College graduate n = 50(6.75%) College graduate and some Master's level n = 6(0.81%) Community college or technical school de n = 45(6.07%) Did not complete high school n = 197(26.59%) GED/High school diploma n = 252(34.01%) Master's degree completed n = 11(1.48%) Some college, did not graduate n = 178(24.02%)	ideation Variable n(%) College graduate n = 15(5.47%) College graduate and some Master's level n = 2(0.73%) Community college or technical school de n = 24(8.76%) Did not complete high school n = 64(23.36%) GED/High school diploma n = 103(37.59%) Master's degree completed n = 7(2.55%) Some college, did not graduate n = 59(21.53%) no_ideation Variable n(%) Advanced degree completed [e.g. Ph.D., M n = 1(0.21%) Advanced degree courses, not graduated [ n = 1(0.21%) College graduate n = 35(7.49%) College graduate and some Master's level n = 4(0.86%) Community college or technical school de n = 21(4.5%) Did not complete high school n = 133(28.48%) GED/High school diploma n = 149(31.91%) Master's degree completed n = 4(0.86%) Some college, did not graduate n = 119(25.48%)	n.s
Years of education	12.12 ± 2.24	ideation = 12.25 ± 2.1 no_ideation = 12.05 ± 2.32	n.s
Employment	Did Not Work n = 627(84.62%) Full Time n = 43(5.8%) Part Time n = 68(9.18%) Unknown n = 3(0.4%)	deation Variable n(%) Did Not Work n = 231(84.31%) Full Time n = 14(5.11%) Part Time n = 29(10.58%) no_ideation Variable n(%) Did Not Work n = 396(84.8%) Full Time n = 29(6.21%) Part Time n = 39(8.35%) Unknown n = 3(0.64%)	n.s
Years of treatment	16.69 ± 11.2	ideation = 15.97 ± 11.2 no_ideation = 17.12 ± 11.19	n.s
Years at onset	14.3 ± 10.83	ideation = 13.33 ± 10.79 no_ideation = 14.87 ± 10.82	n.s
PANSS	67.66 ± 18.02	No suicidal ideation = 66.83 ± 17.91 Mild suicidal ideation = 73.69 ± 17.34 Moderate suicidal ideation = 77.09 ± 17.76 Severe suicidal ideation = 67.76 ± 21.42	n.s

### Genetic and clinical variables

**Fig. 1:**
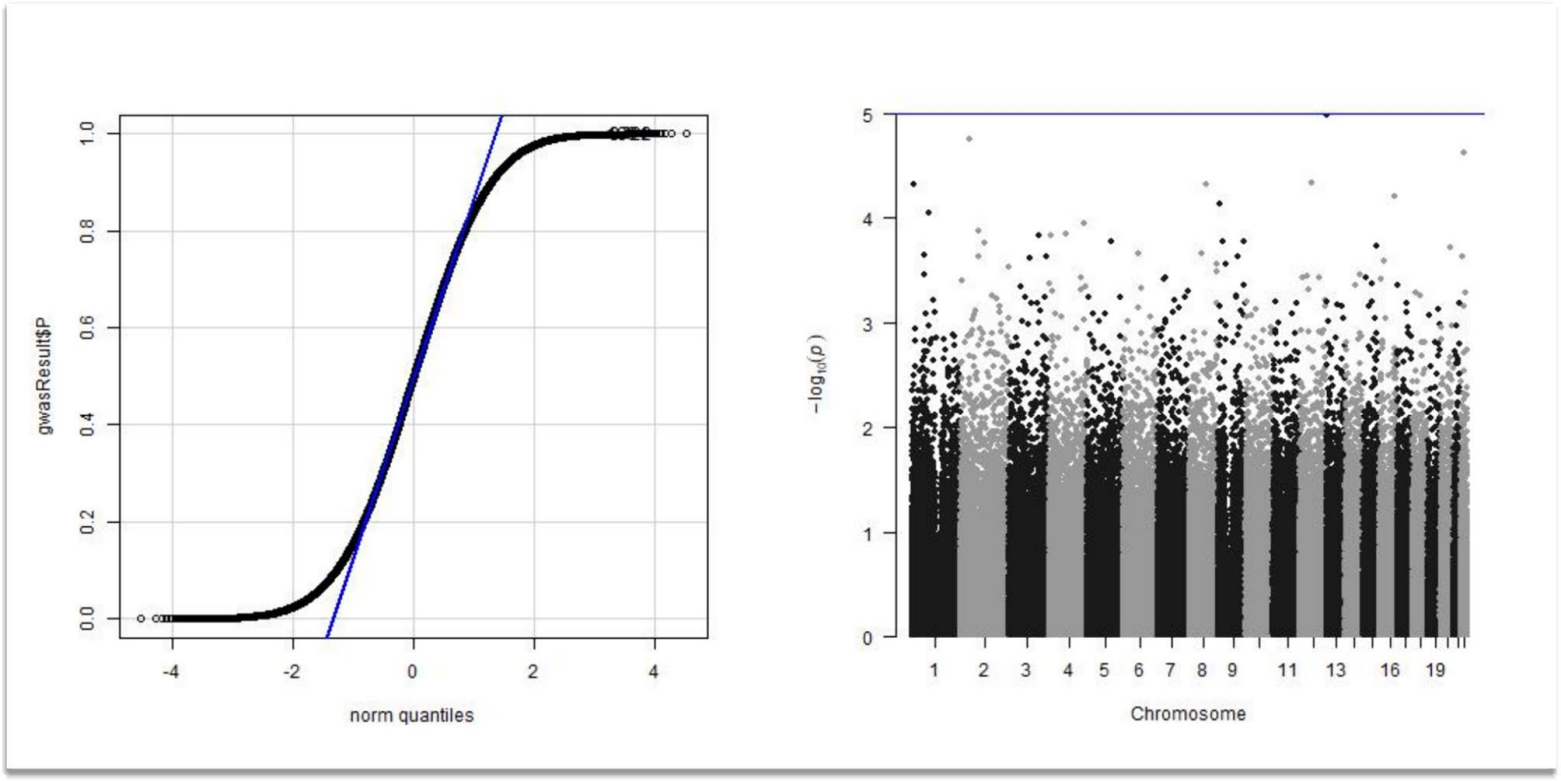
GWAS analysis. A QQ plot analysis showing no inflation factor after correction for the clinical and genetic covariates along with the Manhattan plot of the GWAS analysis showing no SNPs reaching the GWAS significance level

Of the initial 486,935 SNPs available in the genetic database, 166,325 passed the quality analysis because of low allelic representation in the database or because of unbalance in the HWE test. Of the initial database, 322,851 SNPs were excluded after the pruning analysis. The genetic database was not imputated because of the low quality of the imputation analysis in this specific dataset. Pairwise identity-by-state distance clustering allowed for the introduction of the first six dimensions of the pairwise identity-by-state distance structure as covariates for the genetic analysis. The analysis of genetic and clinical covariates resulted in a lambda value as large as 1.01, indicating no inflation factor in the final genetic analysis. The QQ-plot showing the distribution of the observed VS expected P values and the Manhattan plot are represented in Fig. 1.

None of the SNPs investigated achieved a genome-wide significant association with the outcomes under analysis. The following molecular pathways were found to be significantly associated with the outcome under analysis after correction for multiple testing: (1) Protein–protein interactions at synapses; (2) Neurexins and neuroligins; (3) O-linked glycosylation; (4) Glucuronidation; (5) Neuronal System; (6) Defective B3GALTL causes PpS; (7) cGMP effects; (8) Collagen chain trimerization; (9) O-glycosylation of TSR domain-containing proteins; (10) Nitric oxide stimulates guanylate cyclase; (11) Non-integrin membrane-ECM interactions and (12) NCAM1 interactions. The result of the molecular pathway analysis is detailed in Table 3.

**Table 3:** Molecular pathway analysis result

ID	Description	GeneRatio	BgRatio	p value	p.adjust	qvalue	geneID	Count
R-HSA-6794362	Protein–protein interactions at synapses	27/903	86/10955	5.29E-10	6.99E-07	6.68E-07	*APBA2/DLG2/GRIA4/GRIN2A/GRM1/GRM5/PTPRD/PTPRF/PTPRS/SYT1/PPFIBP1/LIN7A/DLGAP1/NRXN3/NRXN1/HOMER2/FLOT1/PDLIM5/IL1RAPL1/DLGAP4/SHANK2/IL1RAPL2/NLGN4X/DLGAP3/LRRTM4/SYT2/SYT9*	27
R-HSA-6794361	Neurexins and neuroligins	19/903	55/10955	3.40E-08	2.25E-05	2.15E-05	*APBA2/DLG2/GRIN2A/GRM1/GRM5/SYT1/LIN7A/DLGAP1/NRXN3/NRXN1/HOMER2/PDLIM5/DLGAP4/SHANK2/NLGN4X/DLGAP3/LRRTM4/SYT2/SYT9*	19
R-HSA-5173105	O-linked glycosylation	24/903	111/10955	9.28E-06	0.004	0.0039	*GALNT2/ST3GAL1/ST3GAL3/LARGE1/ADAMTS7/ADAMTS6/GALNT10/C1GALT1/GALNT16/GALNT17/GALNT14/THSD4/ADAMTS20/THSD7B/ADAMTS12/ADAMTSL1/GALNT13/ADAMTS16/ADAMTS17/ADAMTS18/ADAMTS19/ST6GALNAC3/GALNT18/GALNTL6*	24
R-HSA-156588	Glucuronidation	10/903	25/10955	1.43E-05	0.004	0.004	*UGT1A10/UGT1A8/UGT1A7/UGT1A6/UGT1A5/UGT1A9/UGT1A4/UGT1A3/UGT2A3/UGT3A1*	10
R-HSA-112316	Neuronal System	57/903	410/10955	5.75E-05	0.015	0.014	*AP2A2/APBA2/CACNA1E/CACNA2D1/CACNB2/CHRNA7/CREB1/DLG2/ERBB4/GABRA2/GABRB2/GLRA2/GRIA4/GRIK1/GRIK2/GRIK4/GRIN2A/GRM1/GRM5/KCNH1/KCNJ12/KCNMA1/KCNQ1/KCNQ3/KRAS/MYO6/PRKAA1/PRKCA/PTPRD/PTPRF/PTPRS/SLC6A3/SYT1/KCNAB1/PPFIBP1/LIN7A/HTR3B/DLGAP1/NRXN3/NRXN1/HOMER2/FLOT1/PDLIM5/IL1RAPL1/DLGAP4/SHANK2/ARHGEF9/PLCB1/IL1RAPL2/NBEA/PRKAG2/CACNA2D3/NLGN4X/DLGAP3/LRRTM4/SYT2/SYT9*	57
R-HSA-5083635	Defective B3GALTL causes PpS	11/903	37/10955	1.30E-04	0.024	0.023	*ADAMTS7/ADAMTS6/THSD4/ADAMTS20/THSD7B/ADAMTS12/ADAMTSL1/ADAMTS16/ADAMTS17/ADAMTS18/ADAMTS19*	11
R-HSA-418457	cGMP effects	7/903	16/10955	1.49E-04	0.024	0.023	*ITPR1/KCNMA1/PDE1A/PRKG1/PDE5A/PDE10A/PDE11A*	7
R-HSA-8948216	Collagen chain trimerization	12/903	44/10955	0.000162119	0.024	0.023	*COL1A1/COL4A1/COL4A2/COL4A3/COL6A3/COL8A1/COL9A1/COL9A3/COL13A1/COL25A1/COL23A1/COL26A1*	12
R-HSA-5173214	O-glycosylation of TSR domain-containing proteins	11/903	38/10955	0.000169391	0.024	0.023	*ADAMTS7/ADAMTS6/THSD4/ADAMTS20/THSD7B/ADAMTS12/ADAMTSL1/ADAMTS16/ADAMTS17/ADAMTS18/ADAMTS19*	11
R-HSA-392154	Nitric oxide stimulates guanylate cyclase	8/903	22/10955	0.000231871	0.028	0.027	*GUCY1A2/ITPR1/KCNMA1/PDE1A/PRKG1/PDE5A/PDE10A/PDE11A*	8
R-HSA-3000171	Non-integrin membrane-ECM interactions	14/903	59/10955	0.000239362	0.028	0.027	*ACTN1/COL1A1/COL4A1/COL4A2/COL4A3/DMD/HSPG2/ITGB4/LAMA3/LAMC2/DDR2/PRKCA/NRXN1/LAMC3*	14
R-HSA-419037	NCAM1 interactions	11/903	42/10955	0.000444033	0.028	0.027	*CACNA1C/CACNA1D/CACNB2/COL4A1/COL4A2/COL4A3/COL6A3/COL9A1/COL9A3/NCAM1/CACNA1I*	11

The machine learning approach to the analysis was conducted to the application of a random forest technique, which was deemed appropriate for the “case VS controls” phenotype under analysis. The initial database was split in 400 individuals as the training test, and 351 individuals for the test sample. As a result, a random forest of 20 tress was enough to obtain stable result from the OOB (out of Bag) result, with an OOB estimate error of 0.88%. The confusing matrix reported in Table 4 suggests that the model was highly predictive of the final classification in the test sample. The mtry parameter was set at 5, allowing for 5 variables to be included in the model at each node. The predictive variables used in the random forest analysis were the PRS as calculated in the training test, age, gender, ethnicity, marital status, years at presentation and years of education. The analysis of the single weight of the genetic and clinical variables in predicting the final classification was unbalanced towards the genetic component of the analysis.

**Table 4:** Confusion matrix from the Random Forest Analysis

	Oberved cases	Observed controls
Predicted cases	220	1
Predicted controls	2	117

## Discussion

Suicide ideation, known also as suicidal thoughts, is a phrase used to depict preoccupations and whishes linked to the idea of killing oneself [25]. There is at the moment of writing no consensus on a gold standard to assess SI [32] and the psychodiagnostics tools commonly used to assess it are not effective in identifying people at imminent risk of suicide [33, 34]. SI is considered a better predictor of lifetime risk for suicide than imminent risk [35] and can be distinguished in "active suicidal ideation”, characterized by specific suicidal ideas, with plans aiming to get death [36]; and “passive suicidal ideation”, described as a general desire do die, without any specific plan or mean to give oneself death [37]. There is evidence reporting that up to 75% of patients who died by suicide denied SI in the last month before acting suicide[35, 38].

**Fig. 2:**
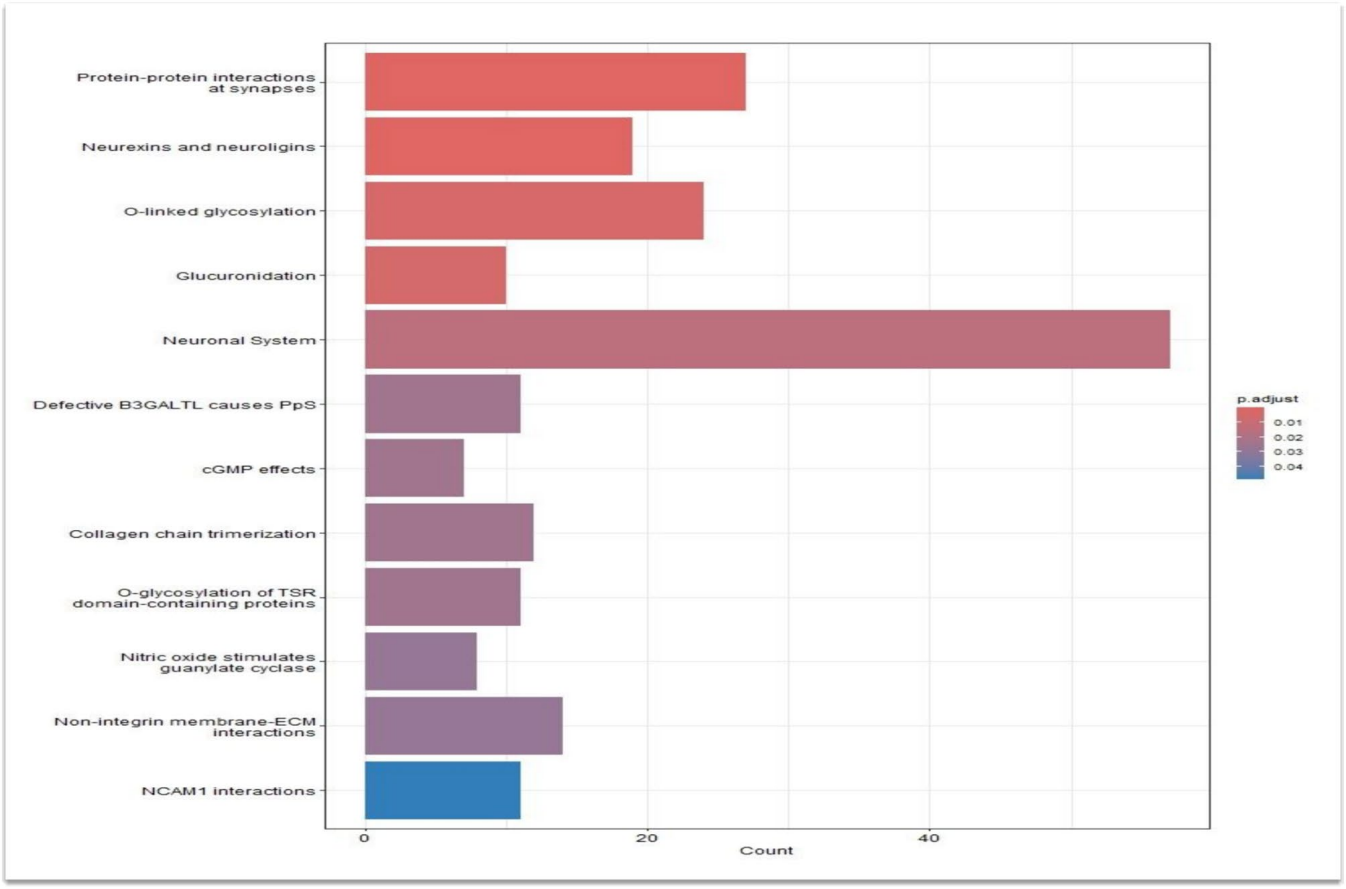
Molecular pathway analysis

SCZ patients with SI have over a six-fold increase of suicide [39], while people who had at least one lifetime psychotic event has double odds of experiencing SI, triple odds of a future suicide attempt, and four times the odds of dying by suicide [11]. Given those data, the importance of identifying biological and genetic biomarkers emerges. Our study tried to reach this aim using a classic GWAS approach, a molecular pathway analysis and modelling the polygenic risk score with the clinical predictors in a model through machine learning. No SNP alone reached a GWAS significance in identifying the genetic risk for SI. This result may depend on the underpowered sample, which does not allow to statistically distinguish the noise signal from the true association findings. This negative association result is also explained by the probable biologic complex and most likely polygenic nature of SI. To tackle the latter hypothesis a molecular pathway analysis was undertaken, along with a random forest analysis (machine learning), that considered both the PRS – which includes all the SNPs associated with the phenotype under analysis –. As a main result, a likely overfitting random forest model was retrieved from the analysis, with an error rate in the test sample as low as < 1%. This finding is to be taken with extreme cautiousness, since overfitting is a known possible bias of random forests [40] and it is unlikely that the model under analysis reaches such high level of accuracy. On the other hand, the molecular pathway analysis revealed some significant and interesting result. Table 3 and Fig. 2 report the result of the molecular pathway analysis. Some molecular pathways that resulted to be significantly enriched in variations associated with the outcome under analysis are closely related to the neurological function such as the “Protein–protein interactions at synapses”, “Neuronal system” or “Neurexins and neuroligins”. It is relevant to stress that the second pathway, that related to neurexins and neuroligins was previously found to be associate with cognitive functions [41, 42]. A recent meta-analysis conducted by Qingqin and colleagues [20], consistently reported that one SNP located in the neuroligin 1 (NLGN1) gene was significantly associated with suicide death and suicidal behavior in a sample combining 3765 cases and 6572 controls. The relevance of genetic variants located in the neurorexin 1 towards the suicide risk, was also recently reported by William and colleagues [43]. Negative association findings are also reported [44], this being possibly related to the power of the studies (small sample sizes) reporting negative association result. Overall, it can be underlined that the molecular pathway analysis as conducted in the present investigation is a hypothesis – free strategy to interrogate the whole genome while taking advantage of the current knowledge about the known molecular cascades. It is of note, that out of a hypothesis free approach, three of the molecular pathways resulting to be enriched in SNPs associated with SI are related to the function of neurons. This finding can help to defend the robustness of our investigation.

Pathways related to glycosylation and glucuronidation were also reported to be significantly enriched in SNPs associated with the phenotype under analysis. These pathways are involved in a fan of different biologic events, and it is difficult to retrieve their specific role in determining the suicide risk. One pathway classically related to glycosylation was recently reported to play a role in the genetics of depression: *B3GALTL (Beta 3-Glucosyltransferase)* was found to be associated with suicide behavior in a proteome-wide association study on depression. The integration of data from 500,199 individuals with genome-wide data and 376 human brain proteomes identified 19 genes associated – and being causal of – depression. One of the proteins that were found to be associated with depression was the product of the *B3GALTL*. *B3GALTL* gene product codes for a protein that is implicated in synaptogenesis [45]. Quite interestingly, *B3GALTL* is expressed in the brain by neurons and astrocytes (proteinatlas.org). It is also expressed in T cells. This finding is then of particular relevance: Microglia are the primary immune system cells in the central nervous system, and they function like peripheral macrophages, releasing a multitude of pro-inflammatory cytokines and chemokines. Post-mortem examination of suicide patients shows an increased density of microglia in anterior cingulate cortex, dorsolateral prefrontal cortex, and mediodorsal thalamus regions [46]. An increased microglial activation is identified by PET in patients with SI [47]. Microglial cells affect the tryptophan-kynurenine pathway, increasing the production of neurotoxic metabolites such as quinolinic acid, a NMDA agonist [46] and creating an imbalance in the glutamatergic system. Glutamate neurotransmission is the background of a normal cognitive and emotional processing, so its disruption can lead to increased impulsivity, depressed mood, and suicidality [48]. Consistently with this finding, another important molecule involved in neuroinflammation is the 18-kDa Translocator Protein (TSPO), whose expression was significantly increased in patients with SI, most robustly in the regions of the anterior cingulate cortex [47]. Refer to Table 5, for previous relevant studies about schizophrenia, neuroinflammation and SI.

**Table 5:** Main Previous Findings about the Genetics of Neuroinflammation and SB in SKZ

References	Outcomes	Main findings	Type of study
Shinko et al. (2020) [76]	CCL1, CCL8, CCL13, CCL15, CCL17, CCL19, CCL20, CCL22, CCL25, CX3CL1, CXCL11, CXCL16, IL-10, IL-16, MIF	CCL1, CCL8, CCL13, CCL15, CCL17, CCL19, CCL20, CXCL11, and IL-10 were significantly decreased and IL-16 significantly increased in suicide completers	Case control study
Ermakov et al. (2023) [77]	CXCL8/IL-8, CCL2/MCP-1, CCL4/MIP-1β, CCL11/eotaxin-1 CX3CR1 CCL5 CCL2 CXCR4	Increased levels of CXCL8/IL-8, CCL2/MCP-1, CCL4/MIP-1β, CCL11/eotaxin-1 in the blood of patients with SCZ Increased CX3CR1 mRNA expression in SCZ without suicide as compared to suicide completers	Systematic review
Purves‐Tyson et al. (2021) [78]	Eight GABAergic‐related transcripts Glutamate decarboxylase (GAD) 65/67 GABAA alpha 3 (α3) (GABRA3) protein	GAD1 mRNA levels were significantly higher in SCZ died by suicide compared to SCZ died by non-suicide causes	Case control study
Suzuki et al. (2021) [46]	Microglial activation	HLA-DR as a microglial marker in suicide	Review
Rahimian et al. (2021) [79]	Microglial phenotype and function	Increased mRNA and protein levels of IL-1β, IL-6, and TNF-α in suicide Increased CX3CR1 mRNA expression in SZ without suicide as compared to suicide victims Elevated P2Y purinoceptor 12 (P2RY12) mRNA in suicide completers Reduced TREM2 expression in non-suicide subjects with SZ	Review
Trépanier et al. (2016) [80]	Microglial activity	HLA-DR overexpressed in SCZ suicide	Review
Brisch et al. (2022) [81]	Microglial activity and neuroinflammation	Decreased quinolinic acid levels in SCZ patients	Review

A pathway related to the Nitric oxide (NO) function was also found to be enriched in SNPs associated with the outcome under analysis. NO has many functions in the human body [49]. It regulates vascular tone and blood flow by activating soluble guanylate cyclase (sGC) in the vascular smooth muscle, and it controls mitochondrial O2 consumption by inhibiting cytochrome c oxidase. Of note, NO is also used as a regulator of the metabolic state of neurons. Our result is consistent with previous findings in literature. Baltazar-Gaytan reported that reduced NO levels were found in the anterior-pituitary region of young suicide completers [50]. The relevance of this pathway as a possible mediator between early trauma and suicide behavior [51].

Neurodevelopment may be the physiologic process that that is affected in subjects experiencing SI as it is hypothesized that neuroplasticity may be a key event in many psychiatric disorders [52]. This is consistent with our findings were the molecular pathways associated with neuronal migration and proliferation were associated with SI.

The present investigation is characterized by several limits. The CATIE study was not designed for the analysis of the phenotype under investigation, and it is underpowered to retrieve a GWAS significant result. In order to define the phenotype under analysis, an item of the Calgary scale was used and not a dedicated psychologic test, limiting the clinical information that can be retrieved by our analysis. No imputation was conducted due to the poor quality of the original database not granting valuable imputed data. The molecular pathway analysis is prone to false positive findings, despite the Bonferroni and FDR corrections. Moreover, it depends on the current annotation system, which is changing year after year. Table 6 (supplementary data) reports the genes that were found to be enriched in SNPs associated with the phenotype under analysis.

In the table, the gene ratio indicates the k/n value and the Bg ratio indicates the M/N value, where N is the total number of genes with annotation, M is the number of genes annotated in that distribution, n is the number of genes included in the analysis, and k is the number of genes annotated to the node. P adjust indicates the P level after Bonferroni correction for multitesting. q Value indicates the P level after false discovery rate correction for multitesting.

Confusion Matrix for the Ranfom Forest Analysis. OOB estimate of error rate was 0.88%. 20 trees with the analysis of up to 5 variables per node were used.

The molecular pathways containing significantly more SNPs associated with the phenotype under analysis than expected by chance are reported. Bar length represents the number of genes enriched in each pathway. The shadowing of the bars represents the P level after correction for multiple tests.

All the participants gave written, informed consent prior to entering the study, and the protocol was conducted in full compliance with the Declaration of Helsinki.

## Supplementary Information

Below is the link to the electronic supplementary material.

Supplementary file1 (DOCX 74 KB)
