# Supplementary material for: Genetics of suicide ideation. A role for inflammation and neuroplasticity?
Source: Eur Arch Psychiatry Clin Neurosci. 2024 Jun 15;274(7):1527–41. doi: 10.1007/s00406-024-01836-6 (PMC11422468; doi:10.1007/s00406-024-01836-6)
Supplement: Supplementary file 1 — Supplementary file1 (DOCX 74 KB) [file 406_2024_1836_MOESM1_ESM.docx]

Table 6. Genes description

| **external_gene_name** | **chr** | **start_position** | **end_position** | **phenotype_description** | **wikigene_description** |
| --- | --- | --- | --- | --- | --- |
| DLGAP3 | 1 | 34865436 | 34929650 |  | DLG associated protein 3 |
| PTPRF | 1 | 43525187 | 43623666 | Isolated congenital breast hypoplasia/aplasia | protein tyrosine phosphatase receptor type F |
| PTPRF | 1 | 43525187 | 43623666 | BREASTS AND/OR NIPPLES APLASIA OR HYPOPLASIA OF 2 | protein tyrosine phosphatase receptor type F |
| PTPRF | 1 | 43525187 | 43623666 | ATHELIA | protein tyrosine phosphatase receptor type F |
| LAMC2 | 1 | 183186238 | 183245127 | Intermediate generalized junctional epidermolysis bullosa | laminin subunit gamma 2 |
| LAMC2 | 1 | 183186238 | 183245127 | Severe generalized junctional epidermolysis bullosa | laminin subunit gamma 2 |
| LAMC2 | 1 | 183186238 | 183245127 | EPIDERMOLYSIS BULLOSA JUNCTIONAL 1B SEVERE | laminin subunit gamma 2 |
| LAMC2 | 1 | 183186238 | 183245127 | EPIDERMOLYSIS BULLOSA JUNCTIONAL 3A INTERMEDIATE | laminin subunit gamma 2 |
| LAMC2 | 1 | 183186238 | 183245127 | EPIDERMOLYSIS BULLOSA JUNCTIONAL 3B SEVERE | laminin subunit gamma 2 |
| LAMC2 | 1 | 183186238 | 183245127 | Epidermolysis Bullosa Junctional lethal Herlitz | laminin subunit gamma 2 |
| GALNT2 | 1 | 230057990 | 230282122 | CONGENITAL DISORDER OF GLYCOSYLATION TYPE IIt | polypeptide N-acetylgalactosaminyltransferase 2 |
| ST3GAL3 | 1 | 43705824 | 43931165 | Autosomal recessive non-syndromic intellectual disability | ST3 beta-galactoside alpha-2,3-sialyltransferase 3 |
| ST3GAL3 | 1 | 43705824 | 43931165 | Infantile spasms syndrome | ST3 beta-galactoside alpha-2,3-sialyltransferase 3 |
| ST3GAL3 | 1 | 43705824 | 43931165 | DEVELOPMENTAL AND EPILEPTIC ENCEPHALOPATHY 15 | ST3 beta-galactoside alpha-2,3-sialyltransferase 3 |
| ST3GAL3 | 1 | 43705824 | 43931165 | INTELLECTUAL DEVELOPMENTAL DISORDER AUTOSOMAL RECESSIVE 12 | ST3 beta-galactoside alpha-2,3-sialyltransferase 3 |
| HSPG2 | 1 | 21822244 | 21937310 | 1p36 deletion syndrome | heparan sulfate proteoglycan 2 |
| HSPG2 | 1 | 21822244 | 21937310 | Dyssegmental dysplasia Silverman-Handmaker type | heparan sulfate proteoglycan 2 |
| HSPG2 | 1 | 21822244 | 21937310 | Schwartz-Jampel syndrome | heparan sulfate proteoglycan 2 |
| HSPG2 | 1 | 21822244 | 21937310 | DYSSEGMENTAL DYSPLASIA SILVERMAN-HANDMAKER TYPE | heparan sulfate proteoglycan 2 |
| HSPG2 | 1 | 21822244 | 21937310 | Schwartz-Jampel syndrome type 1 | heparan sulfate proteoglycan 2 |
| DDR2 | 1 | 162631373 | 162787405 | Spondyloepimetaphyseal dysplasia-short limb-abnormal calcification syndrome | discoidin domain receptor tyrosine kinase 2 |
| DDR2 | 1 | 162631373 | 162787405 | SPONDYLOMETAEPIPHYSEAL DYSPLASIA SHORT LIMB-HAND TYPE | discoidin domain receptor tyrosine kinase 2 |
| DDR2 | 1 | 162631373 | 162787405 | Warburg-Cinotti syndrome | discoidin domain receptor tyrosine kinase 2 |
| DDR2 | 1 | 162631373 | 162787405 | SPONDYLOEPIMETAPHYSEAL DYSPLASIA SHORT LIMB-HAND TYPE | discoidin domain receptor tyrosine kinase 2 |
| DDR2 | 1 | 162631373 | 162787405 | Atypical Meningioma | discoidin domain receptor tyrosine kinase 2 |
| DDR2 | 1 | 162631373 | 162787405 | Bladder Small Cell Neuroendocrine Carcinoma | discoidin domain receptor tyrosine kinase 2 |
| DDR2 | 1 | 162631373 | 162787405 | Brain Stem Glioblastoma | discoidin domain receptor tyrosine kinase 2 |
| DDR2 | 1 | 162631373 | 162787405 | Breast Carcinoma by Gene Expression Profile | discoidin domain receptor tyrosine kinase 2 |
| DDR2 | 1 | 162631373 | 162787405 | Burkitts lymphoma | discoidin domain receptor tyrosine kinase 2 |
| DDR2 | 1 | 162631373 | 162787405 | Chordoid Meningioma | discoidin domain receptor tyrosine kinase 2 |
| DDR2 | 1 | 162631373 | 162787405 | Endometrial Endometrioid Adenocarcinoma | discoidin domain receptor tyrosine kinase 2 |
| DDR2 | 1 | 162631373 | 162787405 | Gallbladder Small Cell Neuroendocrine Carcinoma | discoidin domain receptor tyrosine kinase 2 |
| DDR2 | 1 | 162631373 | 162787405 | Hepatobiliary Neoplasm | discoidin domain receptor tyrosine kinase 2 |
| DDR2 | 1 | 162631373 | 162787405 | Hodgkins lymphoma | discoidin domain receptor tyrosine kinase 2 |
| DDR2 | 1 | 162631373 | 162787405 | Invasive Breast Carcinoma | discoidin domain receptor tyrosine kinase 2 |
| DDR2 | 1 | 162631373 | 162787405 | MANTLE CELL LYMPHOMA | discoidin domain receptor tyrosine kinase 2 |
| DDR2 | 1 | 162631373 | 162787405 | Merkel cell skin cancer | discoidin domain receptor tyrosine kinase 2 |
| DDR2 | 1 | 162631373 | 162787405 | ND | discoidin domain receptor tyrosine kinase 2 |
| DDR2 | 1 | 162631373 | 162787405 | Ovarian Endometrioid Adenocarcinoma with Squamous Differentiation | discoidin domain receptor tyrosine kinase 2 |
| DDR2 | 1 | 162631373 | 162787405 | Placental Choriocarcinoma | discoidin domain receptor tyrosine kinase 2 |
| DDR2 | 1 | 162631373 | 162787405 | Secretory Meningioma | discoidin domain receptor tyrosine kinase 2 |
| DDR2 | 1 | 162631373 | 162787405 | Testicular Teratoma | discoidin domain receptor tyrosine kinase 2 |
| DDR2 | 1 | 162631373 | 162787405 | Thymic Squamous Cell Carcinoma | discoidin domain receptor tyrosine kinase 2 |
| DDR2 | 1 | 162631373 | 162787405 | Thyroid Gland Undifferentiated (Anaplastic) Carcinoma | discoidin domain receptor tyrosine kinase 2 |
| DDR2 | 1 | 162631373 | 162787405 | Transitional Meningioma | discoidin domain receptor tyrosine kinase 2 |
| DDR2 | 1 | 162631373 | 162787405 | Acute lymphoblastic leukemia | discoidin domain receptor tyrosine kinase 2 |
| DDR2 | 1 | 162631373 | 162787405 | Acute myeloid leukemia | discoidin domain receptor tyrosine kinase 2 |
| DDR2 | 1 | 162631373 | 162787405 | adenosquamous lung carcinoma | discoidin domain receptor tyrosine kinase 2 |
| DDR2 | 1 | 162631373 | 162787405 | Anaplastic astrocytoma | discoidin domain receptor tyrosine kinase 2 |
| DDR2 | 1 | 162631373 | 162787405 | bile duct carcinoma | discoidin domain receptor tyrosine kinase 2 |
| DDR2 | 1 | 162631373 | 162787405 | bladder transitional cell carcinoma | discoidin domain receptor tyrosine kinase 2 |
| DDR2 | 1 | 162631373 | 162787405 | brain glioblastoma | discoidin domain receptor tyrosine kinase 2 |
| DDR2 | 1 | 162631373 | 162787405 | Breast carcinoma | discoidin domain receptor tyrosine kinase 2 |
| DDR2 | 1 | 162631373 | 162787405 | breast ductal adenocarcinoma | discoidin domain receptor tyrosine kinase 2 |
| DDR2 | 1 | 162631373 | 162787405 | cecum adenocarcinoma | discoidin domain receptor tyrosine kinase 2 |
| DDR2 | 1 | 162631373 | 162787405 | cervical carcinoma | discoidin domain receptor tyrosine kinase 2 |
| DDR2 | 1 | 162631373 | 162787405 | cervical squamous cell carcinoma | discoidin domain receptor tyrosine kinase 2 |
| DDR2 | 1 | 162631373 | 162787405 | Chronic lymphocytic leukemia | discoidin domain receptor tyrosine kinase 2 |
| DDR2 | 1 | 162631373 | 162787405 | Clear cell renal carcinoma | discoidin domain receptor tyrosine kinase 2 |
| DDR2 | 1 | 162631373 | 162787405 | colon adenocarcinoma | discoidin domain receptor tyrosine kinase 2 |
| DDR2 | 1 | 162631373 | 162787405 | colon carcinoma | discoidin domain receptor tyrosine kinase 2 |
| DDR2 | 1 | 162631373 | 162787405 | colorectal adenocarcinoma | discoidin domain receptor tyrosine kinase 2 |
| DDR2 | 1 | 162631373 | 162787405 | diffuse gastric adenocarcinoma | discoidin domain receptor tyrosine kinase 2 |
| DDR2 | 1 | 162631373 | 162787405 | diffuse large B-cell lymphoma | discoidin domain receptor tyrosine kinase 2 |
| DDR2 | 1 | 162631373 | 162787405 | endometrium adenocarcinoma | discoidin domain receptor tyrosine kinase 2 |
| DDR2 | 1 | 162631373 | 162787405 | Ependymoma | discoidin domain receptor tyrosine kinase 2 |
| DDR2 | 1 | 162631373 | 162787405 | ganglioneuroblastoma | discoidin domain receptor tyrosine kinase 2 |
| DDR2 | 1 | 162631373 | 162787405 | gastric adenocarcinoma | discoidin domain receptor tyrosine kinase 2 |
| DDR2 | 1 | 162631373 | 162787405 | head and neck squamous cell carcinoma | discoidin domain receptor tyrosine kinase 2 |
| DDR2 | 1 | 162631373 | 162787405 | Hepatocellular Carcinoma | discoidin domain receptor tyrosine kinase 2 |
| DDR2 | 1 | 162631373 | 162787405 | kidney neoplasm | discoidin domain receptor tyrosine kinase 2 |
| DDR2 | 1 | 162631373 | 162787405 | large cell lung carcinoma | discoidin domain receptor tyrosine kinase 2 |
| DDR2 | 1 | 162631373 | 162787405 | large cell medulloblastoma | discoidin domain receptor tyrosine kinase 2 |
| DDR2 | 1 | 162631373 | 162787405 | lobular breast carcinoma | discoidin domain receptor tyrosine kinase 2 |
| DDR2 | 1 | 162631373 | 162787405 | Lung adenocarcinoma | discoidin domain receptor tyrosine kinase 2 |
| DDR2 | 1 | 162631373 | 162787405 | Lung carcinoma | discoidin domain receptor tyrosine kinase 2 |
| DDR2 | 1 | 162631373 | 162787405 | lymphoid neoplasm | discoidin domain receptor tyrosine kinase 2 |
| DDR2 | 1 | 162631373 | 162787405 | Melanoma | discoidin domain receptor tyrosine kinase 2 |
| DDR2 | 1 | 162631373 | 162787405 | Multiple myeloma | discoidin domain receptor tyrosine kinase 2 |
| DDR2 | 1 | 162631373 | 162787405 | nodular melanoma | discoidin domain receptor tyrosine kinase 2 |
| DDR2 | 1 | 162631373 | 162787405 | non-small cell lung carcinoma | discoidin domain receptor tyrosine kinase 2 |
| DDR2 | 1 | 162631373 | 162787405 | oral squamous cell carcinoma | discoidin domain receptor tyrosine kinase 2 |
| DDR2 | 1 | 162631373 | 162787405 | ovarian clear cell adenocarcinoma | discoidin domain receptor tyrosine kinase 2 |
| DDR2 | 1 | 162631373 | 162787405 | ovarian mucinous adenocarcinoma | discoidin domain receptor tyrosine kinase 2 |
| DDR2 | 1 | 162631373 | 162787405 | ovarian teratoma | discoidin domain receptor tyrosine kinase 2 |
| DDR2 | 1 | 162631373 | 162787405 | pancreatic carcinoma | discoidin domain receptor tyrosine kinase 2 |
| DDR2 | 1 | 162631373 | 162787405 | pancreatic ductal adenocarcinoma | discoidin domain receptor tyrosine kinase 2 |
| DDR2 | 1 | 162631373 | 162787405 | pancreatic neuroendocrine tumor | discoidin domain receptor tyrosine kinase 2 |
| DDR2 | 1 | 162631373 | 162787405 | pharyngeal squamous cell carcinoma | discoidin domain receptor tyrosine kinase 2 |
| DDR2 | 1 | 162631373 | 162787405 | prostate adenocarcinoma | discoidin domain receptor tyrosine kinase 2 |
| DDR2 | 1 | 162631373 | 162787405 | prostate carcinoma | discoidin domain receptor tyrosine kinase 2 |
| DDR2 | 1 | 162631373 | 162787405 | rectal adenocarcinoma | discoidin domain receptor tyrosine kinase 2 |
| DDR2 | 1 | 162631373 | 162787405 | Renal cell carcinoma | discoidin domain receptor tyrosine kinase 2 |
| DDR2 | 1 | 162631373 | 162787405 | salivary gland squamous cell carcinoma | discoidin domain receptor tyrosine kinase 2 |
| DDR2 | 1 | 162631373 | 162787405 | skin carcinoma | discoidin domain receptor tyrosine kinase 2 |
| DDR2 | 1 | 162631373 | 162787405 | Small cell lung carcinoma | discoidin domain receptor tyrosine kinase 2 |
| DDR2 | 1 | 162631373 | 162787405 | squamous cell lung carcinoma | discoidin domain receptor tyrosine kinase 2 |
| DDR2 | 1 | 162631373 | 162787405 | testicular mixed germ cell tumor | discoidin domain receptor tyrosine kinase 2 |
| DDR2 | 1 | 162631373 | 162787405 | thyroid carcinoma | discoidin domain receptor tyrosine kinase 2 |
| DDR2 | 1 | 162631373 | 162787405 | undifferentiated pleomorphic sarcoma | discoidin domain receptor tyrosine kinase 2 |
| DDR2 | 1 | 162631373 | 162787405 | urothelial carcinoma | discoidin domain receptor tyrosine kinase 2 |
| KCNH1 | 1 | 210676823 | 211134165 | TEMPLE-BARAITSER SYNDROME | potassium voltage-gated channel subfamily H member 1 |
| KCNH1 | 1 | 210676823 | 211134165 | Zimmermann-Laband syndrome | potassium voltage-gated channel subfamily H member 1 |
| KCNH1 | 1 | 210676823 | 211134165 | ZIMMERMANN-LABAND SYNDROME 1 | potassium voltage-gated channel subfamily H member 1 |
| KCNH1 | 1 | 210676823 | 211134165 | Temple barraister syndrome | potassium voltage-gated channel subfamily H member 1 |
| SYT2 | 1 | 202590596 | 202710454 | Presynaptic congenital myasthenic syndromes | synaptotagmin 2 |
| SYT2 | 1 | 202590596 | 202710454 | MYASTHENIC SYNDROME CONGENITAL 7A PRESYNAPTIC AND DISTAL MOTOR NEUROPATHY AUTOSOMAL DOMINANT | synaptotagmin 2 |
| SYT2 | 1 | 202590596 | 202710454 | MYASTHENIC SYNDROME CONGENITAL 7B PRESYNAPTIC AUTOSOMAL RECESSIVE | synaptotagmin 2 |
| SYT2 | 1 | 202590596 | 202710454 | SYT2-related congenital onset presynaptic myasthenic syndrome | synaptotagmin 2 |
| CACNA1E | 1 | 181317690 | 181813262 | DEVELOPMENTAL AND EPILEPTIC ENCEPHALOPATHY 69 | calcium voltage-gated channel subunit alpha1 E |
| CACNA1E | 1 | 181317690 | 181813262 | Epileptic Encephalopathy with Contractures Macrocephaly and Dyskinesia | calcium voltage-gated channel subunit alpha1 E |
| ST6GALNAC3 | 1 | 76074746 | 76634603 |  | ST6 N-acetylgalactosaminide alpha-2,6-sialyltransferase 3 |
| GALNT14 | 2 | 30910467 | 31155202 |  | polypeptide N-acetylgalactosaminyltransferase 14 |
| GALNT13 | 2 | 153871922 | 154453979 |  | polypeptide N-acetylgalactosaminyltransferase 13 |
| UGT1A9 | 2 | 233671898 | 233773300 |  | UDP glucuronosyltransferase family 1 member A9 |
| UGT1A6 | 2 | 233691607 | 233773300 |  | UDP glucuronosyltransferase family 1 member A6 |
| UGT1A4 | 2 | 233718736 | 233773300 |  | UDP glucuronosyltransferase family 1 member A4 |
| LRRTM4 | 2 | 76747685 | 77593319 |  | leucine rich repeat transmembrane neuronal 4 |
| COL4A3 | 2 | 227164624 | 227314792 | Autosomal dominant Alport syndrome | collagen type IV alpha 3 chain |
| COL4A3 | 2 | 227164624 | 227314792 | Autosomal recessive Alport syndrome | collagen type IV alpha 3 chain |
| COL4A3 | 2 | 227164624 | 227314792 | Genetic steroid-resistant nephrotic syndrome | collagen type IV alpha 3 chain |
| COL4A3 | 2 | 227164624 | 227314792 | NON RARE IN EUROPE: Benign familial hematuria | collagen type IV alpha 3 chain |
| COL4A3 | 2 | 227164624 | 227314792 | ALPORT SYNDROME 2 AUTOSOMAL RECESSIVE | collagen type IV alpha 3 chain |
| COL4A3 | 2 | 227164624 | 227314792 | Alport syndrome 3 autosomal dominant | collagen type IV alpha 3 chain |
| COL4A3 | 2 | 227164624 | 227314792 | HEMATURIA BENIGN FAMILIAL | collagen type IV alpha 3 chain |
| COL4A3 | 2 | 227164624 | 227314792 | Alport syndrome autosomal dominant | collagen type IV alpha 3 chain |
| COL4A3 | 2 | 227164624 | 227314792 | ALPORT SYNDROME AUTOSOMAL RECESSIVE | collagen type IV alpha 3 chain |
| COL6A3 | 2 | 237324003 | 237414328 | BETHLEM MYOPATHY | collagen type VI alpha 3 chain |
| COL6A3 | 2 | 237324003 | 237414328 | Congenital muscular dystrophy Ullrich type | collagen type VI alpha 3 chain |
| COL6A3 | 2 | 237324003 | 237414328 | Primary dystonia DYT27 type | collagen type VI alpha 3 chain |
| COL6A3 | 2 | 237324003 | 237414328 | Bethlem myopathy 1 | collagen type VI alpha 3 chain |
| COL6A3 | 2 | 237324003 | 237414328 | DYSTONIA 27 | collagen type VI alpha 3 chain |
| COL6A3 | 2 | 237324003 | 237414328 | ULLRICH CONGENITAL MUSCULAR DYSTROPHY 1 | collagen type VI alpha 3 chain |
| UGT1A3 | 2 | 233729042 | 233773300 |  | UDP glucuronosyltransferase family 1 member A3 |
| UGT1A8 | 2 | 233617633 | 233773300 |  | UDP glucuronosyltransferase family 1 member A8 |
| UGT1A5 | 2 | 233712907 | 233773300 |  | UDP glucuronosyltransferase family 1 member A5 |
| UGT1A7 | 2 | 233681901 | 233773300 |  | UDP glucuronosyltransferase family 1 member A7 |
| THSD7B | 2 | 136765545 | 137677718 |  | thrombospondin type 1 domain containing 7B |
| NRXN1 | 2 | 49918503 | 51225575 | NON RARE IN EUROPE | neurexin 1 |
| NRXN1 | 2 | 49918503 | 51225575 | NRXN1-related severe neurodevelopmental disorder-motor stereotypies-chronic constipation-sleep-wake cycle disturbance | neurexin 1 |
| NRXN1 | 2 | 49918503 | 51225575 | CHROMOSOME 2p16.3 DELETION SYNDROMESCHIZOPHRENIA 17 INCLUDED | neurexin 1 |
| NRXN1 | 2 | 49918503 | 51225575 | Pitt-Hopkins-like syndrome 2 | neurexin 1 |
| NRXN1 | 2 | 49918503 | 51225575 | Autism | neurexin 1 |
| NRXN1 | 2 | 49918503 | 51225575 | Pitt Hopkins 2 | neurexin 1 |
| UGT1A10 | 2 | 233636448 | 233773300 |  | UDP glucuronosyltransferase family 1 member A10 |
| PDE11A | 2 | 177623244 | 178108339 | Carney complex | phosphodiesterase 11A |
| PDE11A | 2 | 177623244 | 178108339 | Primary pigmented nodular adrenocortical disease | phosphodiesterase 11A |
| PDE11A | 2 | 177623244 | 178108339 | PIGMENTED NODULAR ADRENOCORTICAL DISEASE PRIMARY 2 | phosphodiesterase 11A |
| CREB1 | 2 | 207529737 | 207605988 | Melanoma of soft tissue | cAMP responsive element binding protein 1 |
| CREB1 | 2 | 207529737 | 207605988 | HISTIOCYTOMA ANGIOMATOID FIBROUS | cAMP responsive element binding protein 1 |
| CREB1 | 2 | 207529737 | 207605988 | Atypical Meningioma | cAMP responsive element binding protein 1 |
| CREB1 | 2 | 207529737 | 207605988 | Brain Stem Glioblastoma | cAMP responsive element binding protein 1 |
| CREB1 | 2 | 207529737 | 207605988 | Burkitts lymphoma | cAMP responsive element binding protein 1 |
| CREB1 | 2 | 207529737 | 207605988 | Cortisol-Producing Adrenal Cortex Adenoma | cAMP responsive element binding protein 1 |
| CREB1 | 2 | 207529737 | 207605988 | Endometrial Endometrioid Adenocarcinoma | cAMP responsive element binding protein 1 |
| CREB1 | 2 | 207529737 | 207605988 | Hepatobiliary Neoplasm | cAMP responsive element binding protein 1 |
| CREB1 | 2 | 207529737 | 207605988 | Merkel cell skin cancer | cAMP responsive element binding protein 1 |
| CREB1 | 2 | 207529737 | 207605988 | ND | cAMP responsive element binding protein 1 |
| CREB1 | 2 | 207529737 | 207605988 | Ovarian Endometrioid Adenocarcinoma with Squamous Differentiation | cAMP responsive element binding protein 1 |
| CREB1 | 2 | 207529737 | 207605988 | Placental Choriocarcinoma | cAMP responsive element binding protein 1 |
| CREB1 | 2 | 207529737 | 207605988 | T-cell acute lymphoblastic leukemia | cAMP responsive element binding protein 1 |
| CREB1 | 2 | 207529737 | 207605988 | adenosquamous lung carcinoma | cAMP responsive element binding protein 1 |
| CREB1 | 2 | 207529737 | 207605988 | Anaplastic astrocytoma | cAMP responsive element binding protein 1 |
| CREB1 | 2 | 207529737 | 207605988 | bile duct carcinoma | cAMP responsive element binding protein 1 |
| CREB1 | 2 | 207529737 | 207605988 | bladder transitional cell carcinoma | cAMP responsive element binding protein 1 |
| CREB1 | 2 | 207529737 | 207605988 | brain glioblastoma | cAMP responsive element binding protein 1 |
| CREB1 | 2 | 207529737 | 207605988 | Breast carcinoma | cAMP responsive element binding protein 1 |
| CREB1 | 2 | 207529737 | 207605988 | breast ductal adenocarcinoma | cAMP responsive element binding protein 1 |
| CREB1 | 2 | 207529737 | 207605988 | cecum adenocarcinoma | cAMP responsive element binding protein 1 |
| CREB1 | 2 | 207529737 | 207605988 | cervical squamous cell carcinoma | cAMP responsive element binding protein 1 |
| CREB1 | 2 | 207529737 | 207605988 | Chronic lymphocytic leukemia | cAMP responsive element binding protein 1 |
| CREB1 | 2 | 207529737 | 207605988 | Clear cell renal carcinoma | cAMP responsive element binding protein 1 |
| CREB1 | 2 | 207529737 | 207605988 | clear cell sarcoma | cAMP responsive element binding protein 1 |
| CREB1 | 2 | 207529737 | 207605988 | colon adenocarcinoma | cAMP responsive element binding protein 1 |
| CREB1 | 2 | 207529737 | 207605988 | colorectal adenocarcinoma | cAMP responsive element binding protein 1 |
| CREB1 | 2 | 207529737 | 207605988 | cutaneous fibrous histiocytoma | cAMP responsive element binding protein 1 |
| CREB1 | 2 | 207529737 | 207605988 | diffuse large B-cell lymphoma | cAMP responsive element binding protein 1 |
| CREB1 | 2 | 207529737 | 207605988 | gastric adenocarcinoma | cAMP responsive element binding protein 1 |
| CREB1 | 2 | 207529737 | 207605988 | Hepatocellular Carcinoma | cAMP responsive element binding protein 1 |
| CREB1 | 2 | 207529737 | 207605988 | kidney neoplasm | cAMP responsive element binding protein 1 |
| CREB1 | 2 | 207529737 | 207605988 | lobular breast carcinoma | cAMP responsive element binding protein 1 |
| CREB1 | 2 | 207529737 | 207605988 | Lung adenocarcinoma | cAMP responsive element binding protein 1 |
| CREB1 | 2 | 207529737 | 207605988 | Lung carcinoma | cAMP responsive element binding protein 1 |
| CREB1 | 2 | 207529737 | 207605988 | lymphoid neoplasm | cAMP responsive element binding protein 1 |
| CREB1 | 2 | 207529737 | 207605988 | Neoplasm | cAMP responsive element binding protein 1 |
| CREB1 | 2 | 207529737 | 207605988 | nodular melanoma | cAMP responsive element binding protein 1 |
| CREB1 | 2 | 207529737 | 207605988 | oral squamous cell carcinoma | cAMP responsive element binding protein 1 |
| CREB1 | 2 | 207529737 | 207605988 | pancreatic carcinoma | cAMP responsive element binding protein 1 |
| CREB1 | 2 | 207529737 | 207605988 | pancreatic ductal adenocarcinoma | cAMP responsive element binding protein 1 |
| CREB1 | 2 | 207529737 | 207605988 | pancreatic neuroendocrine tumor | cAMP responsive element binding protein 1 |
| CREB1 | 2 | 207529737 | 207605988 | pharyngeal squamous cell carcinoma | cAMP responsive element binding protein 1 |
| CREB1 | 2 | 207529737 | 207605988 | prostate adenocarcinoma | cAMP responsive element binding protein 1 |
| CREB1 | 2 | 207529737 | 207605988 | prostate carcinoma | cAMP responsive element binding protein 1 |
| CREB1 | 2 | 207529737 | 207605988 | rectal adenocarcinoma | cAMP responsive element binding protein 1 |
| CREB1 | 2 | 207529737 | 207605988 | skin carcinoma | cAMP responsive element binding protein 1 |
| CREB1 | 2 | 207529737 | 207605988 | Small cell lung carcinoma | cAMP responsive element binding protein 1 |
| CREB1 | 2 | 207529737 | 207605988 | soft tissue sarcoma | cAMP responsive element binding protein 1 |
| CREB1 | 2 | 207529737 | 207605988 | squamous cell lung carcinoma | cAMP responsive element binding protein 1 |
| PDE1A | 2 | 182139968 | 182523359 |  | phosphodiesterase 1A |
| ERBB4 | 2 | 211375717 | 212538841 | Amyotrophic lateral sclerosis | erb-b2 receptor tyrosine kinase 4 |
| ERBB4 | 2 | 211375717 | 212538841 | Autosomal dominant non-syndromic intellectual disability | erb-b2 receptor tyrosine kinase 4 |
| ERBB4 | 2 | 211375717 | 212538841 | Amyotrophic lateral sclerosis 19 | erb-b2 receptor tyrosine kinase 4 |
| ERBB4 | 2 | 211375717 | 212538841 | Appendix Adenocarcinoma | erb-b2 receptor tyrosine kinase 4 |
| ERBB4 | 2 | 211375717 | 212538841 | Atypical Meningioma | erb-b2 receptor tyrosine kinase 4 |
| ERBB4 | 2 | 211375717 | 212538841 | B-cell acute lymphoblastic leukemia | erb-b2 receptor tyrosine kinase 4 |
| ERBB4 | 2 | 211375717 | 212538841 | Bladder Adenocarcinoma | erb-b2 receptor tyrosine kinase 4 |
| ERBB4 | 2 | 211375717 | 212538841 | Brain Stem Glioblastoma | erb-b2 receptor tyrosine kinase 4 |
| ERBB4 | 2 | 211375717 | 212538841 | Breast Carcinoma by Gene Expression Profile | erb-b2 receptor tyrosine kinase 4 |
| ERBB4 | 2 | 211375717 | 212538841 | Burkitts lymphoma | erb-b2 receptor tyrosine kinase 4 |
| ERBB4 | 2 | 211375717 | 212538841 | Choroid plexus papilloma | erb-b2 receptor tyrosine kinase 4 |
| ERBB4 | 2 | 211375717 | 212538841 | Digestive System Carcinoma | erb-b2 receptor tyrosine kinase 4 |
| ERBB4 | 2 | 211375717 | 212538841 | Duodenal Adenocarcinoma | erb-b2 receptor tyrosine kinase 4 |
| ERBB4 | 2 | 211375717 | 212538841 | Endometrial Endometrioid Adenocarcinoma | erb-b2 receptor tyrosine kinase 4 |
| ERBB4 | 2 | 211375717 | 212538841 | Hepatobiliary Neoplasm | erb-b2 receptor tyrosine kinase 4 |
| ERBB4 | 2 | 211375717 | 212538841 | Hodgkins lymphoma | erb-b2 receptor tyrosine kinase 4 |
| ERBB4 | 2 | 211375717 | 212538841 | MANTLE CELL LYMPHOMA | erb-b2 receptor tyrosine kinase 4 |
| ERBB4 | 2 | 211375717 | 212538841 | Merkel cell skin cancer | erb-b2 receptor tyrosine kinase 4 |
| ERBB4 | 2 | 211375717 | 212538841 | ND | erb-b2 receptor tyrosine kinase 4 |
| ERBB4 | 2 | 211375717 | 212538841 | Ovarian Endometrioid Adenocarcinoma | erb-b2 receptor tyrosine kinase 4 |
| ERBB4 | 2 | 211375717 | 212538841 | Ovarian Endometrioid Adenocarcinoma with Squamous Differentiation | erb-b2 receptor tyrosine kinase 4 |
| ERBB4 | 2 | 211375717 | 212538841 | Pancreatic Acinar Cell Carcinoma | erb-b2 receptor tyrosine kinase 4 |
| ERBB4 | 2 | 211375717 | 212538841 | Parotid Gland Squamous Cell Carcinoma | erb-b2 receptor tyrosine kinase 4 |
| ERBB4 | 2 | 211375717 | 212538841 | Placental Choriocarcinoma | erb-b2 receptor tyrosine kinase 4 |
| ERBB4 | 2 | 211375717 | 212538841 | Pleural Biphasic Mesothelioma | erb-b2 receptor tyrosine kinase 4 |
| ERBB4 | 2 | 211375717 | 212538841 | Poroma | erb-b2 receptor tyrosine kinase 4 |
| ERBB4 | 2 | 211375717 | 212538841 | Sinonasal Undifferentiated Carcinoma | erb-b2 receptor tyrosine kinase 4 |
| ERBB4 | 2 | 211375717 | 212538841 | T-cell acute lymphoblastic leukemia | erb-b2 receptor tyrosine kinase 4 |
| ERBB4 | 2 | 211375717 | 212538841 | Testicular Choriocarcinoma | erb-b2 receptor tyrosine kinase 4 |
| ERBB4 | 2 | 211375717 | 212538841 | Thymic Carcinoma | erb-b2 receptor tyrosine kinase 4 |
| ERBB4 | 2 | 211375717 | 212538841 | Thyroid Gland Undifferentiated (Anaplastic) Carcinoma | erb-b2 receptor tyrosine kinase 4 |
| ERBB4 | 2 | 211375717 | 212538841 | Unclassified Renal Cell Carcinoma | erb-b2 receptor tyrosine kinase 4 |
| ERBB4 | 2 | 211375717 | 212538841 | Undifferentiated Pancreatic Carcinoma | erb-b2 receptor tyrosine kinase 4 |
| ERBB4 | 2 | 211375717 | 212538841 | Vulvar Squamous Cell Carcinoma | erb-b2 receptor tyrosine kinase 4 |
| ERBB4 | 2 | 211375717 | 212538841 | Acute lymphoblastic leukemia | erb-b2 receptor tyrosine kinase 4 |
| ERBB4 | 2 | 211375717 | 212538841 | Acute myeloid leukemia | erb-b2 receptor tyrosine kinase 4 |
| ERBB4 | 2 | 211375717 | 212538841 | Adenoid cystic carcinoma | erb-b2 receptor tyrosine kinase 4 |
| ERBB4 | 2 | 211375717 | 212538841 | adrenocortical adenoma | erb-b2 receptor tyrosine kinase 4 |
| ERBB4 | 2 | 211375717 | 212538841 | Anaplastic astrocytoma | erb-b2 receptor tyrosine kinase 4 |
| ERBB4 | 2 | 211375717 | 212538841 | angiosarcoma | erb-b2 receptor tyrosine kinase 4 |
| ERBB4 | 2 | 211375717 | 212538841 | Astrocytoma | erb-b2 receptor tyrosine kinase 4 |
| ERBB4 | 2 | 211375717 | 212538841 | bile duct carcinoma | erb-b2 receptor tyrosine kinase 4 |
| ERBB4 | 2 | 211375717 | 212538841 | bladder transitional cell carcinoma | erb-b2 receptor tyrosine kinase 4 |
| ERBB4 | 2 | 211375717 | 212538841 | brain glioblastoma | erb-b2 receptor tyrosine kinase 4 |
| ERBB4 | 2 | 211375717 | 212538841 | Breast carcinoma | erb-b2 receptor tyrosine kinase 4 |
| ERBB4 | 2 | 211375717 | 212538841 | breast ductal adenocarcinoma | erb-b2 receptor tyrosine kinase 4 |
| ERBB4 | 2 | 211375717 | 212538841 | cecum adenocarcinoma | erb-b2 receptor tyrosine kinase 4 |
| ERBB4 | 2 | 211375717 | 212538841 | cervical carcinoma | erb-b2 receptor tyrosine kinase 4 |
| ERBB4 | 2 | 211375717 | 212538841 | cervical squamous cell carcinoma | erb-b2 receptor tyrosine kinase 4 |
| ERBB4 | 2 | 211375717 | 212538841 | Chronic lymphocytic leukemia | erb-b2 receptor tyrosine kinase 4 |
| ERBB4 | 2 | 211375717 | 212538841 | Chronic myelomonocytic leukemia | erb-b2 receptor tyrosine kinase 4 |
| ERBB4 | 2 | 211375717 | 212538841 | Clear cell renal carcinoma | erb-b2 receptor tyrosine kinase 4 |
| ERBB4 | 2 | 211375717 | 212538841 | colon adenocarcinoma | erb-b2 receptor tyrosine kinase 4 |
| ERBB4 | 2 | 211375717 | 212538841 | colonic neoplasm | erb-b2 receptor tyrosine kinase 4 |
| ERBB4 | 2 | 211375717 | 212538841 | colorectal adenocarcinoma | erb-b2 receptor tyrosine kinase 4 |
| ERBB4 | 2 | 211375717 | 212538841 | Dedifferentiated liposarcoma | erb-b2 receptor tyrosine kinase 4 |
| ERBB4 | 2 | 211375717 | 212538841 | diffuse gastric adenocarcinoma | erb-b2 receptor tyrosine kinase 4 |
| ERBB4 | 2 | 211375717 | 212538841 | diffuse large B-cell lymphoma | erb-b2 receptor tyrosine kinase 4 |
| ERBB4 | 2 | 211375717 | 212538841 | dysplastic nevus | erb-b2 receptor tyrosine kinase 4 |
| ERBB4 | 2 | 211375717 | 212538841 | endometrium adenocarcinoma | erb-b2 receptor tyrosine kinase 4 |
| ERBB4 | 2 | 211375717 | 212538841 | Ependymoma | erb-b2 receptor tyrosine kinase 4 |
| ERBB4 | 2 | 211375717 | 212538841 | gastric adenocarcinoma | erb-b2 receptor tyrosine kinase 4 |
| ERBB4 | 2 | 211375717 | 212538841 | hairy cell leukemia | erb-b2 receptor tyrosine kinase 4 |
| ERBB4 | 2 | 211375717 | 212538841 | head and neck squamous cell carcinoma | erb-b2 receptor tyrosine kinase 4 |
| ERBB4 | 2 | 211375717 | 212538841 | Hepatocellular Carcinoma | erb-b2 receptor tyrosine kinase 4 |
| ERBB4 | 2 | 211375717 | 212538841 | kidney neoplasm | erb-b2 receptor tyrosine kinase 4 |
| ERBB4 | 2 | 211375717 | 212538841 | large cell lung carcinoma | erb-b2 receptor tyrosine kinase 4 |
| ERBB4 | 2 | 211375717 | 212538841 | large cell medulloblastoma | erb-b2 receptor tyrosine kinase 4 |
| ERBB4 | 2 | 211375717 | 212538841 | leiomyosarcoma | erb-b2 receptor tyrosine kinase 4 |
| ERBB4 | 2 | 211375717 | 212538841 | lobular breast carcinoma | erb-b2 receptor tyrosine kinase 4 |
| ERBB4 | 2 | 211375717 | 212538841 | Lung adenocarcinoma | erb-b2 receptor tyrosine kinase 4 |
| ERBB4 | 2 | 211375717 | 212538841 | Lung carcinoma | erb-b2 receptor tyrosine kinase 4 |
| ERBB4 | 2 | 211375717 | 212538841 | lymphoid neoplasm | erb-b2 receptor tyrosine kinase 4 |
| ERBB4 | 2 | 211375717 | 212538841 | mast-cell leukemia | erb-b2 receptor tyrosine kinase 4 |
| ERBB4 | 2 | 211375717 | 212538841 | Medulloblastoma | erb-b2 receptor tyrosine kinase 4 |
| ERBB4 | 2 | 211375717 | 212538841 | Melanoma | erb-b2 receptor tyrosine kinase 4 |
| ERBB4 | 2 | 211375717 | 212538841 | Neoplasm | erb-b2 receptor tyrosine kinase 4 |
| ERBB4 | 2 | 211375717 | 212538841 | neoplasm of mature B-cells | erb-b2 receptor tyrosine kinase 4 |
| ERBB4 | 2 | 211375717 | 212538841 | nodular melanoma | erb-b2 receptor tyrosine kinase 4 |
| ERBB4 | 2 | 211375717 | 212538841 | non-small cell lung carcinoma | erb-b2 receptor tyrosine kinase 4 |
| ERBB4 | 2 | 211375717 | 212538841 | oral squamous cell carcinoma | erb-b2 receptor tyrosine kinase 4 |
| ERBB4 | 2 | 211375717 | 212538841 | OSTEOSARCOMA | erb-b2 receptor tyrosine kinase 4 |
| ERBB4 | 2 | 211375717 | 212538841 | ovarian clear cell adenocarcinoma | erb-b2 receptor tyrosine kinase 4 |
| ERBB4 | 2 | 211375717 | 212538841 | ovarian mucinous adenocarcinoma | erb-b2 receptor tyrosine kinase 4 |
| ERBB4 | 2 | 211375717 | 212538841 | pancreatic carcinoma | erb-b2 receptor tyrosine kinase 4 |
| ERBB4 | 2 | 211375717 | 212538841 | pancreatic ductal adenocarcinoma | erb-b2 receptor tyrosine kinase 4 |
| ERBB4 | 2 | 211375717 | 212538841 | pancreatic neuroendocrine tumor | erb-b2 receptor tyrosine kinase 4 |
| ERBB4 | 2 | 211375717 | 212538841 | pharyngeal squamous cell carcinoma | erb-b2 receptor tyrosine kinase 4 |
| ERBB4 | 2 | 211375717 | 212538841 | prostate adenocarcinoma | erb-b2 receptor tyrosine kinase 4 |
| ERBB4 | 2 | 211375717 | 212538841 | prostate carcinoma | erb-b2 receptor tyrosine kinase 4 |
| ERBB4 | 2 | 211375717 | 212538841 | pulmonary blastoma | erb-b2 receptor tyrosine kinase 4 |
| ERBB4 | 2 | 211375717 | 212538841 | rectal adenocarcinoma | erb-b2 receptor tyrosine kinase 4 |
| ERBB4 | 2 | 211375717 | 212538841 | Renal cell carcinoma | erb-b2 receptor tyrosine kinase 4 |
| ERBB4 | 2 | 211375717 | 212538841 | salivary gland squamous cell carcinoma | erb-b2 receptor tyrosine kinase 4 |
| ERBB4 | 2 | 211375717 | 212538841 | sebaceous adenocarcinoma | erb-b2 receptor tyrosine kinase 4 |
| ERBB4 | 2 | 211375717 | 212538841 | skin carcinoma | erb-b2 receptor tyrosine kinase 4 |
| ERBB4 | 2 | 211375717 | 212538841 | small cell carcinoma | erb-b2 receptor tyrosine kinase 4 |
| ERBB4 | 2 | 211375717 | 212538841 | Small cell lung carcinoma | erb-b2 receptor tyrosine kinase 4 |
| ERBB4 | 2 | 211375717 | 212538841 | soft tissue sarcoma | erb-b2 receptor tyrosine kinase 4 |
| ERBB4 | 2 | 211375717 | 212538841 | squamous cell lung carcinoma | erb-b2 receptor tyrosine kinase 4 |
| ERBB4 | 2 | 211375717 | 212538841 | Synovial sarcoma | erb-b2 receptor tyrosine kinase 4 |
| ERBB4 | 2 | 211375717 | 212538841 | testicular seminoma | erb-b2 receptor tyrosine kinase 4 |
| ERBB4 | 2 | 211375717 | 212538841 | thyroid carcinoma | erb-b2 receptor tyrosine kinase 4 |
| ERBB4 | 2 | 211375717 | 212538841 | Ulcerative colitis | erb-b2 receptor tyrosine kinase 4 |
| ERBB4 | 2 | 211375717 | 212538841 | undifferentiated pleomorphic sarcoma | erb-b2 receptor tyrosine kinase 4 |
| ERBB4 | 2 | 211375717 | 212538841 | urothelial carcinoma | erb-b2 receptor tyrosine kinase 4 |
| COL8A1 | 3 | 99638475 | 99799226 |  | collagen type VIII alpha 1 chain |
| KCNAB1 | 3 | 156037701 | 156539138 |  | potassium voltage-gated channel subfamily A regulatory beta subunit 1 |
| ITPR1 | 3 | 4493345 | 4847506 | Aniridia-cerebellar ataxia-intellectual disability syndrome | inositol 1,4,5-trisphosphate receptor type 1 |
| ITPR1 | 3 | 4493345 | 4847506 | Spinocerebellar ataxia type 15/16 | inositol 1,4,5-trisphosphate receptor type 1 |
| ITPR1 | 3 | 4493345 | 4847506 | Spinocerebellar ataxia type 29 | inositol 1,4,5-trisphosphate receptor type 1 |
| ITPR1 | 3 | 4493345 | 4847506 | Gillespie Syndrome | inositol 1,4,5-trisphosphate receptor type 1 |
| ITPR1 | 3 | 4493345 | 4847506 | SPINOCEREBELLAR ATAXIA 15 | inositol 1,4,5-trisphosphate receptor type 1 |
| ITPR1 | 3 | 4493345 | 4847506 | SPINOCEREBELLAR ATAXIA 29 | inositol 1,4,5-trisphosphate receptor type 1 |
| ITPR1 | 3 | 4493345 | 4847506 | Gillespie Syndrome biallelic loss of function | inositol 1,4,5-trisphosphate receptor type 1 |
| ITPR1 | 3 | 4493345 | 4847506 | Gillespie Syndrome monoallelic | inositol 1,4,5-trisphosphate receptor type 1 |
| ITPR1 | 3 | 4493345 | 4847506 | Spinocerebellar ataxia 29 congenital nonprogressive | inositol 1,4,5-trisphosphate receptor type 1 |
| CACNA1D | 3 | 53328963 | 53813733 | NON RARE IN EUROPE: Aldosterone-producing adenoma | calcium voltage-gated channel subunit alpha1 D |
| CACNA1D | 3 | 53328963 | 53813733 | Primary hyperaldosteronism-seizures-neurological abnormalities syndrome | calcium voltage-gated channel subunit alpha1 D |
| CACNA1D | 3 | 53328963 | 53813733 | Sinoatrial node dysfunction and deafness | calcium voltage-gated channel subunit alpha1 D |
| CACNA1D | 3 | 53328963 | 53813733 | Primary aldosteronism seizures and neurologic abnormalities | calcium voltage-gated channel subunit alpha1 D |
| CACNA1D | 3 | 53328963 | 53813733 | Atypical Meningioma | calcium voltage-gated channel subunit alpha1 D |
| CACNA1D | 3 | 53328963 | 53813733 | B-cell acute lymphoblastic leukemia | calcium voltage-gated channel subunit alpha1 D |
| CACNA1D | 3 | 53328963 | 53813733 | Brain Stem Glioblastoma | calcium voltage-gated channel subunit alpha1 D |
| CACNA1D | 3 | 53328963 | 53813733 | Breast Carcinoma by Gene Expression Profile | calcium voltage-gated channel subunit alpha1 D |
| CACNA1D | 3 | 53328963 | 53813733 | Burkitts lymphoma | calcium voltage-gated channel subunit alpha1 D |
| CACNA1D | 3 | 53328963 | 53813733 | Endometrial Endometrioid Adenocarcinoma | calcium voltage-gated channel subunit alpha1 D |
| CACNA1D | 3 | 53328963 | 53813733 | Hepatobiliary Neoplasm | calcium voltage-gated channel subunit alpha1 D |
| CACNA1D | 3 | 53328963 | 53813733 | Hodgkins lymphoma | calcium voltage-gated channel subunit alpha1 D |
| CACNA1D | 3 | 53328963 | 53813733 | Invasive Breast Carcinoma | calcium voltage-gated channel subunit alpha1 D |
| CACNA1D | 3 | 53328963 | 53813733 | Lung Sclerosing Hemangioma | calcium voltage-gated channel subunit alpha1 D |
| CACNA1D | 3 | 53328963 | 53813733 | MANTLE CELL LYMPHOMA | calcium voltage-gated channel subunit alpha1 D |
| CACNA1D | 3 | 53328963 | 53813733 | Merkel cell skin cancer | calcium voltage-gated channel subunit alpha1 D |
| CACNA1D | 3 | 53328963 | 53813733 | ND | calcium voltage-gated channel subunit alpha1 D |
| CACNA1D | 3 | 53328963 | 53813733 | Ovarian Endometrioid Adenocarcinoma with Squamous Differentiation | calcium voltage-gated channel subunit alpha1 D |
| CACNA1D | 3 | 53328963 | 53813733 | Pancreatic Acinar Cell Carcinoma | calcium voltage-gated channel subunit alpha1 D |
| CACNA1D | 3 | 53328963 | 53813733 | Placental Choriocarcinoma | calcium voltage-gated channel subunit alpha1 D |
| CACNA1D | 3 | 53328963 | 53813733 | Small Intestinal Diffuse Large B-Cell Lymphoma | calcium voltage-gated channel subunit alpha1 D |
| CACNA1D | 3 | 53328963 | 53813733 | T-cell acute lymphoblastic leukemia | calcium voltage-gated channel subunit alpha1 D |
| CACNA1D | 3 | 53328963 | 53813733 | Thyroid Gland Undifferentiated (Anaplastic) Carcinoma | calcium voltage-gated channel subunit alpha1 D |
| CACNA1D | 3 | 53328963 | 53813733 | Acute lymphoblastic leukemia | calcium voltage-gated channel subunit alpha1 D |
| CACNA1D | 3 | 53328963 | 53813733 | Acute myeloid leukemia | calcium voltage-gated channel subunit alpha1 D |
| CACNA1D | 3 | 53328963 | 53813733 | Anaplastic astrocytoma | calcium voltage-gated channel subunit alpha1 D |
| CACNA1D | 3 | 53328963 | 53813733 | angiosarcoma | calcium voltage-gated channel subunit alpha1 D |
| CACNA1D | 3 | 53328963 | 53813733 | bile duct carcinoma | calcium voltage-gated channel subunit alpha1 D |
| CACNA1D | 3 | 53328963 | 53813733 | bladder transitional cell carcinoma | calcium voltage-gated channel subunit alpha1 D |
| CACNA1D | 3 | 53328963 | 53813733 | brain glioblastoma | calcium voltage-gated channel subunit alpha1 D |
| CACNA1D | 3 | 53328963 | 53813733 | Breast carcinoma | calcium voltage-gated channel subunit alpha1 D |
| CACNA1D | 3 | 53328963 | 53813733 | breast ductal adenocarcinoma | calcium voltage-gated channel subunit alpha1 D |
| CACNA1D | 3 | 53328963 | 53813733 | cecum adenocarcinoma | calcium voltage-gated channel subunit alpha1 D |
| CACNA1D | 3 | 53328963 | 53813733 | cervical squamous cell carcinoma | calcium voltage-gated channel subunit alpha1 D |
| CACNA1D | 3 | 53328963 | 53813733 | Chronic lymphocytic leukemia | calcium voltage-gated channel subunit alpha1 D |
| CACNA1D | 3 | 53328963 | 53813733 | Clear cell renal carcinoma | calcium voltage-gated channel subunit alpha1 D |
| CACNA1D | 3 | 53328963 | 53813733 | colon adenocarcinoma | calcium voltage-gated channel subunit alpha1 D |
| CACNA1D | 3 | 53328963 | 53813733 | colon carcinoma | calcium voltage-gated channel subunit alpha1 D |
| CACNA1D | 3 | 53328963 | 53813733 | colorectal adenocarcinoma | calcium voltage-gated channel subunit alpha1 D |
| CACNA1D | 3 | 53328963 | 53813733 | dedifferentiated chondrosarcoma | calcium voltage-gated channel subunit alpha1 D |
| CACNA1D | 3 | 53328963 | 53813733 | diffuse gastric adenocarcinoma | calcium voltage-gated channel subunit alpha1 D |
| CACNA1D | 3 | 53328963 | 53813733 | diffuse large B-cell lymphoma | calcium voltage-gated channel subunit alpha1 D |
| CACNA1D | 3 | 53328963 | 53813733 | Ependymoma | calcium voltage-gated channel subunit alpha1 D |
| CACNA1D | 3 | 53328963 | 53813733 | gastric adenocarcinoma | calcium voltage-gated channel subunit alpha1 D |
| CACNA1D | 3 | 53328963 | 53813733 | head and neck squamous cell carcinoma | calcium voltage-gated channel subunit alpha1 D |
| CACNA1D | 3 | 53328963 | 53813733 | Hepatocellular Carcinoma | calcium voltage-gated channel subunit alpha1 D |
| CACNA1D | 3 | 53328963 | 53813733 | kidney neoplasm | calcium voltage-gated channel subunit alpha1 D |
| CACNA1D | 3 | 53328963 | 53813733 | large cell lung carcinoma | calcium voltage-gated channel subunit alpha1 D |
| CACNA1D | 3 | 53328963 | 53813733 | large cell medulloblastoma | calcium voltage-gated channel subunit alpha1 D |
| CACNA1D | 3 | 53328963 | 53813733 | lobular breast carcinoma | calcium voltage-gated channel subunit alpha1 D |
| CACNA1D | 3 | 53328963 | 53813733 | Lung adenocarcinoma | calcium voltage-gated channel subunit alpha1 D |
| CACNA1D | 3 | 53328963 | 53813733 | Lung carcinoma | calcium voltage-gated channel subunit alpha1 D |
| CACNA1D | 3 | 53328963 | 53813733 | lymphoid neoplasm | calcium voltage-gated channel subunit alpha1 D |
| CACNA1D | 3 | 53328963 | 53813733 | mast-cell leukemia | calcium voltage-gated channel subunit alpha1 D |
| CACNA1D | 3 | 53328963 | 53813733 | Medulloblastoma | calcium voltage-gated channel subunit alpha1 D |
| CACNA1D | 3 | 53328963 | 53813733 | Melanoma | calcium voltage-gated channel subunit alpha1 D |
| CACNA1D | 3 | 53328963 | 53813733 | Neoplasm | calcium voltage-gated channel subunit alpha1 D |
| CACNA1D | 3 | 53328963 | 53813733 | neoplasm of mature B-cells | calcium voltage-gated channel subunit alpha1 D |
| CACNA1D | 3 | 53328963 | 53813733 | nodular melanoma | calcium voltage-gated channel subunit alpha1 D |
| CACNA1D | 3 | 53328963 | 53813733 | oral squamous cell carcinoma | calcium voltage-gated channel subunit alpha1 D |
| CACNA1D | 3 | 53328963 | 53813733 | OSTEOSARCOMA | calcium voltage-gated channel subunit alpha1 D |
| CACNA1D | 3 | 53328963 | 53813733 | ovarian adenocarcinoma | calcium voltage-gated channel subunit alpha1 D |
| CACNA1D | 3 | 53328963 | 53813733 | pancreatic carcinoma | calcium voltage-gated channel subunit alpha1 D |
| CACNA1D | 3 | 53328963 | 53813733 | pancreatic ductal adenocarcinoma | calcium voltage-gated channel subunit alpha1 D |
| CACNA1D | 3 | 53328963 | 53813733 | pancreatic neuroendocrine tumor | calcium voltage-gated channel subunit alpha1 D |
| CACNA1D | 3 | 53328963 | 53813733 | prostate adenocarcinoma | calcium voltage-gated channel subunit alpha1 D |
| CACNA1D | 3 | 53328963 | 53813733 | prostate carcinoma | calcium voltage-gated channel subunit alpha1 D |
| CACNA1D | 3 | 53328963 | 53813733 | pulmonary blastoma | calcium voltage-gated channel subunit alpha1 D |
| CACNA1D | 3 | 53328963 | 53813733 | rectal adenocarcinoma | calcium voltage-gated channel subunit alpha1 D |
| CACNA1D | 3 | 53328963 | 53813733 | skin carcinoma | calcium voltage-gated channel subunit alpha1 D |
| CACNA1D | 3 | 53328963 | 53813733 | Small cell lung carcinoma | calcium voltage-gated channel subunit alpha1 D |
| CACNA1D | 3 | 53328963 | 53813733 | soft tissue sarcoma | calcium voltage-gated channel subunit alpha1 D |
| CACNA1D | 3 | 53328963 | 53813733 | squamous cell lung carcinoma | calcium voltage-gated channel subunit alpha1 D |
| CACNA1D | 3 | 53328963 | 53813733 | testicular mixed germ cell tumor | calcium voltage-gated channel subunit alpha1 D |
| CACNA1D | 3 | 53328963 | 53813733 | testicular seminoma | calcium voltage-gated channel subunit alpha1 D |
| CACNA1D | 3 | 53328963 | 53813733 | thyroid carcinoma | calcium voltage-gated channel subunit alpha1 D |
| CACNA1D | 3 | 53328963 | 53813733 | thyroid neoplasm | calcium voltage-gated channel subunit alpha1 D |
| CACNA1D | 3 | 53328963 | 53813733 | Well-differentiated liposarcoma | calcium voltage-gated channel subunit alpha1 D |
| CACNA2D3 | 3 | 54122552 | 55074557 |  | calcium voltage-gated channel auxiliary subunit alpha2delta 3 |
| UGT2A3 | 4 | 68928463 | 68951804 |  | UDP glucuronosyltransferase family 2 member A3 |
| PDLIM5 | 4 | 94451857 | 94668227 |  | PDZ and LIM domain 5 |
| GABRA2 | 4 | 46243548 | 46475230 | Non-specific early-onset epileptic encephalopathy | gamma-aminobutyric acid type A receptor subunit alpha2 |
| GABRA2 | 4 | 46243548 | 46475230 | Alcohol dependence | gamma-aminobutyric acid type A receptor subunit alpha2 |
| GABRA2 | 4 | 46243548 | 46475230 | DEVELOPMENTAL AND EPILEPTIC ENCEPHALOPATHY 78 | gamma-aminobutyric acid type A receptor subunit alpha2 |
| COL25A1 | 4 | 108808725 | 109302752 | Congenital ptosis | collagen type XXV alpha 1 chain |
| COL25A1 | 4 | 108808725 | 109302752 | Neurogenic arthrogryposis multiplex congenita | collagen type XXV alpha 1 chain |
| COL25A1 | 4 | 108808725 | 109302752 | Fibrosis of extraocular muscles congenital 5 | collagen type XXV alpha 1 chain |
| PDE5A | 4 | 119494397 | 119628804 |  | phosphodiesterase 5A |
| GALNTL6 | 4 | 171812254 | 173041559 |  | polypeptide N-acetylgalactosaminyltransferase like 6 |
| ADAMTS16 | 5 | 5140330 | 5320304 |  | ADAM metallopeptidase with thrombospondin type 1 motif 16 |
| ADAMTS12 | 5 | 33523535 | 33892019 |  | ADAM metallopeptidase with thrombospondin type 1 motif 12 |
| UGT3A1 | 5 | 35951006 | 36001028 |  | UDP glycosyltransferase family 3 member A1 |
| GALNT10 | 5 | 154190730 | 154420984 |  | polypeptide N-acetylgalactosaminyltransferase 10 |
| GABRB2 | 5 | 161288429 | 161549044 | Non-specific early-onset epileptic encephalopathy | gamma-aminobutyric acid type A receptor subunit beta2 |
| GABRB2 | 5 | 161288429 | 161549044 | Developmental and epileptic encephalopathy 92 | gamma-aminobutyric acid type A receptor subunit beta2 |
| GABRB2 | 5 | 161288429 | 161549044 | Epilepsy and intellectual disability | gamma-aminobutyric acid type A receptor subunit beta2 |
| COL23A1 | 5 | 178237476 | 178590393 |  | collagen type XXIII alpha 1 chain |
| ADAMTS19 | 5 | 129460281 | 129738683 | Cardiac valvular dysplasia 2 | ADAM metallopeptidase with thrombospondin type 1 motif 19 |
| ADAMTS6 | 5 | 65148738 | 65481920 |  | ADAM metallopeptidase with thrombospondin type 1 motif 6 |
| PRKAA1 | 5 | 40759389 | 40798374 |  | protein kinase AMP-activated catalytic subunit alpha 1 |
| SLC6A3 | 5 | 1392794 | 1445440 | Infantile dystonia-parkinsonism | solute carrier family 6 member 3 |
| SLC6A3 | 5 | 1392794 | 1445440 | PARKINSONISM-DYSTONIA 1 INFANTILE-ONSET | solute carrier family 6 member 3 |
| SLC6A3 | 5 | 1392794 | 1445440 | TOBACCO ADDICTION SUSCEPTIBILITY TO | solute carrier family 6 member 3 |
| FLOT1 | 6 | 30727709 | 30742732 |  | flotillin 1 |
| GRIK2 | 6 | 100962701 | 102081622 | Autosomal recessive non-syndromic intellectual disability | glutamate ionotropic receptor kainate type subunit 2 |
| GRIK2 | 6 | 100962701 | 102081622 | INTELLECTUAL DEVELOPMENTAL DISORDER AUTOSOMAL RECESSIVE 6 | glutamate ionotropic receptor kainate type subunit 2 |
| GRIK2 | 6 | 100962701 | 102081622 | NEURODEVELOPMENTAL DISORDER WITH IMPAIRED LANGUAGE AND ATAXIA AND WITH OR WITHOUT SEIZURES | glutamate ionotropic receptor kainate type subunit 2 |
| GRIK2 | 6 | 100962701 | 102081622 | GRIK2-related intellectual disability and hypomyelination | glutamate ionotropic receptor kainate type subunit 2 |
| GRIK2 | 6 | 100962701 | 102081622 | INTELLECTUAL DEVELOPMENTAL DISORDER AUTOSOMAL RECESSIVE TYPE 6 | glutamate ionotropic receptor kainate type subunit 2 |
| PDE10A | 6 | 165327287 | 165988117 | Childhood-onset benign chorea with striatal involvement | phosphodiesterase 10A |
| PDE10A | 6 | 165327287 | 165988117 | Infantile-onset generalized dyskinesia with orofacial involvement | phosphodiesterase 10A |
| PDE10A | 6 | 165327287 | 165988117 | DYSKINESIA LIMB AND OROFACIAL INFANTILE-ONSET | phosphodiesterase 10A |
| PDE10A | 6 | 165327287 | 165988117 | Striatal degeneration autosomal dominant 2 | phosphodiesterase 10A |
| PDE10A | 6 | 165327287 | 165988117 | Childhood-Onset Chorea with Bilateral Striatal Lesions | phosphodiesterase 10A |
| PDE10A | 6 | 165327287 | 165988117 | Childhood-onset benign chorea with striatal involvement | long intergenic non-protein coding RNA 473 |
| PDE10A | 6 | 165327287 | 165988117 | Infantile-onset generalized dyskinesia with orofacial involvement | long intergenic non-protein coding RNA 473 |
| PDE10A | 6 | 165327287 | 165988117 | DYSKINESIA LIMB AND OROFACIAL INFANTILE-ONSET | long intergenic non-protein coding RNA 473 |
| PDE10A | 6 | 165327287 | 165988117 | Striatal degeneration autosomal dominant 2 | long intergenic non-protein coding RNA 473 |
| PDE10A | 6 | 165327287 | 165988117 | Childhood-Onset Chorea with Bilateral Striatal Lesions | long intergenic non-protein coding RNA 473 |
| MYO6 | 6 | 75749201 | 75919537 | Autosomal dominant non-syndromic sensorineural deafness type DFNA | myosin VI |
| MYO6 | 6 | 75749201 | 75919537 | Autosomal recessive non-syndromic sensorineural deafness type DFNB | myosin VI |
| MYO6 | 6 | 75749201 | 75919537 | Progressive sensorineural hearing loss-hypertrophic cardiomyopathy syndrome | myosin VI |
| MYO6 | 6 | 75749201 | 75919537 | DEAFNESS AUTOSOMAL DOMINANT 22 | myosin VI |
| MYO6 | 6 | 75749201 | 75919537 | DEAFNESS AUTOSOMAL RECESSIVE 37 | myosin VI |
| COL9A1 | 6 | 70216040 | 70303084 | Autosomal recessive Stickler syndrome | collagen type IX alpha 1 chain |
| COL9A1 | 6 | 70216040 | 70303084 | Multiple epiphyseal dysplasia due to collagen 9 anomaly | collagen type IX alpha 1 chain |
| COL9A1 | 6 | 70216040 | 70303084 | EPIPHYSEAL DYSPLASIA MULTIPLE 6 | collagen type IX alpha 1 chain |
| COL9A1 | 6 | 70216040 | 70303084 | STICKLER SYNDROME TYPE IV | collagen type IX alpha 1 chain |
| COL9A1 | 6 | 70216040 | 70303084 | MULTIPLE EPIPHYSEAL DYSPLASIA TYPE 6 | collagen type IX alpha 1 chain |
| COL9A1 | 6 | 70216040 | 70303084 | STICKLER SYNDROME TYPE 4 | collagen type IX alpha 1 chain |
| GRM1 | 6 | 146027646 | 146437601 | Autosomal recessive congenital cerebellar ataxia due to MGLUR1 deficiency | glutamate metabotropic receptor 1 |
| GRM1 | 6 | 146027646 | 146437601 | Chondromyxoid fibroma | glutamate metabotropic receptor 1 |
| GRM1 | 6 | 146027646 | 146437601 | Spinocerebellar ataxia type 44 | glutamate metabotropic receptor 1 |
| GRM1 | 6 | 146027646 | 146437601 | SPINOCEREBELLAR ATAXIA 44 | glutamate metabotropic receptor 1 |
| GRM1 | 6 | 146027646 | 146437601 | SPINOCEREBELLAR ATAXIA AUTOSOMAL RECESSIVE 13 | glutamate metabotropic receptor 1 |
| GRM1 | 6 | 146027646 | 146437601 | Congenital Cerebellar Ataxia | glutamate metabotropic receptor 1 |
| C1GALT1 | 7 | 7156934 | 7248616 |  | core 1 synthase, glycoprotein-N-acetylgalactosamine 3-beta-galactosyltransferase 1 |
| COL26A1 | 7 | 101362875 | 101559024 |  | collagen type XXVI alpha 1 chain |
| CACNA2D1 | 7 | 81946444 | 82443956 | Brugada syndrome | calcium voltage-gated channel auxiliary subunit alpha2delta 1 |
| CACNA2D1 | 7 | 81946444 | 82443956 | Familial short QT syndrome | calcium voltage-gated channel auxiliary subunit alpha2delta 1 |
| CACNA2D1 | 7 | 81946444 | 82443956 | Non-specific early-onset epileptic encephalopathy | calcium voltage-gated channel auxiliary subunit alpha2delta 1 |
| CACNA2D1 | 7 | 81946444 | 82443956 | Developmental and epileptic encephalopathy 110 | calcium voltage-gated channel auxiliary subunit alpha2delta 1 |
| GALNT17 | 7 | 71132144 | 71713600 |  | polypeptide N-acetylgalactosaminyltransferase 17 |
| PRKAG2 | 7 | 151556124 | 151877214 | Fatal congenital hypertrophic cardiomyopathy due to glycogen storage disease | protein kinase AMP-activated non-catalytic subunit gamma 2 |
| PRKAG2 | 7 | 151556124 | 151877214 | NON RARE IN EUROPE: Familial isolated hypertrophic cardiomyopathy | protein kinase AMP-activated non-catalytic subunit gamma 2 |
| PRKAG2 | 7 | 151556124 | 151877214 | NON RARE IN EUROPE: Wolff-Parkinson-White syndrome | protein kinase AMP-activated non-catalytic subunit gamma 2 |
| PRKAG2 | 7 | 151556124 | 151877214 | CARDIOMYOPATHY FAMILIAL HYPERTROPHIC 6 | protein kinase AMP-activated non-catalytic subunit gamma 2 |
| PRKAG2 | 7 | 151556124 | 151877214 | GLYCOGEN STORAGE DISEASE OF HEART LETHAL CONGENITAL | protein kinase AMP-activated non-catalytic subunit gamma 2 |
| PRKAG2 | 7 | 151556124 | 151877214 | WOLFF-PARKINSON-WHITE SYNDROME | protein kinase AMP-activated non-catalytic subunit gamma 2 |
| PRKAG2 | 7 | 151556124 | 151877214 | PRKAG2-related cardiomyopathy | protein kinase AMP-activated non-catalytic subunit gamma 2 |
| KCNQ3 | 8 | 132120861 | 132481095 | Benign familial infantile epilepsy | potassium voltage-gated channel subfamily Q member 3 |
| KCNQ3 | 8 | 132120861 | 132481095 | Benign familial neonatal epilepsy | potassium voltage-gated channel subfamily Q member 3 |
| KCNQ3 | 8 | 132120861 | 132481095 | Juvenile myoclonic epilepsy | potassium voltage-gated channel subfamily Q member 3 |
| KCNQ3 | 8 | 132120861 | 132481095 | SEIZURES BENIGN FAMILIAL NEONATAL 2 | potassium voltage-gated channel subfamily Q member 3 |
| KCNQ3 | 8 | 132120861 | 132481095 | KCNQ3 syndrome | potassium voltage-gated channel subfamily Q member 3 |
| ST3GAL1 | 8 | 133454848 | 133571926 |  | ST3 beta-galactoside alpha-2,3-sialyltransferase 1 |
| LAMC3 | 9 | 131009174 | 131094473 | Occipital pachygyria and polymicrogyria | laminin subunit gamma 3 |
| LAMC3 | 9 | 131009174 | 131094473 | CORTICAL MALFORMATIONS OCCIPITAL | laminin subunit gamma 3 |
| LAMC3 | 9 | 131009174 | 131094473 | occipital cortical malformations | laminin subunit gamma 3 |
| ADAMTSL1 | 9 | 17906563 | 18910950 | Microcephaly-facial dysmorphism-ocular anomalies-multiple congenital anomalies syndrome | ADAMTS like 1 |
| ADAMTSL1 | 9 | 17906563 | 18910950 | Syndromic congenital glaucoma | ADAMTS like 1 |
| PTPRD | 9 | 8314246 | 10613002 | Atypical Meningioma | protein tyrosine phosphatase receptor type D |
| PTPRD | 9 | 8314246 | 10613002 | B-cell acute lymphoblastic leukemia | protein tyrosine phosphatase receptor type D |
| PTPRD | 9 | 8314246 | 10613002 | Bladder Small Cell Neuroendocrine Carcinoma | protein tyrosine phosphatase receptor type D |
| PTPRD | 9 | 8314246 | 10613002 | Borderline Ovarian Mucinous Tumor | protein tyrosine phosphatase receptor type D |
| PTPRD | 9 | 8314246 | 10613002 | Brain Stem Glioblastoma | protein tyrosine phosphatase receptor type D |
| PTPRD | 9 | 8314246 | 10613002 | Breast Carcinoma by Gene Expression Profile | protein tyrosine phosphatase receptor type D |
| PTPRD | 9 | 8314246 | 10613002 | Burkitts lymphoma | protein tyrosine phosphatase receptor type D |
| PTPRD | 9 | 8314246 | 10613002 | Digestive System Carcinoma | protein tyrosine phosphatase receptor type D |
| PTPRD | 9 | 8314246 | 10613002 | Duodenal Adenocarcinoma | protein tyrosine phosphatase receptor type D |
| PTPRD | 9 | 8314246 | 10613002 | Endometrial Endometrioid Adenocarcinoma | protein tyrosine phosphatase receptor type D |
| PTPRD | 9 | 8314246 | 10613002 | Gastric Adenoma | protein tyrosine phosphatase receptor type D |
| PTPRD | 9 | 8314246 | 10613002 | Hepatobiliary Neoplasm | protein tyrosine phosphatase receptor type D |
| PTPRD | 9 | 8314246 | 10613002 | Hodgkins lymphoma | protein tyrosine phosphatase receptor type D |
| PTPRD | 9 | 8314246 | 10613002 | Invasive Breast Carcinoma | protein tyrosine phosphatase receptor type D |
| PTPRD | 9 | 8314246 | 10613002 | MANTLE CELL LYMPHOMA | protein tyrosine phosphatase receptor type D |
| PTPRD | 9 | 8314246 | 10613002 | Merkel cell skin cancer | protein tyrosine phosphatase receptor type D |
| PTPRD | 9 | 8314246 | 10613002 | ND | protein tyrosine phosphatase receptor type D |
| PTPRD | 9 | 8314246 | 10613002 | Ovarian Endometrioid Adenocarcinoma | protein tyrosine phosphatase receptor type D |
| PTPRD | 9 | 8314246 | 10613002 | Ovarian Endometrioid Adenocarcinoma with Squamous Differentiation | protein tyrosine phosphatase receptor type D |
| PTPRD | 9 | 8314246 | 10613002 | Pancreatic Acinar Cell Carcinoma | protein tyrosine phosphatase receptor type D |
| PTPRD | 9 | 8314246 | 10613002 | Placental Choriocarcinoma | protein tyrosine phosphatase receptor type D |
| PTPRD | 9 | 8314246 | 10613002 | Secretory Meningioma | protein tyrosine phosphatase receptor type D |
| PTPRD | 9 | 8314246 | 10613002 | Signet Ring Cell Gastric Adenocarcinoma | protein tyrosine phosphatase receptor type D |
| PTPRD | 9 | 8314246 | 10613002 | Sinonasal Undifferentiated Carcinoma | protein tyrosine phosphatase receptor type D |
| PTPRD | 9 | 8314246 | 10613002 | Submandibular Gland Adenoid Cystic Carcinoma | protein tyrosine phosphatase receptor type D |
| PTPRD | 9 | 8314246 | 10613002 | T-cell acute lymphoblastic leukemia | protein tyrosine phosphatase receptor type D |
| PTPRD | 9 | 8314246 | 10613002 | Thyroid Gland Undifferentiated (Anaplastic) Carcinoma | protein tyrosine phosphatase receptor type D |
| PTPRD | 9 | 8314246 | 10613002 | Unclassified Renal Cell Carcinoma | protein tyrosine phosphatase receptor type D |
| PTPRD | 9 | 8314246 | 10613002 | Acute myeloid leukemia | protein tyrosine phosphatase receptor type D |
| PTPRD | 9 | 8314246 | 10613002 | adenosquamous lung carcinoma | protein tyrosine phosphatase receptor type D |
| PTPRD | 9 | 8314246 | 10613002 | adrenal gland pheochromocytoma | protein tyrosine phosphatase receptor type D |
| PTPRD | 9 | 8314246 | 10613002 | Anaplastic astrocytoma | protein tyrosine phosphatase receptor type D |
| PTPRD | 9 | 8314246 | 10613002 | anaplastic large cell lymphoma | protein tyrosine phosphatase receptor type D |
| PTPRD | 9 | 8314246 | 10613002 | angiosarcoma | protein tyrosine phosphatase receptor type D |
| PTPRD | 9 | 8314246 | 10613002 | bile duct carcinoma | protein tyrosine phosphatase receptor type D |
| PTPRD | 9 | 8314246 | 10613002 | bladder transitional cell carcinoma | protein tyrosine phosphatase receptor type D |
| PTPRD | 9 | 8314246 | 10613002 | brain glioblastoma | protein tyrosine phosphatase receptor type D |
| PTPRD | 9 | 8314246 | 10613002 | Breast carcinoma | protein tyrosine phosphatase receptor type D |
| PTPRD | 9 | 8314246 | 10613002 | breast ductal adenocarcinoma | protein tyrosine phosphatase receptor type D |
| PTPRD | 9 | 8314246 | 10613002 | carcinoid tumor | protein tyrosine phosphatase receptor type D |
| PTPRD | 9 | 8314246 | 10613002 | cecum adenocarcinoma | protein tyrosine phosphatase receptor type D |
| PTPRD | 9 | 8314246 | 10613002 | cervical adenocarcinoma | protein tyrosine phosphatase receptor type D |
| PTPRD | 9 | 8314246 | 10613002 | cervical squamous cell carcinoma | protein tyrosine phosphatase receptor type D |
| PTPRD | 9 | 8314246 | 10613002 | Chronic lymphocytic leukemia | protein tyrosine phosphatase receptor type D |
| PTPRD | 9 | 8314246 | 10613002 | Clear cell renal carcinoma | protein tyrosine phosphatase receptor type D |
| PTPRD | 9 | 8314246 | 10613002 | colon adenocarcinoma | protein tyrosine phosphatase receptor type D |
| PTPRD | 9 | 8314246 | 10613002 | colon carcinoma | protein tyrosine phosphatase receptor type D |
| PTPRD | 9 | 8314246 | 10613002 | colonic neoplasm | protein tyrosine phosphatase receptor type D |
| PTPRD | 9 | 8314246 | 10613002 | colorectal adenocarcinoma | protein tyrosine phosphatase receptor type D |
| PTPRD | 9 | 8314246 | 10613002 | diffuse gastric adenocarcinoma | protein tyrosine phosphatase receptor type D |
| PTPRD | 9 | 8314246 | 10613002 | diffuse large B-cell lymphoma | protein tyrosine phosphatase receptor type D |
| PTPRD | 9 | 8314246 | 10613002 | Ependymoma | protein tyrosine phosphatase receptor type D |
| PTPRD | 9 | 8314246 | 10613002 | gastric adenocarcinoma | protein tyrosine phosphatase receptor type D |
| PTPRD | 9 | 8314246 | 10613002 | head and neck squamous cell carcinoma | protein tyrosine phosphatase receptor type D |
| PTPRD | 9 | 8314246 | 10613002 | Hepatocellular Carcinoma | protein tyrosine phosphatase receptor type D |
| PTPRD | 9 | 8314246 | 10613002 | kidney neoplasm | protein tyrosine phosphatase receptor type D |
| PTPRD | 9 | 8314246 | 10613002 | large cell lung carcinoma | protein tyrosine phosphatase receptor type D |
| PTPRD | 9 | 8314246 | 10613002 | large cell medulloblastoma | protein tyrosine phosphatase receptor type D |
| PTPRD | 9 | 8314246 | 10613002 | leiomyosarcoma | protein tyrosine phosphatase receptor type D |
| PTPRD | 9 | 8314246 | 10613002 | lobular breast carcinoma | protein tyrosine phosphatase receptor type D |
| PTPRD | 9 | 8314246 | 10613002 | Lung adenocarcinoma | protein tyrosine phosphatase receptor type D |
| PTPRD | 9 | 8314246 | 10613002 | Lung carcinoma | protein tyrosine phosphatase receptor type D |
| PTPRD | 9 | 8314246 | 10613002 | lymphoid neoplasm | protein tyrosine phosphatase receptor type D |
| PTPRD | 9 | 8314246 | 10613002 | malignant epithelioid mesothelioma | protein tyrosine phosphatase receptor type D |
| PTPRD | 9 | 8314246 | 10613002 | marginal zone B-cell lymphoma | protein tyrosine phosphatase receptor type D |
| PTPRD | 9 | 8314246 | 10613002 | mast-cell leukemia | protein tyrosine phosphatase receptor type D |
| PTPRD | 9 | 8314246 | 10613002 | Medulloblastoma | protein tyrosine phosphatase receptor type D |
| PTPRD | 9 | 8314246 | 10613002 | Melanoma | protein tyrosine phosphatase receptor type D |
| PTPRD | 9 | 8314246 | 10613002 | Multiple myeloma | protein tyrosine phosphatase receptor type D |
| PTPRD | 9 | 8314246 | 10613002 | Neoplasm | protein tyrosine phosphatase receptor type D |
| PTPRD | 9 | 8314246 | 10613002 | neoplasm of mature B-cells | protein tyrosine phosphatase receptor type D |
| PTPRD | 9 | 8314246 | 10613002 | nodular melanoma | protein tyrosine phosphatase receptor type D |
| PTPRD | 9 | 8314246 | 10613002 | non-small cell lung carcinoma | protein tyrosine phosphatase receptor type D |
| PTPRD | 9 | 8314246 | 10613002 | oral squamous cell carcinoma | protein tyrosine phosphatase receptor type D |
| PTPRD | 9 | 8314246 | 10613002 | OSTEOSARCOMA | protein tyrosine phosphatase receptor type D |
| PTPRD | 9 | 8314246 | 10613002 | ovarian clear cell adenocarcinoma | protein tyrosine phosphatase receptor type D |
| PTPRD | 9 | 8314246 | 10613002 | ovarian mucinous adenocarcinoma | protein tyrosine phosphatase receptor type D |
| PTPRD | 9 | 8314246 | 10613002 | pancreatic carcinoma | protein tyrosine phosphatase receptor type D |
| PTPRD | 9 | 8314246 | 10613002 | pancreatic ductal adenocarcinoma | protein tyrosine phosphatase receptor type D |
| PTPRD | 9 | 8314246 | 10613002 | pancreatic neoplasm | protein tyrosine phosphatase receptor type D |
| PTPRD | 9 | 8314246 | 10613002 | pancreatic neuroendocrine tumor | protein tyrosine phosphatase receptor type D |
| PTPRD | 9 | 8314246 | 10613002 | pharyngeal squamous cell carcinoma | protein tyrosine phosphatase receptor type D |
| PTPRD | 9 | 8314246 | 10613002 | prostate adenocarcinoma | protein tyrosine phosphatase receptor type D |
| PTPRD | 9 | 8314246 | 10613002 | prostate carcinoma | protein tyrosine phosphatase receptor type D |
| PTPRD | 9 | 8314246 | 10613002 | pulmonary blastoma | protein tyrosine phosphatase receptor type D |
| PTPRD | 9 | 8314246 | 10613002 | rectal adenocarcinoma | protein tyrosine phosphatase receptor type D |
| PTPRD | 9 | 8314246 | 10613002 | salivary gland squamous cell carcinoma | protein tyrosine phosphatase receptor type D |
| PTPRD | 9 | 8314246 | 10613002 | skin carcinoma | protein tyrosine phosphatase receptor type D |
| PTPRD | 9 | 8314246 | 10613002 | Small cell lung carcinoma | protein tyrosine phosphatase receptor type D |
| PTPRD | 9 | 8314246 | 10613002 | soft tissue sarcoma | protein tyrosine phosphatase receptor type D |
| PTPRD | 9 | 8314246 | 10613002 | squamous cell lung carcinoma | protein tyrosine phosphatase receptor type D |
| PTPRD | 9 | 8314246 | 10613002 | testicular mixed germ cell tumor | protein tyrosine phosphatase receptor type D |
| PTPRD | 9 | 8314246 | 10613002 | testicular seminoma | protein tyrosine phosphatase receptor type D |
| PTPRD | 9 | 8314246 | 10613002 | thyroid carcinoma | protein tyrosine phosphatase receptor type D |
| PTPRD | 9 | 8314246 | 10613002 | thyroid neoplasm | protein tyrosine phosphatase receptor type D |
| PTPRD | 9 | 8314246 | 10613002 | undifferentiated pleomorphic sarcoma | protein tyrosine phosphatase receptor type D |
| PTPRD | 9 | 8314246 | 10613002 | urothelial carcinoma | protein tyrosine phosphatase receptor type D |
| PTPRD | 9 | 8314246 | 10613002 | Well-differentiated liposarcoma | protein tyrosine phosphatase receptor type D |
| PRKG1 | 10 | 50990888 | 52298423 | Familial thoracic aortic aneurysm and aortic dissection | protein kinase cGMP-dependent 1 |
| PRKG1 | 10 | 50990888 | 52298423 | Aortic aneurysm familial thoracic 8 | protein kinase cGMP-dependent 1 |
| CACNB2 | 10 | 18140424 | 18543557 | Brugada syndrome | calcium voltage-gated channel auxiliary subunit beta 2 |
| CACNB2 | 10 | 18140424 | 18543557 | BRUGADA SYNDROME 4 | calcium voltage-gated channel auxiliary subunit beta 2 |
| COL13A1 | 10 | 69801880 | 69964275 | Postsynaptic congenital myasthenic syndromes | collagen type XIII alpha 1 chain |
| COL13A1 | 10 | 69801880 | 69964275 | Presynaptic congenital myasthenic syndromes | collagen type XIII alpha 1 chain |
| COL13A1 | 10 | 69801880 | 69964275 | Myasthenic syndrome congenital 19 | collagen type XIII alpha 1 chain |
| COL13A1 | 10 | 69801880 | 69964275 | Congenital Myasthenic Syndrome Type 19 | collagen type XIII alpha 1 chain |
| KCNMA1 | 10 | 76869601 | 77638369 | Generalized epilepsy-paroxysmal dyskinesia syndrome | potassium calcium-activated channel subfamily M alpha 1 |
| KCNMA1 | 10 | 76869601 | 77638369 | Non-specific syndromic intellectual disability | potassium calcium-activated channel subfamily M alpha 1 |
| KCNMA1 | 10 | 76869601 | 77638369 | CEREBELLAR ATROPHY DEVELOPMENTAL DELAY AND SEIZURES | potassium calcium-activated channel subfamily M alpha 1 |
| KCNMA1 | 10 | 76869601 | 77638369 | EPILEPSY IDIOPATHIC GENERALIZED SUSCEPTIBILITY TO 16 | potassium calcium-activated channel subfamily M alpha 1 |
| KCNMA1 | 10 | 76869601 | 77638369 | LIANG-WANG SYNDROME | potassium calcium-activated channel subfamily M alpha 1 |
| KCNMA1 | 10 | 76869601 | 77638369 | PAROXYSMAL NONKINESIGENIC DYSKINESIA 3 WITH OR WITHOUT GENERALIZED EPILEPSY | potassium calcium-activated channel subfamily M alpha 1 |
| KCNMA1 | 10 | 76869601 | 77638369 | GENERALIZED EPILEPSY AND PAROXYSMAL DYSKINESIA | potassium calcium-activated channel subfamily M alpha 1 |
| KCNMA1 | 10 | 76869601 | 77638369 | KCNMA1-related developmental delay seizures and cerebellar atrophy | potassium calcium-activated channel subfamily M alpha 1 |
| GALNT18 | 11 | 11270877 | 11622005 |  | polypeptide N-acetylgalactosaminyltransferase 18 |
| SYT9 | 11 | 7238778 | 7469043 |  | synaptotagmin 9 |
| GUCY1A2 | 11 | 106674019 | 107018476 |  | guanylate cyclase 1 soluble subunit alpha 2 |
| GRIK4 | 11 | 120511746 | 120988906 |  | glutamate ionotropic receptor kainate type subunit 4 |
| GRM5 | 11 | 88504576 | 89065982 |  | glutamate metabotropic receptor 5 |
| HTR3B | 11 | 113904796 | 113949079 |  | 5-hydroxytryptamine receptor 3B |
| SHANK2 | 11 | 70467854 | 71252577 | NON RARE IN EUROPE | SH3 and multiple ankyrin repeat domains 2 |
| SHANK2 | 11 | 70467854 | 71252577 | AUTISM SUSCEPTIBILITY TO 17 | SH3 and multiple ankyrin repeat domains 2 |
| SHANK2 | 11 | 70467854 | 71252577 | SUSCEPTIBILITY TO AUTISM TYPE 17 | SH3 and multiple ankyrin repeat domains 2 |
| AP2A2 | 11 | 924881 | 1012245 |  | adaptor related protein complex 2 subunit alpha 2 |
| DLG2 | 11 | 83455012 | 85628335 |  | discs large MAGUK scaffold protein 2 |
| GRIA4 | 11 | 105609535 | 105982090 | Non-specific syndromic intellectual disability | glutamate ionotropic receptor AMPA type subunit 4 |
| GRIA4 | 11 | 105609535 | 105982090 | NEURODEVELOPMENTAL DISORDER WITH OR WITHOUT SEIZURES AND GAIT ABNORMALITIES | glutamate ionotropic receptor AMPA type subunit 4 |
| KCNQ1 | 11 | 2444654 | 2849105 | Familial atrial fibrillation | potassium voltage-gated channel subfamily Q member 1 |
| KCNQ1 | 11 | 2444654 | 2849105 | Familial short QT syndrome | potassium voltage-gated channel subfamily Q member 1 |
| KCNQ1 | 11 | 2444654 | 2849105 | Jervell and Lange-Nielsen syndrome | potassium voltage-gated channel subfamily Q member 1 |
| KCNQ1 | 11 | 2444654 | 2849105 | Romano-Ward syndrome | potassium voltage-gated channel subfamily Q member 1 |
| KCNQ1 | 11 | 2444654 | 2849105 | ATRIAL FIBRILLATION FAMILIAL 3 | potassium voltage-gated channel subfamily Q member 1 |
| KCNQ1 | 11 | 2444654 | 2849105 | BECKWITH-WIEDEMANN SYNDROME | potassium voltage-gated channel subfamily Q member 1 |
| KCNQ1 | 11 | 2444654 | 2849105 | JERVELL AND LANGE-NIELSEN SYNDROME 1 | potassium voltage-gated channel subfamily Q member 1 |
| KCNQ1 | 11 | 2444654 | 2849105 | LONG QT SYNDROME 1 | potassium voltage-gated channel subfamily Q member 1 |
| KCNQ1 | 11 | 2444654 | 2849105 | SHORT QT SYNDROME 2 | potassium voltage-gated channel subfamily Q member 1 |
| KCNQ1 | 11 | 2444654 | 2849105 | Jervell and Lange-Nielsen syndrome type 1 | potassium voltage-gated channel subfamily Q member 1 |
| KCNQ1 | 11 | 2444654 | 2849105 | KCNQ1-related JLNS | potassium voltage-gated channel subfamily Q member 1 |
| KCNQ1 | 11 | 2444654 | 2849105 | KCNQ1-related LQTS | potassium voltage-gated channel subfamily Q member 1 |
| KCNQ1 | 11 | 2444654 | 2849105 | KCNQ1-related SQTS | potassium voltage-gated channel subfamily Q member 1 |
| NCAM1 | 11 | 112961275 | 113278436 |  | neural cell adhesion molecule 1 |
| PPFIBP1 | 12 | 27523431 | 27695564 | Neurodevelopmental disorder with seizures microcephaly and brain abnormalities | PPFIA binding protein 1 |
| PPFIBP1 | 12 | 27523431 | 27695564 | PPFIBP1-related neurodevelopmental disorder | PPFIA binding protein 1 |
| PPFIBP1 | 12 | 27523431 | 27695564 | Atypical Meningioma | PPFIA binding protein 1 |
| PPFIBP1 | 12 | 27523431 | 27695564 | B-cell acute lymphoblastic leukemia | PPFIA binding protein 1 |
| PPFIBP1 | 12 | 27523431 | 27695564 | Brain Stem Glioblastoma | PPFIA binding protein 1 |
| PPFIBP1 | 12 | 27523431 | 27695564 | Burkitts lymphoma | PPFIA binding protein 1 |
| PPFIBP1 | 12 | 27523431 | 27695564 | Endometrial Endometrioid Adenocarcinoma | PPFIA binding protein 1 |
| PPFIBP1 | 12 | 27523431 | 27695564 | Gastric Adenoma | PPFIA binding protein 1 |
| PPFIBP1 | 12 | 27523431 | 27695564 | Hepatobiliary Neoplasm | PPFIA binding protein 1 |
| PPFIBP1 | 12 | 27523431 | 27695564 | Hodgkins lymphoma | PPFIA binding protein 1 |
| PPFIBP1 | 12 | 27523431 | 27695564 | MANTLE CELL LYMPHOMA | PPFIA binding protein 1 |
| PPFIBP1 | 12 | 27523431 | 27695564 | Merkel cell skin cancer | PPFIA binding protein 1 |
| PPFIBP1 | 12 | 27523431 | 27695564 | ND | PPFIA binding protein 1 |
| PPFIBP1 | 12 | 27523431 | 27695564 | Ovarian Endometrioid Adenocarcinoma with Squamous Differentiation | PPFIA binding protein 1 |
| PPFIBP1 | 12 | 27523431 | 27695564 | Pancreatic Acinar Cell Carcinoma | PPFIA binding protein 1 |
| PPFIBP1 | 12 | 27523431 | 27695564 | Placental Choriocarcinoma | PPFIA binding protein 1 |
| PPFIBP1 | 12 | 27523431 | 27695564 | T-cell acute lymphoblastic leukemia | PPFIA binding protein 1 |
| PPFIBP1 | 12 | 27523431 | 27695564 | Acute lymphoblastic leukemia | PPFIA binding protein 1 |
| PPFIBP1 | 12 | 27523431 | 27695564 | Acute myeloid leukemia | PPFIA binding protein 1 |
| PPFIBP1 | 12 | 27523431 | 27695564 | Anaplastic astrocytoma | PPFIA binding protein 1 |
| PPFIBP1 | 12 | 27523431 | 27695564 | Basal cell carcinoma | PPFIA binding protein 1 |
| PPFIBP1 | 12 | 27523431 | 27695564 | bile duct carcinoma | PPFIA binding protein 1 |
| PPFIBP1 | 12 | 27523431 | 27695564 | bladder transitional cell carcinoma | PPFIA binding protein 1 |
| PPFIBP1 | 12 | 27523431 | 27695564 | brain glioblastoma | PPFIA binding protein 1 |
| PPFIBP1 | 12 | 27523431 | 27695564 | Breast carcinoma | PPFIA binding protein 1 |
| PPFIBP1 | 12 | 27523431 | 27695564 | breast ductal adenocarcinoma | PPFIA binding protein 1 |
| PPFIBP1 | 12 | 27523431 | 27695564 | cervical squamous cell carcinoma | PPFIA binding protein 1 |
| PPFIBP1 | 12 | 27523431 | 27695564 | Chronic lymphocytic leukemia | PPFIA binding protein 1 |
| PPFIBP1 | 12 | 27523431 | 27695564 | Clear cell renal carcinoma | PPFIA binding protein 1 |
| PPFIBP1 | 12 | 27523431 | 27695564 | colon adenocarcinoma | PPFIA binding protein 1 |
| PPFIBP1 | 12 | 27523431 | 27695564 | colon carcinoma | PPFIA binding protein 1 |
| PPFIBP1 | 12 | 27523431 | 27695564 | colorectal adenocarcinoma | PPFIA binding protein 1 |
| PPFIBP1 | 12 | 27523431 | 27695564 | diffuse gastric adenocarcinoma | PPFIA binding protein 1 |
| PPFIBP1 | 12 | 27523431 | 27695564 | diffuse large B-cell lymphoma | PPFIA binding protein 1 |
| PPFIBP1 | 12 | 27523431 | 27695564 | Ependymoma | PPFIA binding protein 1 |
| PPFIBP1 | 12 | 27523431 | 27695564 | gastric adenocarcinoma | PPFIA binding protein 1 |
| PPFIBP1 | 12 | 27523431 | 27695564 | head and neck squamous cell carcinoma | PPFIA binding protein 1 |
| PPFIBP1 | 12 | 27523431 | 27695564 | Hepatocellular Carcinoma | PPFIA binding protein 1 |
| PPFIBP1 | 12 | 27523431 | 27695564 | kidney neoplasm | PPFIA binding protein 1 |
| PPFIBP1 | 12 | 27523431 | 27695564 | large cell medulloblastoma | PPFIA binding protein 1 |
| PPFIBP1 | 12 | 27523431 | 27695564 | lobular breast carcinoma | PPFIA binding protein 1 |
| PPFIBP1 | 12 | 27523431 | 27695564 | Lung adenocarcinoma | PPFIA binding protein 1 |
| PPFIBP1 | 12 | 27523431 | 27695564 | Lung carcinoma | PPFIA binding protein 1 |
| PPFIBP1 | 12 | 27523431 | 27695564 | lymphoid neoplasm | PPFIA binding protein 1 |
| PPFIBP1 | 12 | 27523431 | 27695564 | Medulloblastoma | PPFIA binding protein 1 |
| PPFIBP1 | 12 | 27523431 | 27695564 | Melanoma | PPFIA binding protein 1 |
| PPFIBP1 | 12 | 27523431 | 27695564 | Multiple myeloma | PPFIA binding protein 1 |
| PPFIBP1 | 12 | 27523431 | 27695564 | nodular melanoma | PPFIA binding protein 1 |
| PPFIBP1 | 12 | 27523431 | 27695564 | non-small cell lung carcinoma | PPFIA binding protein 1 |
| PPFIBP1 | 12 | 27523431 | 27695564 | oral squamous cell carcinoma | PPFIA binding protein 1 |
| PPFIBP1 | 12 | 27523431 | 27695564 | OSTEOSARCOMA | PPFIA binding protein 1 |
| PPFIBP1 | 12 | 27523431 | 27695564 | pancreatic carcinoma | PPFIA binding protein 1 |
| PPFIBP1 | 12 | 27523431 | 27695564 | pancreatic ductal adenocarcinoma | PPFIA binding protein 1 |
| PPFIBP1 | 12 | 27523431 | 27695564 | pancreatic neuroendocrine tumor | PPFIA binding protein 1 |
| PPFIBP1 | 12 | 27523431 | 27695564 | prostate adenocarcinoma | PPFIA binding protein 1 |
| PPFIBP1 | 12 | 27523431 | 27695564 | prostate carcinoma | PPFIA binding protein 1 |
| PPFIBP1 | 12 | 27523431 | 27695564 | rectal adenocarcinoma | PPFIA binding protein 1 |
| PPFIBP1 | 12 | 27523431 | 27695564 | skin carcinoma | PPFIA binding protein 1 |
| PPFIBP1 | 12 | 27523431 | 27695564 | Small cell lung carcinoma | PPFIA binding protein 1 |
| PPFIBP1 | 12 | 27523431 | 27695564 | soft tissue sarcoma | PPFIA binding protein 1 |
| PPFIBP1 | 12 | 27523431 | 27695564 | squamous cell lung carcinoma | PPFIA binding protein 1 |
| PPFIBP1 | 12 | 27523431 | 27695564 | thyroid carcinoma | PPFIA binding protein 1 |
| PPFIBP1 | 12 | 27523431 | 27695564 | thyroid neoplasm | PPFIA binding protein 1 |
| LIN7A | 12 | 80792520 | 80937934 |  | lin-7 homolog A, crumbs cell polarity complex component |
| ADAMTS20 | 12 | 43353866 | 43552203 |  | ADAM metallopeptidase with thrombospondin type 1 motif 20 |
| SYT1 | 12 | 78863993 | 79452008 | Infantile hypotonia-oculomotor anomalies-hyperkinetic movements-developmental delay syndrome | synaptotagmin 1 |
| SYT1 | 12 | 78863993 | 79452008 | BAKER-GORDON SYNDROME | synaptotagmin 1 |
| SYT1 | 12 | 78863993 | 79452008 | Intellectual disability | synaptotagmin 1 |
| CACNA1C | 12 | 1970772 | 2697950 | Atypical Timothy syndrome | calcium voltage-gated channel subunit alpha1 C |
| CACNA1C | 12 | 1970772 | 2697950 | Brugada syndrome | calcium voltage-gated channel subunit alpha1 C |
| CACNA1C | 12 | 1970772 | 2697950 | Romano-Ward syndrome | calcium voltage-gated channel subunit alpha1 C |
| CACNA1C | 12 | 1970772 | 2697950 | Timothy syndrome type 1 | calcium voltage-gated channel subunit alpha1 C |
| CACNA1C | 12 | 1970772 | 2697950 | Timothy syndrome type 2 | calcium voltage-gated channel subunit alpha1 C |
| CACNA1C | 12 | 1970772 | 2697950 | BRUGADA SYNDROME 3 | calcium voltage-gated channel subunit alpha1 C |
| CACNA1C | 12 | 1970772 | 2697950 | Long QT syndrome 8 | calcium voltage-gated channel subunit alpha1 C |
| CACNA1C | 12 | 1970772 | 2697950 | Neurodevelopmental disorder with hypotonia language delay and skeletal defects with or without seizures | calcium voltage-gated channel subunit alpha1 C |
| CACNA1C | 12 | 1970772 | 2697950 | TIMOTHY SYNDROME | calcium voltage-gated channel subunit alpha1 C |
| CACNA1C | 12 | 1970772 | 2697950 | CACNA1C-related Timothy syndrome | calcium voltage-gated channel subunit alpha1 C |
| CACNA1C | 12 | 1970772 | 2697950 | Atypical Timothy syndrome | CACNA1C intronic transcript 2 |
| CACNA1C | 12 | 1970772 | 2697950 | Brugada syndrome | CACNA1C intronic transcript 2 |
| CACNA1C | 12 | 1970772 | 2697950 | Romano-Ward syndrome | CACNA1C intronic transcript 2 |
| CACNA1C | 12 | 1970772 | 2697950 | Timothy syndrome type 1 | CACNA1C intronic transcript 2 |
| CACNA1C | 12 | 1970772 | 2697950 | Timothy syndrome type 2 | CACNA1C intronic transcript 2 |
| CACNA1C | 12 | 1970772 | 2697950 | BRUGADA SYNDROME 3 | CACNA1C intronic transcript 2 |
| CACNA1C | 12 | 1970772 | 2697950 | Long QT syndrome 8 | CACNA1C intronic transcript 2 |
| CACNA1C | 12 | 1970772 | 2697950 | Neurodevelopmental disorder with hypotonia language delay and skeletal defects with or without seizures | CACNA1C intronic transcript 2 |
| CACNA1C | 12 | 1970772 | 2697950 | TIMOTHY SYNDROME | CACNA1C intronic transcript 2 |
| CACNA1C | 12 | 1970772 | 2697950 | CACNA1C-related Timothy syndrome | CACNA1C intronic transcript 2 |
| KRAS | 12 | 25205246 | 25250936 | CARDIOFACIOCUTANEOUS SYNDROME | KRAS proto-oncogene, GTPase |
| KRAS | 12 | 25205246 | 25250936 | Differentiated thyroid carcinoma | KRAS proto-oncogene, GTPase |
| KRAS | 12 | 25205246 | 25250936 | Encephalocraniocutaneous lipomatosis | KRAS proto-oncogene, GTPase |
| KRAS | 12 | 25205246 | 25250936 | Familial pancreatic carcinoma | KRAS proto-oncogene, GTPase |
| KRAS | 12 | 25205246 | 25250936 | Juvenile myelomonocytic leukemia | KRAS proto-oncogene, GTPase |
| KRAS | 12 | 25205246 | 25250936 | Linear nevus sebaceus syndrome | KRAS proto-oncogene, GTPase |
| KRAS | 12 | 25205246 | 25250936 | Lynch syndrome | KRAS proto-oncogene, GTPase |
| KRAS | 12 | 25205246 | 25250936 | Noonan Syndrome | KRAS proto-oncogene, GTPase |
| KRAS | 12 | 25205246 | 25250936 | Pilomyxoid astrocytoma | KRAS proto-oncogene, GTPase |
| KRAS | 12 | 25205246 | 25250936 | RAS-associated autoimmune leukoproliferative disease | KRAS proto-oncogene, GTPase |
| KRAS | 12 | 25205246 | 25250936 | Toriello-Lacassie-Droste syndrome | KRAS proto-oncogene, GTPase |
| KRAS | 12 | 25205246 | 25250936 | ARTERIOVENOUS MALFORMATIONS OF THE BRAIN | KRAS proto-oncogene, GTPase |
| KRAS | 12 | 25205246 | 25250936 | Bladder Cancer | KRAS proto-oncogene, GTPase |
| KRAS | 12 | 25205246 | 25250936 | Breast Cancer | KRAS proto-oncogene, GTPase |
| KRAS | 12 | 25205246 | 25250936 | CARDIOFACIOCUTANEOUS SYNDROME 2 | KRAS proto-oncogene, GTPase |
| KRAS | 12 | 25205246 | 25250936 | GASTRIC CANCER GASTRIC CANCER INTESTINAL INCLUDED | KRAS proto-oncogene, GTPase |
| KRAS | 12 | 25205246 | 25250936 | LEUKEMIA ACUTE MYELOID | KRAS proto-oncogene, GTPase |
| KRAS | 12 | 25205246 | 25250936 | LUNG CANCER ALVEOLAR CELL CARCINOMA INCLUDED | KRAS proto-oncogene, GTPase |
| KRAS | 12 | 25205246 | 25250936 | NOONAN SYNDROME 3 | KRAS proto-oncogene, GTPase |
| KRAS | 12 | 25205246 | 25250936 | Oculoectodermal syndrome | KRAS proto-oncogene, GTPase |
| KRAS | 12 | 25205246 | 25250936 | Pancreatic Cancer | KRAS proto-oncogene, GTPase |
| KRAS | 12 | 25205246 | 25250936 | RAS-associated autoimmune leukoproliferative disorder | KRAS proto-oncogene, GTPase |
| KRAS | 12 | 25205246 | 25250936 | SCHIMMELPENNING-FEUERSTEIN-MIMS SYNDROME | KRAS proto-oncogene, GTPase |
| KRAS | 12 | 25205246 | 25250936 | NOONAN SYNDROME TYPE 3 | KRAS proto-oncogene, GTPase |
| KRAS | 12 | 25205246 | 25250936 | Adenosquamous Carcinoma | KRAS proto-oncogene, GTPase |
| KRAS | 12 | 25205246 | 25250936 | Appendix Adenocarcinoma | KRAS proto-oncogene, GTPase |
| KRAS | 12 | 25205246 | 25250936 | B-cell acute lymphoblastic leukemia | KRAS proto-oncogene, GTPase |
| KRAS | 12 | 25205246 | 25250936 | Bladder Adenocarcinoma | KRAS proto-oncogene, GTPase |
| KRAS | 12 | 25205246 | 25250936 | Borderline Exocrine Pancreatic Neoplasm | KRAS proto-oncogene, GTPase |
| KRAS | 12 | 25205246 | 25250936 | Borderline Ovarian Brenner Tumor | KRAS proto-oncogene, GTPase |
| KRAS | 12 | 25205246 | 25250936 | Borderline Ovarian Endometrioid Tumor | KRAS proto-oncogene, GTPase |
| KRAS | 12 | 25205246 | 25250936 | Borderline Ovarian Mucinous Tumor | KRAS proto-oncogene, GTPase |
| KRAS | 12 | 25205246 | 25250936 | Borderline Ovarian Serous Tumor | KRAS proto-oncogene, GTPase |
| KRAS | 12 | 25205246 | 25250936 | Borderline Ovarian Surface Epithelial-Stromal Tumor | KRAS proto-oncogene, GTPase |
| KRAS | 12 | 25205246 | 25250936 | Breast Carcinoma by Gene Expression Profile | KRAS proto-oncogene, GTPase |
| KRAS | 12 | 25205246 | 25250936 | Burkitts lymphoma | KRAS proto-oncogene, GTPase |
| KRAS | 12 | 25205246 | 25250936 | Chronic neutrophilic leukemia | KRAS proto-oncogene, GTPase |
| KRAS | 12 | 25205246 | 25250936 | Colorectal Neuroendocrine Tumor G1 | KRAS proto-oncogene, GTPase |
| KRAS | 12 | 25205246 | 25250936 | Colorectal Sessile Serrated Adenoma/Polyp | KRAS proto-oncogene, GTPase |
| KRAS | 12 | 25205246 | 25250936 | Cutaneous T-cell lymphoma | KRAS proto-oncogene, GTPase |
| KRAS | 12 | 25205246 | 25250936 | Digestive System Adenoma | KRAS proto-oncogene, GTPase |
| KRAS | 12 | 25205246 | 25250936 | Digestive System Carcinoma | KRAS proto-oncogene, GTPase |
| KRAS | 12 | 25205246 | 25250936 | Duodenal Adenocarcinoma | KRAS proto-oncogene, GTPase |
| KRAS | 12 | 25205246 | 25250936 | Duodenal Villous Adenoma | KRAS proto-oncogene, GTPase |
| KRAS | 12 | 25205246 | 25250936 | Dysplasia in Ulcerative Colitis | KRAS proto-oncogene, GTPase |
| KRAS | 12 | 25205246 | 25250936 | Endometrial Endometrioid Adenocarcinoma | KRAS proto-oncogene, GTPase |
| KRAS | 12 | 25205246 | 25250936 | Endometrial Mucinous Adenocarcinoma | KRAS proto-oncogene, GTPase |
| KRAS | 12 | 25205246 | 25250936 | Endometrial Polyp | KRAS proto-oncogene, GTPase |
| KRAS | 12 | 25205246 | 25250936 | Endometrial Squamous Cell Carcinoma | KRAS proto-oncogene, GTPase |
| KRAS | 12 | 25205246 | 25250936 | Extrahepatic Bile Duct Adenosquamous Carcinoma | KRAS proto-oncogene, GTPase |
| KRAS | 12 | 25205246 | 25250936 | Extrahepatic Bile Duct Squamous Cell Carcinoma | KRAS proto-oncogene, GTPase |
| KRAS | 12 | 25205246 | 25250936 | Gallbladder Small Cell Neuroendocrine Carcinoma | KRAS proto-oncogene, GTPase |
| KRAS | 12 | 25205246 | 25250936 | Gastric Adenoma | KRAS proto-oncogene, GTPase |
| KRAS | 12 | 25205246 | 25250936 | Gastrointestinal Hamartoma | KRAS proto-oncogene, GTPase |
| KRAS | 12 | 25205246 | 25250936 | Granulocytic Sarcoma | KRAS proto-oncogene, GTPase |
| KRAS | 12 | 25205246 | 25250936 | Hepatobiliary Neoplasm | KRAS proto-oncogene, GTPase |
| KRAS | 12 | 25205246 | 25250936 | Histiocytic and Dendritic Cell Neoplasm | KRAS proto-oncogene, GTPase |
| KRAS | 12 | 25205246 | 25250936 | Infiltrating Bladder Urothelial Carcinoma Sarcomatoid Variant | KRAS proto-oncogene, GTPase |
| KRAS | 12 | 25205246 | 25250936 | Invasive Breast Carcinoma | KRAS proto-oncogene, GTPase |
| KRAS | 12 | 25205246 | 25250936 | Juvenile Polyp | KRAS proto-oncogene, GTPase |
| KRAS | 12 | 25205246 | 25250936 | Malignant Ovarian Mixed Epithelial Tumor | KRAS proto-oncogene, GTPase |
| KRAS | 12 | 25205246 | 25250936 | Malignant Pancreatic Neoplasm | KRAS proto-oncogene, GTPase |
| KRAS | 12 | 25205246 | 25250936 | MANTLE CELL LYMPHOMA | KRAS proto-oncogene, GTPase |
| KRAS | 12 | 25205246 | 25250936 | Merkel cell skin cancer | KRAS proto-oncogene, GTPase |
| KRAS | 12 | 25205246 | 25250936 | ND | KRAS proto-oncogene, GTPase |
| KRAS | 12 | 25205246 | 25250936 | Non-Neoplastic Bile Duct Disorder | KRAS proto-oncogene, GTPase |
| KRAS | 12 | 25205246 | 25250936 | Ovarian Carcinosarcoma | KRAS proto-oncogene, GTPase |
| KRAS | 12 | 25205246 | 25250936 | Ovarian Dysgerminoma | KRAS proto-oncogene, GTPase |
| KRAS | 12 | 25205246 | 25250936 | Ovarian Endometrioid Adenocarcinoma | KRAS proto-oncogene, GTPase |
| KRAS | 12 | 25205246 | 25250936 | Ovarian Endometrioid Adenocarcinoma with Squamous Differentiation | KRAS proto-oncogene, GTPase |
| KRAS | 12 | 25205246 | 25250936 | Ovarian Serous Adenofibroma | KRAS proto-oncogene, GTPase |
| KRAS | 12 | 25205246 | 25250936 | Ovarian Sertoli-Leydig Cell Tumor | KRAS proto-oncogene, GTPase |
| KRAS | 12 | 25205246 | 25250936 | Ovarian Yolk Sac Tumor | KRAS proto-oncogene, GTPase |
| KRAS | 12 | 25205246 | 25250936 | Pancreatic Acinar Cell Carcinoma | KRAS proto-oncogene, GTPase |
| KRAS | 12 | 25205246 | 25250936 | Pancreatic Large Cell Neuroendocrine Carcinoma | KRAS proto-oncogene, GTPase |
| KRAS | 12 | 25205246 | 25250936 | Pleural Biphasic Mesothelioma | KRAS proto-oncogene, GTPase |
| KRAS | 12 | 25205246 | 25250936 | Pleural Epithelioid Mesothelioma | KRAS proto-oncogene, GTPase |
| KRAS | 12 | 25205246 | 25250936 | Pyloric Gland Adenoma | KRAS proto-oncogene, GTPase |
| KRAS | 12 | 25205246 | 25250936 | Rectal Villous Adenoma | KRAS proto-oncogene, GTPase |
| KRAS | 12 | 25205246 | 25250936 | Salivary Gland Carcinoma ex Pleomorphic Adenoma | KRAS proto-oncogene, GTPase |
| KRAS | 12 | 25205246 | 25250936 | Sarcomatoid Carcinoma | KRAS proto-oncogene, GTPase |
| KRAS | 12 | 25205246 | 25250936 | Signet Ring Cell Gastric Adenocarcinoma | KRAS proto-oncogene, GTPase |
| KRAS | 12 | 25205246 | 25250936 | Simple Endometrial Hyperplasia | KRAS proto-oncogene, GTPase |
| KRAS | 12 | 25205246 | 25250936 | Sinonasal Undifferentiated Carcinoma | KRAS proto-oncogene, GTPase |
| KRAS | 12 | 25205246 | 25250936 | Small Intestinal Tubulovillous Adenoma | KRAS proto-oncogene, GTPase |
| KRAS | 12 | 25205246 | 25250936 | Submandibular Gland Adenoid Cystic Carcinoma | KRAS proto-oncogene, GTPase |
| KRAS | 12 | 25205246 | 25250936 | Syringocystadenoma Papilliferum | KRAS proto-oncogene, GTPase |
| KRAS | 12 | 25205246 | 25250936 | Systemic Mastocytosis with Associated Clonal Hematological non-Mast-Cell Lineage Disease | KRAS proto-oncogene, GTPase |
| KRAS | 12 | 25205246 | 25250936 | T-cell acute lymphoblastic leukemia | KRAS proto-oncogene, GTPase |
| KRAS | 12 | 25205246 | 25250936 | Testicular Teratoma | KRAS proto-oncogene, GTPase |
| KRAS | 12 | 25205246 | 25250936 | Testicular Yolk Sac Tumor | KRAS proto-oncogene, GTPase |
| KRAS | 12 | 25205246 | 25250936 | Thymic Carcinoma | KRAS proto-oncogene, GTPase |
| KRAS | 12 | 25205246 | 25250936 | Thymic Squamous Cell Carcinoma | KRAS proto-oncogene, GTPase |
| KRAS | 12 | 25205246 | 25250936 | Thymoma | KRAS proto-oncogene, GTPase |
| KRAS | 12 | 25205246 | 25250936 | Thyroid Gland Diffuse Large B-Cell Lymphoma | KRAS proto-oncogene, GTPase |
| KRAS | 12 | 25205246 | 25250936 | Thyroid Gland Undifferentiated (Anaplastic) Carcinoma | KRAS proto-oncogene, GTPase |
| KRAS | 12 | 25205246 | 25250936 | Tracheal Carcinoma | KRAS proto-oncogene, GTPase |
| KRAS | 12 | 25205246 | 25250936 | Transitional Meningioma | KRAS proto-oncogene, GTPase |
| KRAS | 12 | 25205246 | 25250936 | Undifferentiated Gallbladder Carcinoma | KRAS proto-oncogene, GTPase |
| KRAS | 12 | 25205246 | 25250936 | Undifferentiated Pancreatic Carcinoma | KRAS proto-oncogene, GTPase |
| KRAS | 12 | 25205246 | 25250936 | Vulvar Squamous Cell Carcinoma | KRAS proto-oncogene, GTPase |
| KRAS | 12 | 25205246 | 25250936 | Acute lymphoblastic leukemia | KRAS proto-oncogene, GTPase |
| KRAS | 12 | 25205246 | 25250936 | Acute myeloid leukemia | KRAS proto-oncogene, GTPase |
| KRAS | 12 | 25205246 | 25250936 | acute pancreatitis | KRAS proto-oncogene, GTPase |
| KRAS | 12 | 25205246 | 25250936 | Adenoid cystic carcinoma | KRAS proto-oncogene, GTPase |
| KRAS | 12 | 25205246 | 25250936 | adenosquamous lung carcinoma | KRAS proto-oncogene, GTPase |
| KRAS | 12 | 25205246 | 25250936 | Anaplastic astrocytoma | KRAS proto-oncogene, GTPase |
| KRAS | 12 | 25205246 | 25250936 | angiosarcoma | KRAS proto-oncogene, GTPase |
| KRAS | 12 | 25205246 | 25250936 | Astrocytoma | KRAS proto-oncogene, GTPase |
| KRAS | 12 | 25205246 | 25250936 | Basal cell carcinoma | KRAS proto-oncogene, GTPase |
| KRAS | 12 | 25205246 | 25250936 | benign prostatic hyperplasia | KRAS proto-oncogene, GTPase |
| KRAS | 12 | 25205246 | 25250936 | bile duct carcinoma | KRAS proto-oncogene, GTPase |
| KRAS | 12 | 25205246 | 25250936 | bladder transitional cell carcinoma | KRAS proto-oncogene, GTPase |
| KRAS | 12 | 25205246 | 25250936 | brain glioblastoma | KRAS proto-oncogene, GTPase |
| KRAS | 12 | 25205246 | 25250936 | Breast carcinoma | KRAS proto-oncogene, GTPase |
| KRAS | 12 | 25205246 | 25250936 | breast ductal adenocarcinoma | KRAS proto-oncogene, GTPase |
| KRAS | 12 | 25205246 | 25250936 | carcinoid tumor | KRAS proto-oncogene, GTPase |
| KRAS | 12 | 25205246 | 25250936 | Carcinoma | KRAS proto-oncogene, GTPase |
| KRAS | 12 | 25205246 | 25250936 | cecum adenocarcinoma | KRAS proto-oncogene, GTPase |
| KRAS | 12 | 25205246 | 25250936 | cervical adenocarcinoma | KRAS proto-oncogene, GTPase |
| KRAS | 12 | 25205246 | 25250936 | cervical carcinoma | KRAS proto-oncogene, GTPase |
| KRAS | 12 | 25205246 | 25250936 | cervical squamous cell carcinoma | KRAS proto-oncogene, GTPase |
| KRAS | 12 | 25205246 | 25250936 | Chronic lymphocytic leukemia | KRAS proto-oncogene, GTPase |
| KRAS | 12 | 25205246 | 25250936 | Chronic myelogenous leukemia | KRAS proto-oncogene, GTPase |
| KRAS | 12 | 25205246 | 25250936 | Chronic myelomonocytic leukemia | KRAS proto-oncogene, GTPase |
| KRAS | 12 | 25205246 | 25250936 | chronic pancreatitis | KRAS proto-oncogene, GTPase |
| KRAS | 12 | 25205246 | 25250936 | clear cell adenocarcinoma | KRAS proto-oncogene, GTPase |
| KRAS | 12 | 25205246 | 25250936 | Clear cell renal carcinoma | KRAS proto-oncogene, GTPase |
| KRAS | 12 | 25205246 | 25250936 | colon adenocarcinoma | KRAS proto-oncogene, GTPase |
| KRAS | 12 | 25205246 | 25250936 | colon carcinoma | KRAS proto-oncogene, GTPase |
| KRAS | 12 | 25205246 | 25250936 | colonic neoplasm | KRAS proto-oncogene, GTPase |
| KRAS | 12 | 25205246 | 25250936 | colorectal adenocarcinoma | KRAS proto-oncogene, GTPase |
| KRAS | 12 | 25205246 | 25250936 | Colorectal adenoma | KRAS proto-oncogene, GTPase |
| KRAS | 12 | 25205246 | 25250936 | colorectal carcinoma | KRAS proto-oncogene, GTPase |
| KRAS | 12 | 25205246 | 25250936 | colorectal neoplasm | KRAS proto-oncogene, GTPase |
| KRAS | 12 | 25205246 | 25250936 | diffuse gastric adenocarcinoma | KRAS proto-oncogene, GTPase |
| KRAS | 12 | 25205246 | 25250936 | diffuse large B-cell lymphoma | KRAS proto-oncogene, GTPase |
| KRAS | 12 | 25205246 | 25250936 | endodermal sinus tumor | KRAS proto-oncogene, GTPase |
| KRAS | 12 | 25205246 | 25250936 | Endometrial carcinoma | KRAS proto-oncogene, GTPase |
| KRAS | 12 | 25205246 | 25250936 | Endometrial stromal sarcoma | KRAS proto-oncogene, GTPase |
| KRAS | 12 | 25205246 | 25250936 | endometrium adenocarcinoma | KRAS proto-oncogene, GTPase |
| KRAS | 12 | 25205246 | 25250936 | gastric adenocarcinoma | KRAS proto-oncogene, GTPase |
| KRAS | 12 | 25205246 | 25250936 | Gastritis | KRAS proto-oncogene, GTPase |
| KRAS | 12 | 25205246 | 25250936 | hairy cell leukemia | KRAS proto-oncogene, GTPase |
| KRAS | 12 | 25205246 | 25250936 | Hamartoma | KRAS proto-oncogene, GTPase |
| KRAS | 12 | 25205246 | 25250936 | head and neck squamous cell carcinoma | KRAS proto-oncogene, GTPase |
| KRAS | 12 | 25205246 | 25250936 | Hepatocellular Carcinoma | KRAS proto-oncogene, GTPase |
| KRAS | 12 | 25205246 | 25250936 | hyperplasia | KRAS proto-oncogene, GTPase |
| KRAS | 12 | 25205246 | 25250936 | kidney neoplasm | KRAS proto-oncogene, GTPase |
| KRAS | 12 | 25205246 | 25250936 | large cell lung carcinoma | KRAS proto-oncogene, GTPase |
| KRAS | 12 | 25205246 | 25250936 | leiomyosarcoma | KRAS proto-oncogene, GTPase |
| KRAS | 12 | 25205246 | 25250936 | lobular breast carcinoma | KRAS proto-oncogene, GTPase |
| KRAS | 12 | 25205246 | 25250936 | Lung adenocarcinoma | KRAS proto-oncogene, GTPase |
| KRAS | 12 | 25205246 | 25250936 | Lung carcinoma | KRAS proto-oncogene, GTPase |
| KRAS | 12 | 25205246 | 25250936 | lymphoid neoplasm | KRAS proto-oncogene, GTPase |
| KRAS | 12 | 25205246 | 25250936 | Malignant peripheral nerve sheath tumor | KRAS proto-oncogene, GTPase |
| KRAS | 12 | 25205246 | 25250936 | Melanoma | KRAS proto-oncogene, GTPase |
| KRAS | 12 | 25205246 | 25250936 | mucinous carcinoma | KRAS proto-oncogene, GTPase |
| KRAS | 12 | 25205246 | 25250936 | Multiple myeloma | KRAS proto-oncogene, GTPase |
| KRAS | 12 | 25205246 | 25250936 | myeloid neoplasm | KRAS proto-oncogene, GTPase |
| KRAS | 12 | 25205246 | 25250936 | Myeloproliferative disorder | KRAS proto-oncogene, GTPase |
| KRAS | 12 | 25205246 | 25250936 | neoplasm of mature B-cells | KRAS proto-oncogene, GTPase |
| KRAS | 12 | 25205246 | 25250936 | nodular melanoma | KRAS proto-oncogene, GTPase |
| KRAS | 12 | 25205246 | 25250936 | non-small cell lung carcinoma | KRAS proto-oncogene, GTPase |
| KRAS | 12 | 25205246 | 25250936 | Oligoastrocytoma | KRAS proto-oncogene, GTPase |
| KRAS | 12 | 25205246 | 25250936 | oral squamous cell carcinoma | KRAS proto-oncogene, GTPase |
| KRAS | 12 | 25205246 | 25250936 | OSTEOSARCOMA | KRAS proto-oncogene, GTPase |
| KRAS | 12 | 25205246 | 25250936 | ovarian adenocarcinoma | KRAS proto-oncogene, GTPase |
| KRAS | 12 | 25205246 | 25250936 | ovarian carcinoma | KRAS proto-oncogene, GTPase |
| KRAS | 12 | 25205246 | 25250936 | ovarian clear cell adenocarcinoma | KRAS proto-oncogene, GTPase |
| KRAS | 12 | 25205246 | 25250936 | ovarian mucinous adenocarcinoma | KRAS proto-oncogene, GTPase |
| KRAS | 12 | 25205246 | 25250936 | Ovarian neoplasm | KRAS proto-oncogene, GTPase |
| KRAS | 12 | 25205246 | 25250936 | ovarian teratoma | KRAS proto-oncogene, GTPase |
| KRAS | 12 | 25205246 | 25250936 | Pancreatic adenocarcinoma | KRAS proto-oncogene, GTPase |
| KRAS | 12 | 25205246 | 25250936 | pancreatic carcinoma | KRAS proto-oncogene, GTPase |
| KRAS | 12 | 25205246 | 25250936 | pancreatic ductal adenocarcinoma | KRAS proto-oncogene, GTPase |
| KRAS | 12 | 25205246 | 25250936 | pancreatic neoplasm | KRAS proto-oncogene, GTPase |
| KRAS | 12 | 25205246 | 25250936 | pancreatic neuroendocrine tumor | KRAS proto-oncogene, GTPase |
| KRAS | 12 | 25205246 | 25250936 | pharyngeal squamous cell carcinoma | KRAS proto-oncogene, GTPase |
| KRAS | 12 | 25205246 | 25250936 | plasmacytoma | KRAS proto-oncogene, GTPase |
| KRAS | 12 | 25205246 | 25250936 | POLYCYTHEMIA VERA | KRAS proto-oncogene, GTPase |
| KRAS | 12 | 25205246 | 25250936 | prostate adenocarcinoma | KRAS proto-oncogene, GTPase |
| KRAS | 12 | 25205246 | 25250936 | prostate carcinoma | KRAS proto-oncogene, GTPase |
| KRAS | 12 | 25205246 | 25250936 | pseudomyxoma peritonei | KRAS proto-oncogene, GTPase |
| KRAS | 12 | 25205246 | 25250936 | pulmonary blastoma | KRAS proto-oncogene, GTPase |
| KRAS | 12 | 25205246 | 25250936 | rectal adenocarcinoma | KRAS proto-oncogene, GTPase |
| KRAS | 12 | 25205246 | 25250936 | renal carcinoma | KRAS proto-oncogene, GTPase |
| KRAS | 12 | 25205246 | 25250936 | Renal cell carcinoma | KRAS proto-oncogene, GTPase |
| KRAS | 12 | 25205246 | 25250936 | Rhabdomyosarcoma | KRAS proto-oncogene, GTPase |
| KRAS | 12 | 25205246 | 25250936 | sebaceous adenocarcinoma | KRAS proto-oncogene, GTPase |
| KRAS | 12 | 25205246 | 25250936 | seborrheic keratosis | KRAS proto-oncogene, GTPase |
| KRAS | 12 | 25205246 | 25250936 | skin carcinoma | KRAS proto-oncogene, GTPase |
| KRAS | 12 | 25205246 | 25250936 | Small cell lung carcinoma | KRAS proto-oncogene, GTPase |
| KRAS | 12 | 25205246 | 25250936 | soft tissue sarcoma | KRAS proto-oncogene, GTPase |
| KRAS | 12 | 25205246 | 25250936 | Squamous cell carcinoma | KRAS proto-oncogene, GTPase |
| KRAS | 12 | 25205246 | 25250936 | squamous cell lung carcinoma | KRAS proto-oncogene, GTPase |
| KRAS | 12 | 25205246 | 25250936 | Synovial sarcoma | KRAS proto-oncogene, GTPase |
| KRAS | 12 | 25205246 | 25250936 | testicular mixed germ cell tumor | KRAS proto-oncogene, GTPase |
| KRAS | 12 | 25205246 | 25250936 | testicular neoplasm | KRAS proto-oncogene, GTPase |
| KRAS | 12 | 25205246 | 25250936 | testicular seminoma | KRAS proto-oncogene, GTPase |
| KRAS | 12 | 25205246 | 25250936 | thyroid carcinoma | KRAS proto-oncogene, GTPase |
| KRAS | 12 | 25205246 | 25250936 | thyroid neoplasm | KRAS proto-oncogene, GTPase |
| KRAS | 12 | 25205246 | 25250936 | Ulcerative colitis | KRAS proto-oncogene, GTPase |
| KRAS | 12 | 25205246 | 25250936 | undifferentiated pleomorphic sarcoma | KRAS proto-oncogene, GTPase |
| KRAS | 12 | 25205246 | 25250936 | urothelial carcinoma | KRAS proto-oncogene, GTPase |
| COL4A2 | 13 | 110305812 | 110513209 | Familial porencephaly | collagen type IV alpha 2 chain |
| COL4A2 | 13 | 110305812 | 110513209 | BRAIN SMALL VESSEL DISEASE 2 | collagen type IV alpha 2 chain |
| COL4A2 | 13 | 110305812 | 110513209 | HEMORRHAGE INTRACEREBRAL SUSCEPTIBILITY TO | collagen type IV alpha 2 chain |
| COL4A2 | 13 | 110305812 | 110513209 | Porencephaly 2 | collagen type IV alpha 2 chain |
| COL4A1 | 13 | 110148963 | 110307157 | COL4A1-related familial vascular leukoencephalopathy | collagen type IV alpha 1 chain |
| COL4A1 | 13 | 110148963 | 110307157 | Familial porencephaly | collagen type IV alpha 1 chain |
| COL4A1 | 13 | 110148963 | 110307157 | Familial schizencephaly | collagen type IV alpha 1 chain |
| COL4A1 | 13 | 110148963 | 110307157 | HANAC syndrome | collagen type IV alpha 1 chain |
| COL4A1 | 13 | 110148963 | 110307157 | Pontine autosomal dominant microangiopathy with leukoencephalopathy | collagen type IV alpha 1 chain |
| COL4A1 | 13 | 110148963 | 110307157 | Retinal arterial tortuosity | collagen type IV alpha 1 chain |
| COL4A1 | 13 | 110148963 | 110307157 | Walker-Warburg syndrome | collagen type IV alpha 1 chain |
| COL4A1 | 13 | 110148963 | 110307157 | Angiopathy hereditary with nephropathy aneurysms and muscle cramps | collagen type IV alpha 1 chain |
| COL4A1 | 13 | 110148963 | 110307157 | BRAIN SMALL VESSEL DISEASE 1 WITH OR WITHOUT OCULAR ANOMALIES | collagen type IV alpha 1 chain |
| COL4A1 | 13 | 110148963 | 110307157 | HEMORRHAGE INTRACEREBRAL SUSCEPTIBILITY TO | collagen type IV alpha 1 chain |
| COL4A1 | 13 | 110148963 | 110307157 | MICROANGIOPATHY AND LEUKOENCEPHALOPATHY PONTINE AUTOSOMAL DOMINANT | collagen type IV alpha 1 chain |
| COL4A1 | 13 | 110148963 | 110307157 | Retinal arteries tortuosity of | collagen type IV alpha 1 chain |
| COL4A1 | 13 | 110148963 | 110307157 | Brain small vessel disease with or without ocular anomalies | collagen type IV alpha 1 chain |
| COL4A1 | 13 | 110148963 | 110307157 | PORENCEPHALY 1 | collagen type IV alpha 1 chain |
| NBEA | 13 | 34942270 | 35673022 | Autosomal dominant non-syndromic intellectual disability | neurobeachin |
| NBEA | 13 | 34942270 | 35673022 | NEURODEVELOPMENTAL DISORDER WITH OR WITHOUT EARLY-ONSET GENERALIZED EPILEPSY | neurobeachin |
| NBEA | 13 | 34942270 | 35673022 | NBEA Neurodevelopment disorder with seizures | neurobeachin |
| NBEA | 13 | 34942270 | 35673022 | Atypical Meningioma | neurobeachin |
| NBEA | 13 | 34942270 | 35673022 | B-cell acute lymphoblastic leukemia | neurobeachin |
| NBEA | 13 | 34942270 | 35673022 | Bladder Small Cell Neuroendocrine Carcinoma | neurobeachin |
| NBEA | 13 | 34942270 | 35673022 | Brain Stem Glioblastoma | neurobeachin |
| NBEA | 13 | 34942270 | 35673022 | Breast Carcinoma by Gene Expression Profile | neurobeachin |
| NBEA | 13 | 34942270 | 35673022 | Burkitts lymphoma | neurobeachin |
| NBEA | 13 | 34942270 | 35673022 | Chordoid Meningioma | neurobeachin |
| NBEA | 13 | 34942270 | 35673022 | Duodenal Adenocarcinoma | neurobeachin |
| NBEA | 13 | 34942270 | 35673022 | Endometrial Endometrioid Adenocarcinoma | neurobeachin |
| NBEA | 13 | 34942270 | 35673022 | Gallbladder Small Cell Neuroendocrine Carcinoma | neurobeachin |
| NBEA | 13 | 34942270 | 35673022 | Gastric Adenoma | neurobeachin |
| NBEA | 13 | 34942270 | 35673022 | Hepatobiliary Neoplasm | neurobeachin |
| NBEA | 13 | 34942270 | 35673022 | Hodgkins lymphoma | neurobeachin |
| NBEA | 13 | 34942270 | 35673022 | MANTLE CELL LYMPHOMA | neurobeachin |
| NBEA | 13 | 34942270 | 35673022 | Merkel cell skin cancer | neurobeachin |
| NBEA | 13 | 34942270 | 35673022 | ND | neurobeachin |
| NBEA | 13 | 34942270 | 35673022 | Ovarian Endometrioid Adenocarcinoma with Squamous Differentiation | neurobeachin |
| NBEA | 13 | 34942270 | 35673022 | Pancreatic Acinar Cell Carcinoma | neurobeachin |
| NBEA | 13 | 34942270 | 35673022 | Placental Choriocarcinoma | neurobeachin |
| NBEA | 13 | 34942270 | 35673022 | Pleural Epithelioid Mesothelioma | neurobeachin |
| NBEA | 13 | 34942270 | 35673022 | Secretory Meningioma | neurobeachin |
| NBEA | 13 | 34942270 | 35673022 | T-cell acute lymphoblastic leukemia | neurobeachin |
| NBEA | 13 | 34942270 | 35673022 | Transitional Meningioma | neurobeachin |
| NBEA | 13 | 34942270 | 35673022 | Acute lymphoblastic leukemia | neurobeachin |
| NBEA | 13 | 34942270 | 35673022 | Acute myeloid leukemia | neurobeachin |
| NBEA | 13 | 34942270 | 35673022 | adenosquamous lung carcinoma | neurobeachin |
| NBEA | 13 | 34942270 | 35673022 | adrenocortical adenoma | neurobeachin |
| NBEA | 13 | 34942270 | 35673022 | Anaplastic astrocytoma | neurobeachin |
| NBEA | 13 | 34942270 | 35673022 | angiosarcoma | neurobeachin |
| NBEA | 13 | 34942270 | 35673022 | bile duct carcinoma | neurobeachin |
| NBEA | 13 | 34942270 | 35673022 | bladder transitional cell carcinoma | neurobeachin |
| NBEA | 13 | 34942270 | 35673022 | brain glioblastoma | neurobeachin |
| NBEA | 13 | 34942270 | 35673022 | Breast carcinoma | neurobeachin |
| NBEA | 13 | 34942270 | 35673022 | breast ductal adenocarcinoma | neurobeachin |
| NBEA | 13 | 34942270 | 35673022 | cecum adenocarcinoma | neurobeachin |
| NBEA | 13 | 34942270 | 35673022 | cervical squamous cell carcinoma | neurobeachin |
| NBEA | 13 | 34942270 | 35673022 | Chronic lymphocytic leukemia | neurobeachin |
| NBEA | 13 | 34942270 | 35673022 | Clear cell renal carcinoma | neurobeachin |
| NBEA | 13 | 34942270 | 35673022 | colon adenocarcinoma | neurobeachin |
| NBEA | 13 | 34942270 | 35673022 | colon carcinoma | neurobeachin |
| NBEA | 13 | 34942270 | 35673022 | colonic neoplasm | neurobeachin |
| NBEA | 13 | 34942270 | 35673022 | colorectal adenocarcinoma | neurobeachin |
| NBEA | 13 | 34942270 | 35673022 | diffuse gastric adenocarcinoma | neurobeachin |
| NBEA | 13 | 34942270 | 35673022 | diffuse large B-cell lymphoma | neurobeachin |
| NBEA | 13 | 34942270 | 35673022 | Ependymoma | neurobeachin |
| NBEA | 13 | 34942270 | 35673022 | gastric adenocarcinoma | neurobeachin |
| NBEA | 13 | 34942270 | 35673022 | hairy cell leukemia | neurobeachin |
| NBEA | 13 | 34942270 | 35673022 | head and neck squamous cell carcinoma | neurobeachin |
| NBEA | 13 | 34942270 | 35673022 | Hepatocellular Carcinoma | neurobeachin |
| NBEA | 13 | 34942270 | 35673022 | kidney neoplasm | neurobeachin |
| NBEA | 13 | 34942270 | 35673022 | large cell lung carcinoma | neurobeachin |
| NBEA | 13 | 34942270 | 35673022 | large cell medulloblastoma | neurobeachin |
| NBEA | 13 | 34942270 | 35673022 | lobular breast carcinoma | neurobeachin |
| NBEA | 13 | 34942270 | 35673022 | Lung adenocarcinoma | neurobeachin |
| NBEA | 13 | 34942270 | 35673022 | Lung carcinoma | neurobeachin |
| NBEA | 13 | 34942270 | 35673022 | lymphoid neoplasm | neurobeachin |
| NBEA | 13 | 34942270 | 35673022 | Medulloblastoma | neurobeachin |
| NBEA | 13 | 34942270 | 35673022 | Melanoma | neurobeachin |
| NBEA | 13 | 34942270 | 35673022 | Multiple myeloma | neurobeachin |
| NBEA | 13 | 34942270 | 35673022 | Neoplasm | neurobeachin |
| NBEA | 13 | 34942270 | 35673022 | neoplasm of mature B-cells | neurobeachin |
| NBEA | 13 | 34942270 | 35673022 | nodular melanoma | neurobeachin |
| NBEA | 13 | 34942270 | 35673022 | non-small cell lung carcinoma | neurobeachin |
| NBEA | 13 | 34942270 | 35673022 | oral squamous cell carcinoma | neurobeachin |
| NBEA | 13 | 34942270 | 35673022 | OSTEOSARCOMA | neurobeachin |
| NBEA | 13 | 34942270 | 35673022 | ovarian mucinous adenocarcinoma | neurobeachin |
| NBEA | 13 | 34942270 | 35673022 | pancreatic carcinoma | neurobeachin |
| NBEA | 13 | 34942270 | 35673022 | pancreatic ductal adenocarcinoma | neurobeachin |
| NBEA | 13 | 34942270 | 35673022 | pancreatic neuroendocrine tumor | neurobeachin |
| NBEA | 13 | 34942270 | 35673022 | pharyngeal squamous cell carcinoma | neurobeachin |
| NBEA | 13 | 34942270 | 35673022 | prostate adenocarcinoma | neurobeachin |
| NBEA | 13 | 34942270 | 35673022 | prostate carcinoma | neurobeachin |
| NBEA | 13 | 34942270 | 35673022 | rectal adenocarcinoma | neurobeachin |
| NBEA | 13 | 34942270 | 35673022 | Rhabdomyosarcoma | neurobeachin |
| NBEA | 13 | 34942270 | 35673022 | skin carcinoma | neurobeachin |
| NBEA | 13 | 34942270 | 35673022 | Small cell lung carcinoma | neurobeachin |
| NBEA | 13 | 34942270 | 35673022 | soft tissue sarcoma | neurobeachin |
| NBEA | 13 | 34942270 | 35673022 | squamous cell lung carcinoma | neurobeachin |
| NBEA | 13 | 34942270 | 35673022 | testicular seminoma | neurobeachin |
| NBEA | 13 | 34942270 | 35673022 | thyroid carcinoma | neurobeachin |
| NBEA | 13 | 34942270 | 35673022 | urothelial carcinoma | neurobeachin |
| NRXN3 | 14 | 78170373 | 79868291 | Autism | neurexin 3 |
| ACTN1 | 14 | 68874128 | 68979440 | Autosomal dominant macrothrombocytopenia | actinin alpha 1 |
| ACTN1 | 14 | 68874128 | 68979440 | BLEEDING DISORDER PLATELET-TYPE 15 | actinin alpha 1 |
| GALNT16 | 14 | 69259277 | 69357033 |  | polypeptide N-acetylgalactosaminyltransferase 16 |
| ADAMTS7 | 15 | 78759206 | 78811464 |  | ADAM metallopeptidase with thrombospondin type 1 motif 7 |
| HOMER2 | 15 | 82836946 | 82986153 | Autosomal dominant non-syndromic sensorineural deafness type DFNA | homer scaffold protein 2 |
| HOMER2 | 15 | 82836946 | 82986153 | Deafness autosomal dominant 68 | homer scaffold protein 2 |
| CHRNA7 | 15 | 31923438 | 32173018 | 15q13.3 microdeletion syndrome | cholinergic receptor nicotinic alpha 7 subunit |
| CHRNA7 | 15 | 31923438 | 32173018 | CHROMOSOME 15q13.3 DELETION SYNDROME | cholinergic receptor nicotinic alpha 7 subunit |
| ADAMTS17 | 15 | 99971437 | 100342005 | Ichthyosis-short stature-brachydactyly-microspherophakia syndrome | ADAM metallopeptidase with thrombospondin type 1 motif 17 |
| ADAMTS17 | 15 | 99971437 | 100342005 | WEILL-MARCHESANI SYNDROME 4 | ADAM metallopeptidase with thrombospondin type 1 motif 17 |
| ADAMTS17 | 15 | 99971437 | 100342005 | Weill-Marchesani-like syndrome | ADAM metallopeptidase with thrombospondin type 1 motif 17 |
| APBA2 | 15 | 28884483 | 29118315 |  | amyloid beta precursor protein binding family A member 2 |
| THSD4 | 15 | 71096952 | 71783383 | AORTIC ANEURYSM FAMILIAL THORACIC 12 | thrombospondin type 1 domain containing 4 |
| ADAMTS18 | 16 | 77247813 | 77435034 | Microcornea-myopic chorioretinal atrophy-telecanthus syndrome | ADAM metallopeptidase with thrombospondin type 1 motif 18 |
| ADAMTS18 | 16 | 77247813 | 77435034 | Microcornea myopic chorioretinal atrophy and telecanthus | ADAM metallopeptidase with thrombospondin type 1 motif 18 |
| GRIN2A | 16 | 9753404 | 10182928 | Continuous spikes and waves during sleep | glutamate ionotropic receptor NMDA type subunit 2A |
| GRIN2A | 16 | 9753404 | 10182928 | Early-onset epileptic encephalopathy and intellectual disability due to GRIN2A mutation | glutamate ionotropic receptor NMDA type subunit 2A |
| GRIN2A | 16 | 9753404 | 10182928 | Landau-Kleffner syndrome | glutamate ionotropic receptor NMDA type subunit 2A |
| GRIN2A | 16 | 9753404 | 10182928 | Rolandic epilepsy | glutamate ionotropic receptor NMDA type subunit 2A |
| GRIN2A | 16 | 9753404 | 10182928 | Rolandic epilepsy-speech dyspraxia syndrome | glutamate ionotropic receptor NMDA type subunit 2A |
| GRIN2A | 16 | 9753404 | 10182928 | EPILEPSY FOCAL WITH SPEECH DISORDER AND WITH OR WITHOUT IMPAIRED INTELLECTUAL DEVELOPMENT | glutamate ionotropic receptor NMDA type subunit 2A |
| GRIN2A | 16 | 9753404 | 10182928 | EPILEPSY FOCAL WITH SPEECH DISORDER AND WITH OR WITHOUT INTELLECTUAL DEVELOPMENTAL DISORDER | glutamate ionotropic receptor NMDA type subunit 2A |
| GRIN2A | 16 | 9753404 | 10182928 | Appendix Adenocarcinoma | glutamate ionotropic receptor NMDA type subunit 2A |
| GRIN2A | 16 | 9753404 | 10182928 | Atypical Meningioma | glutamate ionotropic receptor NMDA type subunit 2A |
| GRIN2A | 16 | 9753404 | 10182928 | B-cell acute lymphoblastic leukemia | glutamate ionotropic receptor NMDA type subunit 2A |
| GRIN2A | 16 | 9753404 | 10182928 | Bladder Adenocarcinoma | glutamate ionotropic receptor NMDA type subunit 2A |
| GRIN2A | 16 | 9753404 | 10182928 | Bladder Small Cell Neuroendocrine Carcinoma | glutamate ionotropic receptor NMDA type subunit 2A |
| GRIN2A | 16 | 9753404 | 10182928 | Brain Stem Glioblastoma | glutamate ionotropic receptor NMDA type subunit 2A |
| GRIN2A | 16 | 9753404 | 10182928 | Breast Carcinoma by Gene Expression Profile | glutamate ionotropic receptor NMDA type subunit 2A |
| GRIN2A | 16 | 9753404 | 10182928 | Burkitts lymphoma | glutamate ionotropic receptor NMDA type subunit 2A |
| GRIN2A | 16 | 9753404 | 10182928 | Endometrial Endometrioid Adenocarcinoma | glutamate ionotropic receptor NMDA type subunit 2A |
| GRIN2A | 16 | 9753404 | 10182928 | Gallbladder Small Cell Neuroendocrine Carcinoma | glutamate ionotropic receptor NMDA type subunit 2A |
| GRIN2A | 16 | 9753404 | 10182928 | Hepatobiliary Neoplasm | glutamate ionotropic receptor NMDA type subunit 2A |
| GRIN2A | 16 | 9753404 | 10182928 | Hodgkins lymphoma | glutamate ionotropic receptor NMDA type subunit 2A |
| GRIN2A | 16 | 9753404 | 10182928 | Invasive Breast Carcinoma | glutamate ionotropic receptor NMDA type subunit 2A |
| GRIN2A | 16 | 9753404 | 10182928 | MANTLE CELL LYMPHOMA | glutamate ionotropic receptor NMDA type subunit 2A |
| GRIN2A | 16 | 9753404 | 10182928 | Merkel cell skin cancer | glutamate ionotropic receptor NMDA type subunit 2A |
| GRIN2A | 16 | 9753404 | 10182928 | ND | glutamate ionotropic receptor NMDA type subunit 2A |
| GRIN2A | 16 | 9753404 | 10182928 | Ovarian Endometrioid Adenocarcinoma with Squamous Differentiation | glutamate ionotropic receptor NMDA type subunit 2A |
| GRIN2A | 16 | 9753404 | 10182928 | Pancreatic Acinar Cell Carcinoma | glutamate ionotropic receptor NMDA type subunit 2A |
| GRIN2A | 16 | 9753404 | 10182928 | Placental Choriocarcinoma | glutamate ionotropic receptor NMDA type subunit 2A |
| GRIN2A | 16 | 9753404 | 10182928 | Pleural Biphasic Mesothelioma | glutamate ionotropic receptor NMDA type subunit 2A |
| GRIN2A | 16 | 9753404 | 10182928 | Sarcomatoid Carcinoma | glutamate ionotropic receptor NMDA type subunit 2A |
| GRIN2A | 16 | 9753404 | 10182928 | Secretory Meningioma | glutamate ionotropic receptor NMDA type subunit 2A |
| GRIN2A | 16 | 9753404 | 10182928 | T-cell acute lymphoblastic leukemia | glutamate ionotropic receptor NMDA type subunit 2A |
| GRIN2A | 16 | 9753404 | 10182928 | Testicular Teratoma | glutamate ionotropic receptor NMDA type subunit 2A |
| GRIN2A | 16 | 9753404 | 10182928 | Thymic Undifferentiated Carcinoma | glutamate ionotropic receptor NMDA type subunit 2A |
| GRIN2A | 16 | 9753404 | 10182928 | Thyroid Gland Undifferentiated (Anaplastic) Carcinoma | glutamate ionotropic receptor NMDA type subunit 2A |
| GRIN2A | 16 | 9753404 | 10182928 | Acute lymphoblastic leukemia | glutamate ionotropic receptor NMDA type subunit 2A |
| GRIN2A | 16 | 9753404 | 10182928 | Acute myeloid leukemia | glutamate ionotropic receptor NMDA type subunit 2A |
| GRIN2A | 16 | 9753404 | 10182928 | adenosquamous lung carcinoma | glutamate ionotropic receptor NMDA type subunit 2A |
| GRIN2A | 16 | 9753404 | 10182928 | Anaplastic astrocytoma | glutamate ionotropic receptor NMDA type subunit 2A |
| GRIN2A | 16 | 9753404 | 10182928 | angiosarcoma | glutamate ionotropic receptor NMDA type subunit 2A |
| GRIN2A | 16 | 9753404 | 10182928 | Basal cell carcinoma | glutamate ionotropic receptor NMDA type subunit 2A |
| GRIN2A | 16 | 9753404 | 10182928 | bile duct carcinoma | glutamate ionotropic receptor NMDA type subunit 2A |
| GRIN2A | 16 | 9753404 | 10182928 | bladder transitional cell carcinoma | glutamate ionotropic receptor NMDA type subunit 2A |
| GRIN2A | 16 | 9753404 | 10182928 | brain glioblastoma | glutamate ionotropic receptor NMDA type subunit 2A |
| GRIN2A | 16 | 9753404 | 10182928 | Breast carcinoma | glutamate ionotropic receptor NMDA type subunit 2A |
| GRIN2A | 16 | 9753404 | 10182928 | breast ductal adenocarcinoma | glutamate ionotropic receptor NMDA type subunit 2A |
| GRIN2A | 16 | 9753404 | 10182928 | cecum adenocarcinoma | glutamate ionotropic receptor NMDA type subunit 2A |
| GRIN2A | 16 | 9753404 | 10182928 | cervical squamous cell carcinoma | glutamate ionotropic receptor NMDA type subunit 2A |
| GRIN2A | 16 | 9753404 | 10182928 | Chronic lymphocytic leukemia | glutamate ionotropic receptor NMDA type subunit 2A |
| GRIN2A | 16 | 9753404 | 10182928 | Clear cell renal carcinoma | glutamate ionotropic receptor NMDA type subunit 2A |
| GRIN2A | 16 | 9753404 | 10182928 | colon adenocarcinoma | glutamate ionotropic receptor NMDA type subunit 2A |
| GRIN2A | 16 | 9753404 | 10182928 | colonic neoplasm | glutamate ionotropic receptor NMDA type subunit 2A |
| GRIN2A | 16 | 9753404 | 10182928 | colorectal adenocarcinoma | glutamate ionotropic receptor NMDA type subunit 2A |
| GRIN2A | 16 | 9753404 | 10182928 | diffuse gastric adenocarcinoma | glutamate ionotropic receptor NMDA type subunit 2A |
| GRIN2A | 16 | 9753404 | 10182928 | diffuse large B-cell lymphoma | glutamate ionotropic receptor NMDA type subunit 2A |
| GRIN2A | 16 | 9753404 | 10182928 | Ependymoma | glutamate ionotropic receptor NMDA type subunit 2A |
| GRIN2A | 16 | 9753404 | 10182928 | gastric adenocarcinoma | glutamate ionotropic receptor NMDA type subunit 2A |
| GRIN2A | 16 | 9753404 | 10182928 | hairy cell leukemia | glutamate ionotropic receptor NMDA type subunit 2A |
| GRIN2A | 16 | 9753404 | 10182928 | head and neck squamous cell carcinoma | glutamate ionotropic receptor NMDA type subunit 2A |
| GRIN2A | 16 | 9753404 | 10182928 | Hepatocellular Carcinoma | glutamate ionotropic receptor NMDA type subunit 2A |
| GRIN2A | 16 | 9753404 | 10182928 | kidney neoplasm | glutamate ionotropic receptor NMDA type subunit 2A |
| GRIN2A | 16 | 9753404 | 10182928 | large cell lung carcinoma | glutamate ionotropic receptor NMDA type subunit 2A |
| GRIN2A | 16 | 9753404 | 10182928 | leiomyosarcoma | glutamate ionotropic receptor NMDA type subunit 2A |
| GRIN2A | 16 | 9753404 | 10182928 | lobular breast carcinoma | glutamate ionotropic receptor NMDA type subunit 2A |
| GRIN2A | 16 | 9753404 | 10182928 | Lung adenocarcinoma | glutamate ionotropic receptor NMDA type subunit 2A |
| GRIN2A | 16 | 9753404 | 10182928 | Lung carcinoma | glutamate ionotropic receptor NMDA type subunit 2A |
| GRIN2A | 16 | 9753404 | 10182928 | lymphoid neoplasm | glutamate ionotropic receptor NMDA type subunit 2A |
| GRIN2A | 16 | 9753404 | 10182928 | mast-cell leukemia | glutamate ionotropic receptor NMDA type subunit 2A |
| GRIN2A | 16 | 9753404 | 10182928 | Melanoma | glutamate ionotropic receptor NMDA type subunit 2A |
| GRIN2A | 16 | 9753404 | 10182928 | Multiple myeloma | glutamate ionotropic receptor NMDA type subunit 2A |
| GRIN2A | 16 | 9753404 | 10182928 | Neoplasm | glutamate ionotropic receptor NMDA type subunit 2A |
| GRIN2A | 16 | 9753404 | 10182928 | nodular melanoma | glutamate ionotropic receptor NMDA type subunit 2A |
| GRIN2A | 16 | 9753404 | 10182928 | non-small cell lung carcinoma | glutamate ionotropic receptor NMDA type subunit 2A |
| GRIN2A | 16 | 9753404 | 10182928 | oral squamous cell carcinoma | glutamate ionotropic receptor NMDA type subunit 2A |
| GRIN2A | 16 | 9753404 | 10182928 | OSTEOSARCOMA | glutamate ionotropic receptor NMDA type subunit 2A |
| GRIN2A | 16 | 9753404 | 10182928 | ovarian mucinous adenocarcinoma | glutamate ionotropic receptor NMDA type subunit 2A |
| GRIN2A | 16 | 9753404 | 10182928 | pancreatic carcinoma | glutamate ionotropic receptor NMDA type subunit 2A |
| GRIN2A | 16 | 9753404 | 10182928 | pancreatic ductal adenocarcinoma | glutamate ionotropic receptor NMDA type subunit 2A |
| GRIN2A | 16 | 9753404 | 10182928 | pancreatic neuroendocrine tumor | glutamate ionotropic receptor NMDA type subunit 2A |
| GRIN2A | 16 | 9753404 | 10182928 | pharyngeal squamous cell carcinoma | glutamate ionotropic receptor NMDA type subunit 2A |
| GRIN2A | 16 | 9753404 | 10182928 | POLYCYTHEMIA VERA | glutamate ionotropic receptor NMDA type subunit 2A |
| GRIN2A | 16 | 9753404 | 10182928 | prostate adenocarcinoma | glutamate ionotropic receptor NMDA type subunit 2A |
| GRIN2A | 16 | 9753404 | 10182928 | prostate carcinoma | glutamate ionotropic receptor NMDA type subunit 2A |
| GRIN2A | 16 | 9753404 | 10182928 | pulmonary blastoma | glutamate ionotropic receptor NMDA type subunit 2A |
| GRIN2A | 16 | 9753404 | 10182928 | rectal adenocarcinoma | glutamate ionotropic receptor NMDA type subunit 2A |
| GRIN2A | 16 | 9753404 | 10182928 | renal carcinoma | glutamate ionotropic receptor NMDA type subunit 2A |
| GRIN2A | 16 | 9753404 | 10182928 | Renal cell carcinoma | glutamate ionotropic receptor NMDA type subunit 2A |
| GRIN2A | 16 | 9753404 | 10182928 | Rhabdomyosarcoma | glutamate ionotropic receptor NMDA type subunit 2A |
| GRIN2A | 16 | 9753404 | 10182928 | salivary gland squamous cell carcinoma | glutamate ionotropic receptor NMDA type subunit 2A |
| GRIN2A | 16 | 9753404 | 10182928 | skin carcinoma | glutamate ionotropic receptor NMDA type subunit 2A |
| GRIN2A | 16 | 9753404 | 10182928 | small cell carcinoma | glutamate ionotropic receptor NMDA type subunit 2A |
| GRIN2A | 16 | 9753404 | 10182928 | Small cell lung carcinoma | glutamate ionotropic receptor NMDA type subunit 2A |
| GRIN2A | 16 | 9753404 | 10182928 | soft tissue sarcoma | glutamate ionotropic receptor NMDA type subunit 2A |
| GRIN2A | 16 | 9753404 | 10182928 | squamous cell lung carcinoma | glutamate ionotropic receptor NMDA type subunit 2A |
| GRIN2A | 16 | 9753404 | 10182928 | thyroid carcinoma | glutamate ionotropic receptor NMDA type subunit 2A |
| GRIN2A | 16 | 9753404 | 10182928 | thyroid neoplasm | glutamate ionotropic receptor NMDA type subunit 2A |
| GRIN2A | 16 | 9753404 | 10182928 | undifferentiated pleomorphic sarcoma | glutamate ionotropic receptor NMDA type subunit 2A |
| GRIN2A | 16 | 9753404 | 10182928 | urothelial carcinoma | glutamate ionotropic receptor NMDA type subunit 2A |
| PRKCA | 17 | 66302613 | 66810743 |  | protein kinase C alpha |
| COL1A1 | 17 | 50184101 | 50201632 | Arthrochalasia Ehlers-Danlos syndrome | collagen type I alpha 1 chain |
| COL1A1 | 17 | 50184101 | 50201632 | CAFFEY DISEASE | collagen type I alpha 1 chain |
| COL1A1 | 17 | 50184101 | 50201632 | Classical Ehlers-Danlos syndrome | collagen type I alpha 1 chain |
| COL1A1 | 17 | 50184101 | 50201632 | DERMATOFIBROSARCOMA PROTUBERANS | collagen type I alpha 1 chain |
| COL1A1 | 17 | 50184101 | 50201632 | Ehlers-Danlos/osteogenesis imperfecta syndrome | collagen type I alpha 1 chain |
| COL1A1 | 17 | 50184101 | 50201632 | High bone mass osteogenesis imperfecta | collagen type I alpha 1 chain |
| COL1A1 | 17 | 50184101 | 50201632 | Osteogenesis imperfecta type 1 | collagen type I alpha 1 chain |
| COL1A1 | 17 | 50184101 | 50201632 | Osteogenesis imperfecta type 2 | collagen type I alpha 1 chain |
| COL1A1 | 17 | 50184101 | 50201632 | Osteogenesis imperfecta type 3 | collagen type I alpha 1 chain |
| COL1A1 | 17 | 50184101 | 50201632 | Osteogenesis imperfecta type 4 | collagen type I alpha 1 chain |
| COL1A1 | 17 | 50184101 | 50201632 | COMBINED OSTEOGENESIS IMPERFECTA AND EHLERS-DANLOS SYNDROME 1 | collagen type I alpha 1 chain |
| COL1A1 | 17 | 50184101 | 50201632 | EHLERS-DANLOS SYNDROME ARTHROCHALASIA TYPE 1 | collagen type I alpha 1 chain |
| COL1A1 | 17 | 50184101 | 50201632 | OSTEOGENESIS IMPERFECTA TYPE I | collagen type I alpha 1 chain |
| COL1A1 | 17 | 50184101 | 50201632 | OSTEOGENESIS IMPERFECTA TYPE II | collagen type I alpha 1 chain |
| COL1A1 | 17 | 50184101 | 50201632 | OSTEOGENESIS IMPERFECTA TYPE III | collagen type I alpha 1 chain |
| COL1A1 | 17 | 50184101 | 50201632 | OSTEOGENESIS IMPERFECTA TYPE IV | collagen type I alpha 1 chain |
| COL1A1 | 17 | 50184101 | 50201632 | OSTEOPOROSIS | collagen type I alpha 1 chain |
| COL1A1 | 17 | 50184101 | 50201632 | COL1A1-RELATED OSTEOGENESIS IMPERFECTA | collagen type I alpha 1 chain |
| COL1A1 | 17 | 50184101 | 50201632 | COL1A1/2-Related Osteogenesis Imperfecta | collagen type I alpha 1 chain |
| COL1A1 | 17 | 50184101 | 50201632 | EHLERS-DANLOS SYNDROME TYPE VIIA | collagen type I alpha 1 chain |
| COL1A1 | 17 | 50184101 | 50201632 | EHLERS-DANLOS SYNDROME CLASSIC TYPE COL1A1-RELATED | collagen type I alpha 1 chain |
| COL1A1 | 17 | 50184101 | 50201632 | OSTEOGENESIS IMPERFECTA TYPE IIA | collagen type I alpha 1 chain |
| COL1A1 | 17 | 50184101 | 50201632 | Osteogenesis imperfecta type III | collagen type I alpha 1 chain |
| COL1A1 | 17 | 50184101 | 50201632 | B-cell acute lymphoblastic leukemia | collagen type I alpha 1 chain |
| COL1A1 | 17 | 50184101 | 50201632 | Brain Stem Glioblastoma | collagen type I alpha 1 chain |
| COL1A1 | 17 | 50184101 | 50201632 | Duodenal Adenocarcinoma | collagen type I alpha 1 chain |
| COL1A1 | 17 | 50184101 | 50201632 | Endometrial Endometrioid Adenocarcinoma | collagen type I alpha 1 chain |
| COL1A1 | 17 | 50184101 | 50201632 | Gallbladder Small Cell Neuroendocrine Carcinoma | collagen type I alpha 1 chain |
| COL1A1 | 17 | 50184101 | 50201632 | Hepatobiliary Neoplasm | collagen type I alpha 1 chain |
| COL1A1 | 17 | 50184101 | 50201632 | MANTLE CELL LYMPHOMA | collagen type I alpha 1 chain |
| COL1A1 | 17 | 50184101 | 50201632 | Merkel cell skin cancer | collagen type I alpha 1 chain |
| COL1A1 | 17 | 50184101 | 50201632 | ND | collagen type I alpha 1 chain |
| COL1A1 | 17 | 50184101 | 50201632 | Ovarian Endometrioid Adenocarcinoma | collagen type I alpha 1 chain |
| COL1A1 | 17 | 50184101 | 50201632 | Ovarian Endometrioid Adenocarcinoma with Squamous Differentiation | collagen type I alpha 1 chain |
| COL1A1 | 17 | 50184101 | 50201632 | Pancreatic Acinar Cell Carcinoma | collagen type I alpha 1 chain |
| COL1A1 | 17 | 50184101 | 50201632 | Parathyroid Gland Carcinoma | collagen type I alpha 1 chain |
| COL1A1 | 17 | 50184101 | 50201632 | Placental Choriocarcinoma | collagen type I alpha 1 chain |
| COL1A1 | 17 | 50184101 | 50201632 | Pleural Epithelioid Mesothelioma | collagen type I alpha 1 chain |
| COL1A1 | 17 | 50184101 | 50201632 | Acute myeloid leukemia | collagen type I alpha 1 chain |
| COL1A1 | 17 | 50184101 | 50201632 | adenosquamous lung carcinoma | collagen type I alpha 1 chain |
| COL1A1 | 17 | 50184101 | 50201632 | aneurysmal bone cyst | collagen type I alpha 1 chain |
| COL1A1 | 17 | 50184101 | 50201632 | Astrocytoma | collagen type I alpha 1 chain |
| COL1A1 | 17 | 50184101 | 50201632 | bile duct carcinoma | collagen type I alpha 1 chain |
| COL1A1 | 17 | 50184101 | 50201632 | bladder transitional cell carcinoma | collagen type I alpha 1 chain |
| COL1A1 | 17 | 50184101 | 50201632 | brain glioblastoma | collagen type I alpha 1 chain |
| COL1A1 | 17 | 50184101 | 50201632 | Breast carcinoma | collagen type I alpha 1 chain |
| COL1A1 | 17 | 50184101 | 50201632 | breast ductal adenocarcinoma | collagen type I alpha 1 chain |
| COL1A1 | 17 | 50184101 | 50201632 | cecum adenocarcinoma | collagen type I alpha 1 chain |
| COL1A1 | 17 | 50184101 | 50201632 | cervical squamous cell carcinoma | collagen type I alpha 1 chain |
| COL1A1 | 17 | 50184101 | 50201632 | Clear cell renal carcinoma | collagen type I alpha 1 chain |
| COL1A1 | 17 | 50184101 | 50201632 | colon adenocarcinoma | collagen type I alpha 1 chain |
| COL1A1 | 17 | 50184101 | 50201632 | colon carcinoma | collagen type I alpha 1 chain |
| COL1A1 | 17 | 50184101 | 50201632 | colorectal adenocarcinoma | collagen type I alpha 1 chain |
| COL1A1 | 17 | 50184101 | 50201632 | cutaneous fibrous histiocytoma | collagen type I alpha 1 chain |
| COL1A1 | 17 | 50184101 | 50201632 | Dedifferentiated liposarcoma | collagen type I alpha 1 chain |
| COL1A1 | 17 | 50184101 | 50201632 | diffuse gastric adenocarcinoma | collagen type I alpha 1 chain |
| COL1A1 | 17 | 50184101 | 50201632 | diffuse large B-cell lymphoma | collagen type I alpha 1 chain |
| COL1A1 | 17 | 50184101 | 50201632 | gastric adenocarcinoma | collagen type I alpha 1 chain |
| COL1A1 | 17 | 50184101 | 50201632 | hairy cell leukemia | collagen type I alpha 1 chain |
| COL1A1 | 17 | 50184101 | 50201632 | head and neck squamous cell carcinoma | collagen type I alpha 1 chain |
| COL1A1 | 17 | 50184101 | 50201632 | Hepatocellular Carcinoma | collagen type I alpha 1 chain |
| COL1A1 | 17 | 50184101 | 50201632 | kidney neoplasm | collagen type I alpha 1 chain |
| COL1A1 | 17 | 50184101 | 50201632 | large cell lung carcinoma | collagen type I alpha 1 chain |
| COL1A1 | 17 | 50184101 | 50201632 | leiomyosarcoma | collagen type I alpha 1 chain |
| COL1A1 | 17 | 50184101 | 50201632 | lobular breast carcinoma | collagen type I alpha 1 chain |
| COL1A1 | 17 | 50184101 | 50201632 | Lung adenocarcinoma | collagen type I alpha 1 chain |
| COL1A1 | 17 | 50184101 | 50201632 | Lung carcinoma | collagen type I alpha 1 chain |
| COL1A1 | 17 | 50184101 | 50201632 | lymphoid neoplasm | collagen type I alpha 1 chain |
| COL1A1 | 17 | 50184101 | 50201632 | Malignant peripheral nerve sheath tumor | collagen type I alpha 1 chain |
| COL1A1 | 17 | 50184101 | 50201632 | Melanoma | collagen type I alpha 1 chain |
| COL1A1 | 17 | 50184101 | 50201632 | neurofibroma | collagen type I alpha 1 chain |
| COL1A1 | 17 | 50184101 | 50201632 | nodular melanoma | collagen type I alpha 1 chain |
| COL1A1 | 17 | 50184101 | 50201632 | non-small cell lung carcinoma | collagen type I alpha 1 chain |
| COL1A1 | 17 | 50184101 | 50201632 | oral squamous cell carcinoma | collagen type I alpha 1 chain |
| COL1A1 | 17 | 50184101 | 50201632 | OSTEOSARCOMA | collagen type I alpha 1 chain |
| COL1A1 | 17 | 50184101 | 50201632 | ovarian carcinoma | collagen type I alpha 1 chain |
| COL1A1 | 17 | 50184101 | 50201632 | pancreatic carcinoma | collagen type I alpha 1 chain |
| COL1A1 | 17 | 50184101 | 50201632 | pancreatic ductal adenocarcinoma | collagen type I alpha 1 chain |
| COL1A1 | 17 | 50184101 | 50201632 | pancreatic neuroendocrine tumor | collagen type I alpha 1 chain |
| COL1A1 | 17 | 50184101 | 50201632 | prostate adenocarcinoma | collagen type I alpha 1 chain |
| COL1A1 | 17 | 50184101 | 50201632 | prostate carcinoma | collagen type I alpha 1 chain |
| COL1A1 | 17 | 50184101 | 50201632 | rectal adenocarcinoma | collagen type I alpha 1 chain |
| COL1A1 | 17 | 50184101 | 50201632 | schwannoma | collagen type I alpha 1 chain |
| COL1A1 | 17 | 50184101 | 50201632 | skin carcinoma | collagen type I alpha 1 chain |
| COL1A1 | 17 | 50184101 | 50201632 | Small cell lung carcinoma | collagen type I alpha 1 chain |
| COL1A1 | 17 | 50184101 | 50201632 | soft tissue sarcoma | collagen type I alpha 1 chain |
| COL1A1 | 17 | 50184101 | 50201632 | squamous cell lung carcinoma | collagen type I alpha 1 chain |
| COL1A1 | 17 | 50184101 | 50201632 | thyroid carcinoma | collagen type I alpha 1 chain |
| COL1A1 | 17 | 50184101 | 50201632 | undifferentiated pleomorphic sarcoma | collagen type I alpha 1 chain |
| KCNJ12 | 17 | 21376357 | 21419870 |  | potassium inwardly rectifying channel subfamily J member 12 |
| ITGB4 | 17 | 75721328 | 75757818 | Aplasia cutis congenita | integrin subunit beta 4 |
| ITGB4 | 17 | 75721328 | 75757818 | EPIDERMOLYSIS BULLOSA SIMPLEX WITH PYLORIC ATRESIA | integrin subunit beta 4 |
| ITGB4 | 17 | 75721328 | 75757818 | Intermediate generalized junctional epidermolysis bullosa | integrin subunit beta 4 |
| ITGB4 | 17 | 75721328 | 75757818 | Junctional epidermolysis bullosa with pyloric atresia | integrin subunit beta 4 |
| ITGB4 | 17 | 75721328 | 75757818 | Localized junctional epidermolysis bullosa | integrin subunit beta 4 |
| ITGB4 | 17 | 75721328 | 75757818 | EPIDERMOLYSIS BULLOSA JUNCTIONAL 5A INTERMEDIATE | integrin subunit beta 4 |
| ITGB4 | 17 | 75721328 | 75757818 | EPIDERMOLYSIS BULLOSA JUNCTIONAL 5B WITH PYLORIC ATRESIA | integrin subunit beta 4 |
| ITGB4 | 17 | 75721328 | 75757818 | Epidermolysis Bullosa Junctional | integrin subunit beta 4 |
| ITGB4 | 17 | 75721328 | 75757818 | Epidermolysis Bullosa Simplex Weber Cockayne | integrin subunit beta 4 |
| LAMA3 | 18 | 23689453 | 23956222 | Intermediate generalized junctional epidermolysis bullosa | laminin subunit alpha 3 |
| LAMA3 | 18 | 23689453 | 23956222 | Laryngo-onycho-cutaneous syndrome | laminin subunit alpha 3 |
| LAMA3 | 18 | 23689453 | 23956222 | Severe generalized junctional epidermolysis bullosa | laminin subunit alpha 3 |
| LAMA3 | 18 | 23689453 | 23956222 | EPIDERMOLYSIS BULLOSA JUNCTIONAL 1B SEVERE | laminin subunit alpha 3 |
| LAMA3 | 18 | 23689453 | 23956222 | EPIDERMOLYSIS BULLOSA JUNCTIONAL 2A INTERMEDIATE | laminin subunit alpha 3 |
| LAMA3 | 18 | 23689453 | 23956222 | EPIDERMOLYSIS BULLOSA JUNCTIONAL 2B SEVERE | laminin subunit alpha 3 |
| LAMA3 | 18 | 23689453 | 23956222 | EPIDERMOLYSIS BULLOSA JUNCTIONAL 2C LARYNGOONYCHOCUTANEOUS | laminin subunit alpha 3 |
| LAMA3 | 18 | 23689453 | 23956222 | LARYNGOONYCHOCUTANEOUS SYNDROME | laminin subunit alpha 3 |
| DLGAP1 | 18 | 3496032 | 4455307 |  | DLG associated protein 1 |
| PTPRS | 19 | 5158495 | 5340812 |  | protein tyrosine phosphatase receptor type S |
| COL9A3 | 20 | 62816244 | 62841159 | Autosomal recessive Stickler syndrome | collagen type IX alpha 3 chain |
| COL9A3 | 20 | 62816244 | 62841159 | Multiple epiphyseal dysplasia due to collagen 9 anomaly | collagen type IX alpha 3 chain |
| COL9A3 | 20 | 62816244 | 62841159 | EPIPHYSEAL DYSPLASIA MULTIPLE 3 | collagen type IX alpha 3 chain |
| COL9A3 | 20 | 62816244 | 62841159 | INTERVERTEBRAL DISC DISEASE | collagen type IX alpha 3 chain |
| COL9A3 | 20 | 62816244 | 62841159 | STICKLER SYNDROME TYPE VI | collagen type IX alpha 3 chain |
| COL9A3 | 20 | 62816244 | 62841159 | MULTIPLE EPIPHYSEAL DYSPLASIA TYPE 3 | collagen type IX alpha 3 chain |
| COL9A3 | 20 | 62816244 | 62841159 | Stickler syndrome | collagen type IX alpha 3 chain |
| DLGAP4 | 20 | 36306336 | 36528637 |  | DLG associated protein 4 |
| PLCB1 | 20 | 8077251 | 8968360 | Infantile spasms syndrome | phospholipase C beta 1 |
| PLCB1 | 20 | 8077251 | 8968360 | Malignant migrating focal seizures of infancy | phospholipase C beta 1 |
| PLCB1 | 20 | 8077251 | 8968360 | DEVELOPMENTAL AND EPILEPTIC ENCEPHALOPATHY 12 | phospholipase C beta 1 |
| PLCB1 | 20 | 8077251 | 8968360 | Early infantile epileptic encephalopathy 12 | phospholipase C beta 1 |
| GRIK1 | 21 | 29536933 | 29940033 |  | glutamate ionotropic receptor kainate type subunit 1 |
| CACNA1I | 22 | 39570753 | 39689735 | Autosomal dominant non-syndromic intellectual disability | calcium voltage-gated channel subunit alpha1 I |
| CACNA1I | 22 | 39570753 | 39689735 | NEURODEVELOPMENTAL DISORDER WITH SPEECH IMPAIRMENT AND WITH OR WITHOUT SEIZURES | calcium voltage-gated channel subunit alpha1 I |
| TSPO | 22 | 43151547 | 43163242 |  | translocator protein |
| LARGE1 | 22 | 33162226 | 33922841 | Congenital muscular dystrophy with intellectual disability | LARGE xylosyl- and glucuronyltransferase 1 |
| LARGE1 | 22 | 33162226 | 33922841 | Muscle-eye-brain disease | LARGE xylosyl- and glucuronyltransferase 1 |
| LARGE1 | 22 | 33162226 | 33922841 | Walker-Warburg syndrome | LARGE xylosyl- and glucuronyltransferase 1 |
| LARGE1 | 22 | 33162226 | 33922841 | MUSCULAR DYSTROPHY-DYSTROGLYCANOPATHY CONGENITAL WITH BRAIN AND EYE ANOMALIES TYPE A 1 | LARGE xylosyl- and glucuronyltransferase 1 |
| LARGE1 | 22 | 33162226 | 33922841 | MUSCULAR DYSTROPHY-DYSTROGLYCANOPATHY CONGENITAL WITH BRAIN AND EYE ANOMALIES TYPE A 6 | LARGE xylosyl- and glucuronyltransferase 1 |
| LARGE1 | 22 | 33162226 | 33922841 | MUSCULAR DYSTROPHY-DYSTROGLYCANOPATHY CONGENITAL WITH IMPAIRED INTELLECTUAL DEVELOPMENT TYPE B 6 | LARGE xylosyl- and glucuronyltransferase 1 |
| LARGE1 | 22 | 33162226 | 33922841 | MUSCULAR DYSTROPHY-DYSTROGLYCANOPATHY CONGENITAL WITH INTELLECTUAL DEVELOPMENTAL DISORDER TYPE B6 | LARGE xylosyl- and glucuronyltransferase 1 |
| CACNA1C | HG1815_PATCH | 312916 | 1046066 | BRUGADA SYNDROME 3 | calcium voltage-gated channel subunit alpha1 C |
| CACNA1C | HG1815_PATCH | 312916 | 1046066 | Long QT syndrome 8 | calcium voltage-gated channel subunit alpha1 C |
| CACNA1C | HG1815_PATCH | 312916 | 1046066 | Neurodevelopmental disorder with hypotonia language delay and skeletal defects with or without seizures | calcium voltage-gated channel subunit alpha1 C |
| CACNA1C | HG1815_PATCH | 312916 | 1046066 | TIMOTHY SYNDROME | calcium voltage-gated channel subunit alpha1 C |
| CACNA1C | HG1815_PATCH | 312916 | 1046066 | CACNA1C-related Timothy syndrome | calcium voltage-gated channel subunit alpha1 C |
| CACNA1C | HG1815_PATCH | 312916 | 1046066 | BRUGADA SYNDROME 3 | CACNA1C intronic transcript 2 |
| CACNA1C | HG1815_PATCH | 312916 | 1046066 | Long QT syndrome 8 | CACNA1C intronic transcript 2 |
| CACNA1C | HG1815_PATCH | 312916 | 1046066 | Neurodevelopmental disorder with hypotonia language delay and skeletal defects with or without seizures | CACNA1C intronic transcript 2 |
| CACNA1C | HG1815_PATCH | 312916 | 1046066 | TIMOTHY SYNDROME | CACNA1C intronic transcript 2 |
| CACNA1C | HG1815_PATCH | 312916 | 1046066 | CACNA1C-related Timothy syndrome | CACNA1C intronic transcript 2 |
| KCNQ1 | HSCHR11_1_CTG7 | 1031 | 81275 | ATRIAL FIBRILLATION FAMILIAL 3 | potassium voltage-gated channel subfamily Q member 1 |
| KCNQ1 | HSCHR11_1_CTG7 | 1031 | 81275 | BECKWITH-WIEDEMANN SYNDROME | potassium voltage-gated channel subfamily Q member 1 |
| KCNQ1 | HSCHR11_1_CTG7 | 1031 | 81275 | JERVELL AND LANGE-NIELSEN SYNDROME 1 | potassium voltage-gated channel subfamily Q member 1 |
| KCNQ1 | HSCHR11_1_CTG7 | 1031 | 81275 | LONG QT SYNDROME 1 | potassium voltage-gated channel subfamily Q member 1 |
| KCNQ1 | HSCHR11_1_CTG7 | 1031 | 81275 | SHORT QT SYNDROME 2 | potassium voltage-gated channel subfamily Q member 1 |
| KCNQ1 | HSCHR11_1_CTG7 | 1031 | 81275 | Jervell and Lange-Nielsen syndrome type 1 | potassium voltage-gated channel subfamily Q member 1 |
| KCNQ1 | HSCHR11_1_CTG7 | 1031 | 81275 | KCNQ1-related JLNS | potassium voltage-gated channel subfamily Q member 1 |
| KCNQ1 | HSCHR11_1_CTG7 | 1031 | 81275 | KCNQ1-related LQTS | potassium voltage-gated channel subfamily Q member 1 |
| KCNQ1 | HSCHR11_1_CTG7 | 1031 | 81275 | KCNQ1-related SQTS | potassium voltage-gated channel subfamily Q member 1 |
| AP2A2 | HSCHR11_2_CTG1 | 3478 | 74982 |  | adaptor related protein complex 2 subunit alpha 2 |
| AP2A2 | HSCHR11_3_CTG1 | 3478 | 81830 |  | adaptor related protein complex 2 subunit alpha 2 |
| APBA2 | HSCHR15_4_CTG8 | 1194322 | 1393362 |  | amyloid beta precursor protein binding family A member 2 |
| CHRNA7 | HSCHR15_4_CTG8 | 4315611 | 4454253 | CHROMOSOME 15q13.3 DELETION SYNDROME | cholinergic receptor nicotinic alpha 7 subunit |
| CHRNA7 | HSCHR15_6_CTG8 | 359628 | 497696 | CHROMOSOME 15q13.3 DELETION SYNDROME | cholinergic receptor nicotinic alpha 7 subunit |
| UGT2A3 | HSCHR4_1_CTG9 | 504482 | 527810 |  | UDP glucuronosyltransferase family 2 member A3 |
| SLC6A3 | HSCHR5_3_CTG1 | 64343 | 121219 | PARKINSONISM-DYSTONIA 1 INFANTILE-ONSET | solute carrier family 6 member 3 |
| SLC6A3 | HSCHR5_3_CTG1 | 64343 | 121219 | TOBACCO ADDICTION SUSCEPTIBILITY TO | solute carrier family 6 member 3 |
| SLC6A3 | HSCHR5_3_CTG1 | 64343 | 121219 | Infantile dystonia-parkinsonism | solute carrier family 6 member 3 |
| ADAMTS12 | HSCHR5_6_CTG1 | 41479 | 93514 |  | ADAM metallopeptidase with thrombospondin type 1 motif 12 |
| FLOT1 | HSCHR6_MHC_APD_CTG1 | 2057212 | 2060006 |  | flotillin 1 |
| FLOT1 | HSCHR6_MHC_COX_CTG1 | 2207446 | 2222473 |  | flotillin 1 |
| FLOT1 | HSCHR6_MHC_DBB_CTG1 | 1983559 | 1998584 |  | flotillin 1 |
| FLOT1 | HSCHR6_MHC_MANN_CTG1 | 2037960 | 2052984 |  | flotillin 1 |
| FLOT1 | HSCHR6_MHC_MCF_CTG1 | 2071784 | 2085614 |  | flotillin 1 |
| FLOT1 | HSCHR6_MHC_QBL_CTG1 | 1982822 | 1997843 |  | flotillin 1 |
| FLOT1 | HSCHR6_MHC_SSTO_CTG1 | 2028495 | 2043529 |  | flotillin 1 |
